# Self-Sorting Governed by Chelate Cooperativity

**DOI:** 10.1021/jacs.1c13295

**Published:** 2022-03-19

**Authors:** David Serrano-Molina, Carlos Montoro-García, María J. Mayoral, Alberto de Juan, David González-Rodríguez

**Affiliations:** † Nanostructured Molecular Systems and Materials Group, Departamento de Química Orgánica, Facultad de Ciencias, Universidad Autónoma de Madrid, 28049 Madrid, Spain; ‡ Departamento de Química Inorgánica, Facultad de Ciencias Químicas, Universidad Complutense de Madrid, 28040 Madrid, Spain; § Institute for Advanced Research in Chemical Sciences (IAdChem), Universidad Autónoma de Madrid, 28049 Madrid, Spain

## Abstract

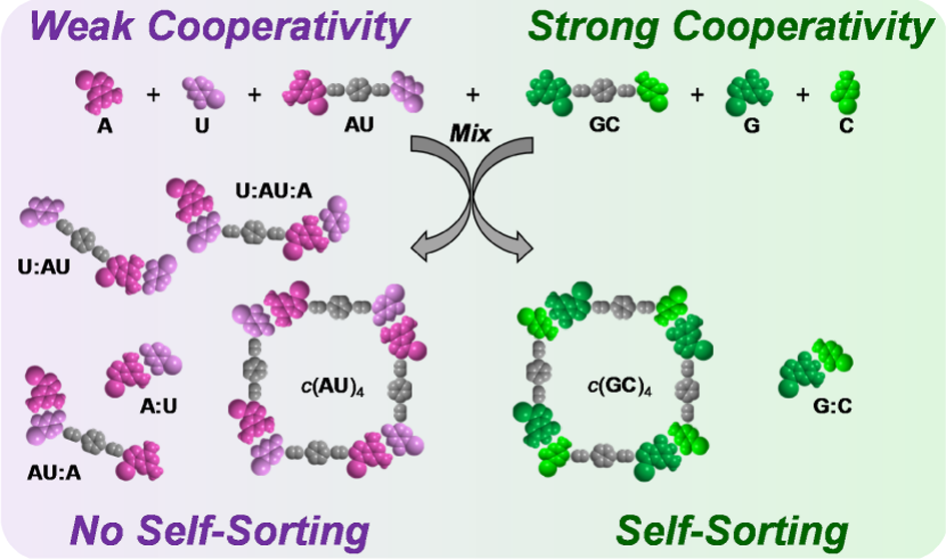

Self-sorting phenomena are the basis of manifold relevant (bio)chemical processes where a set of molecules is able to interact with no interference from other sets and are ruled by a number of codes that are programmed in molecular structures. In this work, we study, the relevance of chelate cooperativity as a code for achieving high self-sorting fidelities. In particular, we establish qualitative and quantitative relationships between the cooperativity of a cyclic system and the self-sorting fidelity when combined with other molecules that share identical geometry and/or binding interactions. We demonstrate that only systems displaying sufficiently strong chelate cooperativity can achieve quantitative narcissistic self-sorting fidelities either by dictating the distribution of cyclic species in complex mixtures or by ruling the competition between the intra- and intermolecular versions of a noncovalent interaction.

## Introduction

As supramolecular chemistry evolves to the generation of systems of increasing complexity that often mimic certain characteristics of biological processes,^1−4^ the study and comprehension of the performance of interacting mixtures of multiple compounds becomes essential. This is illustrated in natural systems through several levels of compartmentalization that allow multiple self-assembled machineries to operate simultaneously and orthogonally with precise spatial and temporal control.^5^*Self-sorting* arises in this context as a key concept to define the collective behavior of a set of molecules able to form a specific assembly with high fidelity and no interference with the rest.^6−12^ Self-sorting is the basis of relevant chemical processes like phase separation, kinetic resolution, or self-replication and can be *narcissistic* if a molecule has a strong tendency for self-recognition and hence binds its own kind, or *social*, if such a molecule undergoes a self-discrimination process and instead shows a high affinity for others.

Self-sorting is governed by a number of “codes” existing in chemically programmed molecules that determine recognition or discrimination phenomena.^7^ Geometric complementarity is an important one and dictates that any interacting set of molecules must have matching size and shape to maximize such an interaction. Another essential code resides in the nature of the functional groups present in a molecule that give rise to specific noncovalent interactions (H-bonding, metal–ligand, van der Waals, π–π stacking, dipole–dipole, etc.) and thus determine its affinity for others. Not only that, some noncovalent forces allow for a subset of codes that additionally influence intermolecular interactions. Some examples are the match between donor (*D*) and acceptor (*A*) groups in complementary H-bonding fragments,^13−15^ the modulation of the coordination number and geometry as a function of the nature of metals and ligands,^16^ the preference for donor–acceptor stacking interactions between electron-rich and electron-poor π-conjugated molecules,^17,18^ the introduction of steric effects in closely interacting environments,^19−21^ or the use of guests containing multiple binding epitopes to control the kinetic and thermodynamic outcomes of multicomponent systems.^22^ Even chirality can reliably function as a self-sorting code.^23,24^ Furthermore, the outcome of self-sorting phenomena, particularly in complex mixtures, is frequently the result of the combination of several codes. For instance, the formation of a specific DNA duplex from a mixture of single strands with diverse sequences is primarily a result of both a geometric and a H-bonding pattern match. The two codes combined lead to stabilization increments whenever G:C and A:T purine:pyrimidine contacts are established along the double strand.^25^

However, although it is quite evident that a strong influence must exist and many reported cyclic assemblies clearly profit from it,^26^ little is known about the relationship between self-sorting and cooperativity. *Cooperativity* is responsible for the difference between the energy of a self-assembled system as a whole and that expected from the sum of the individual isolated interactions. It is intuitive to think that, in many cases, a supramolecular process of positive cooperativity would enhance self-recognition, whereas negative cooperativity would favor self-discrimination. Hence, cooperativity, in its various forms,^27−30^ should be regarded as an additional powerful and programmable code to rule the fidelity of self-sorting processes and this is what we demonstrate herein. In this work, we establish qualitative and quantitative relationships between the *chelate cooperativity* of a H-bonded macrocyclic system,^31−33^ quantified by the product *K*·EM (*K* = association constant; EM = effective molarity), and the self-sorting fidelity when combined with other molecules that share identical geometry and/or binding interactions.

**Figure 1:**
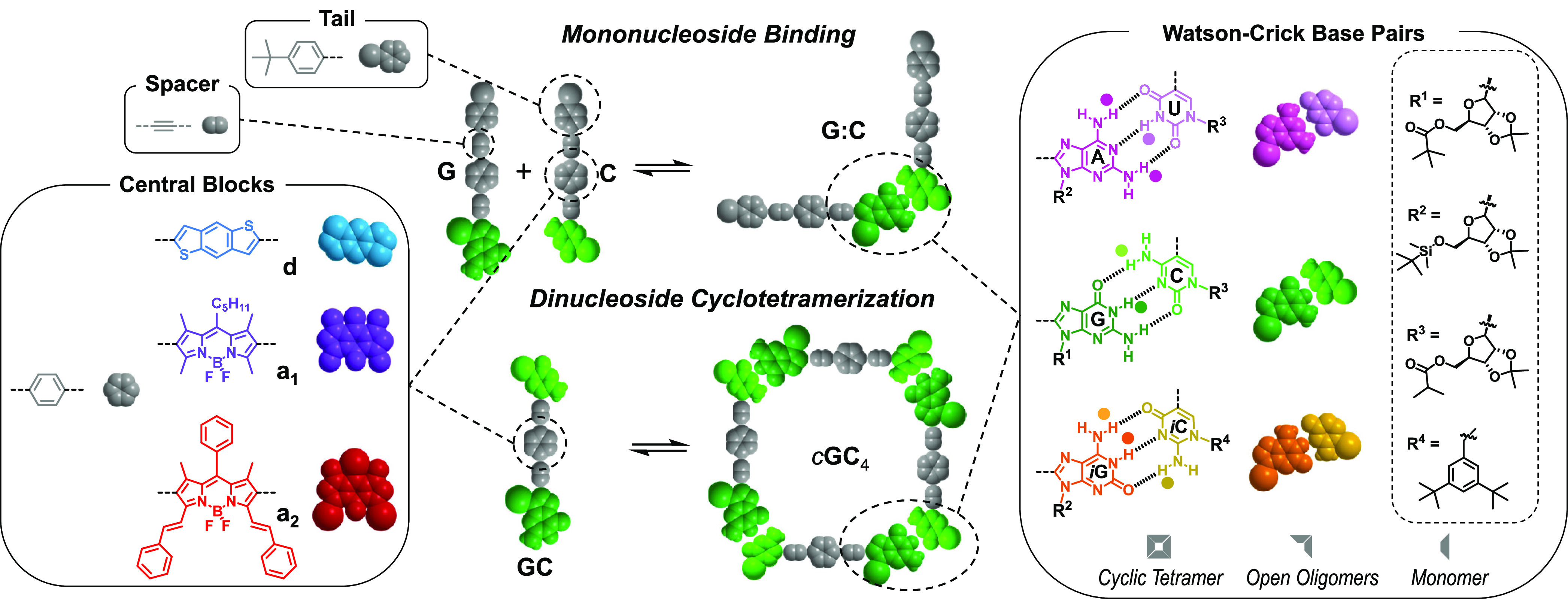
Schematic representation of the mono- and dinucleoside molecules employed in this work to assess self-sorting phenomena, comprising different terminal nucleobases and central blocks (see the Supporting Information. for full details on the molecular structure and characterization). Proton nuclear magnetic resonance (^1^H NMR) signals will be labeled in this work by a color code (type of proton) and a shape code (type of supramolecular species: cyclic tetramer, open oligomer, or monomer).

The noncovalent interaction used in our system will be the complementary triple H-bonding between purine and pyrimidine nucleobases (Figure 1), namely, guanine (G), 2-aminoadenine (abbreviated here as A), *iso*guanine (*i*G), cytosine (C), uracil (U), and *iso*cytosine (*i*C). The association constants (*K*) between complementary purine:pyrimidine pairs are well-known in the literature^34^ and have been specifically measured by us in chloroform (*ca. K*_G:C_ ∼ *K*_*i*G:*i*C_ = 2 × 10^4^ M^–1^; *K*_A:U_ = 3 × 10^2^ M^–1^),^35^ a chloroform–carbon tetrachloride 2:3 mixture (*ca. K*_A:U_ = 3 × 10^3^ M^–1^), and toluene (*ca. K*_G:C_ ∼ *K*_*i*G:*i*C_ = 3 × 10^5^ M^–1^; *K*_A:U_ = 5 × 10^3^ M^–1^)^36^ for the lipophilic nucleosides used in this work. As a consequence of their *DAD*–*ADA* H-bonding pattern, the A:U association constant is considerably lower than G:C and *i*G:*i*C, which bind through *DDA*–*AAD* and *ADD*–*DAA* patterns. It must be noted that the reverse Watson–Crick G:*i*C and *i*G:C pairs are also complementary and bind with similar H-bonding strength than the canonical G:C and *i*G:*i*C pairs (see Figure S1A).^35^

On the other hand, the cooperative process employed herein will be the cyclotetramerization of G–C, A–U, and iG–iC dinucleoside monomers, which are prepared by coupling these complementary purine–pyrimidine bases at the termini of linear π-conjugated spacers (Figure 1).^33^ The establishment of Watson–Crick triple H-bonding interactions between the edges of these dinucleoside molecules disposes the pyrimidine 5- and purine 8-positions in a 90° angle, which results in the assembly of unstrained cyclic tetramers with high fidelity. This supramolecular process has been studied by us during the last few years and has been the basis of systems showing record chelate cooperativities,^37−40^ nanostructured monolayers with well-defined cavities,^41^ highly efficient reversible dispersing agents for carbon nanostructures,^42^ or nanotubes self-assembled in organic^43^ or aqueous^44^ environments. It was demonstrated that **AU** cyclic tetramers (*c*(**AU**)_4_) showed EM values (EM_AU_ ∼ 10^–1^–10^–2^ M) that are at least three orders of magnitude lower than those exhibited by **GC** or ***i*****G*****i*****C** macrocycles (*c*(**GC**)_4_ and *c*(***i*****G*****i*****C**)_4_; EM_GC_ ∼ EM_*i*G*i*C_ ∼ 10^2^–10^3^ M), which was ascribed to entropic effects related to the number of degrees of freedom that are lost upon cyclization.^45^ The cyclotetramerization constants (*K*_C_) for any given cyclic tetramer can thus be estimated as *K*_C_ = *K*^4^·EM using the reported and previously mentioned *K*_G:C_, *K*_*i*G:*i*C_, and *K*_A:U_ reference association constants and EM_GC_, EM_*i*G*i*C_, and EM_AU_ effective molarity values. Taking CHCl_3_ as a reference solvent, these numbers lead to *K·*EM products as high as *ca.* 10^6^–10^7^ for *c*(**GC**)_4_ and *c*(***i*****G*****i*****C**)_4_, and as low as *ca.* 1–10 for *c*(**AU**)_4_.

Self-sorting fidelity will be experimentally assessed in this work by means of two techniques that employ different kinds of molecules (Figure 1) and operate under different conditions. First, we will study self-sorting phenomena by a combination of NMR experiments within the 10^–1^–10^–4^ M range. To this end, mono- and dinucleoside monomers with a *p*-phenylene central block will be employed.^37,45^ Second, and in a complementary manner, we will make use of circular dichroism (CD) and emission spectroscopy in the 10^–4^–10^–6^ M range. Since concentration is lowered considerably, we will generally use the nonpolar toluene solvent, where association constants between nucleobases is enhanced^36^ to maintain a high population of associated species. In particular, the occurrence, or not, of Förster resonance energy transfer (FRET) processes between donor and acceptor dye labeled molecules.^36,46^ To this end, a battery of mono- and dinucleoside molecules, equipped with linearly substituted donor bithiophene or acceptor BODIPY dyes (**d**, **a**_**1**_ or **a**_**2**_ in Figure 1), will be employed. These dyes were selected taking into account: (1) their identical length, so that the formation of mixed cyclic assemblies remains possible and self-sorting is not driven by geometric codes and (2) their complementary absorption and emission features, so that they constitute two couples of FRET donors and acceptors. It is important to note that the EM values of the macrocycles bearing these π-functional units do not change significantly with respect to those calculated for a *p*-phenylene central block, as demonstrated earlier,^46^ since these BODIPY and bithiophene fragments are rigid and do not bring new conformational possibilities. Even if the bulky peripheral substituents can efficiently avoid the unspecific aggregation of these larger π-conjugated monomers, we took care to work in a solvent-concentration experimental window in which such further aggregation is not observed. Unfortunately, mass spectrometry (MS) measurements are not very useful to study this kind of mixtures. Even in the case of the most stable individual H-bonded complexes, MS spectra display multiple fragmentation peaks and provide a supramolecular picture that does not reflect properly the situation in solution.^37^ The structure of all molecules used in this work is shown in Figure S0, while their synthetic and characterization details can be found in the Supporting Information or in our previous work.^35−37,45,46^

Finally, we will demonstrate the strong authority of chelate cooperativity on self-sorting phenomena in two different situations: (1) mixtures of dinucleosides sharing the same geometry but with different complementary base pairs and (2) mixtures of dinucleosides and mononucleosides that share the same Watson–Crick complementary interaction.

## Results and Discussion

### Self-Sorting in Mixtures of Dinucleosides Sharing the Same Geometry

**Figure 2:**
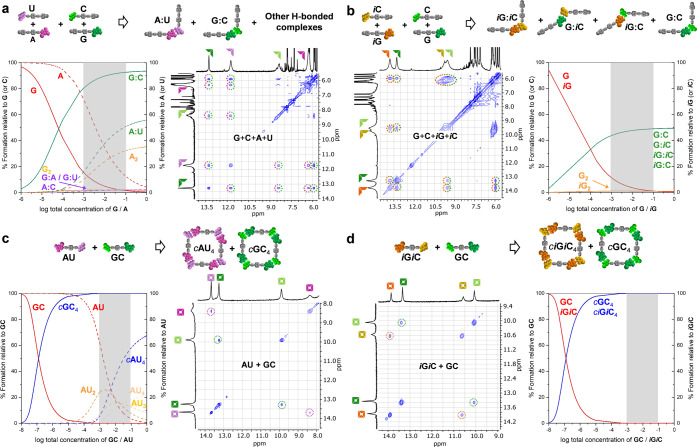
Speciation curves and downfield region of the NOESY spectra of (a) a 1:1:1:1 mixture of **G** + **C** + **A** + **U** (CDCl_3_; 10^–2^ M; 298 K), (b) a 1:1:1:1 mixture of **G** + **C** + ***i*****G** + ***i*****C** (CDCl_3_; 10^–2^ M; 298 K), (c) a 1:1 mixture of **AU** + **GC** (CDCl_3_/CCl_4_ 2:3; 10^–2^ M; 253 K), and (d) a 1:1 mixture of ***i*****G*****i*****C** + **GC** (THF-D_8_; 10^–2^ M; 298 K). These NMR solvents were chosen either to (c) maintain a high association constant (*K*) between the corresponding Watson–Crick pairs and thus a high population of associated species, or (d) to conveniently dissolve the mixtures (see Figure S2C for more details). For proton NMR codes, see Figure 1. Speciation curves were simulated using reported association constants and effective molarities (see Section S1).^37,45^

In the first situation, we compared the supramolecular behavior of mixtures of dinucleosides, able to assemble in cyclic tetramers,^45^ and the corresponding mixtures of mononucleosides, which are expected to bind in complementary purine:pyrimidine pairs as a function of their H-bonding patterns (Figure 1). We started examining the NMR spectra of 1:1 mixtures of complementary mononucleosides (**G** + **C**, **A** + **U**, and ***i*****G** + **iC**), where H-bonding formation is evidenced in the downfield shifts and NOESY cross-peaks between the relevant protons (Figure S2A). When combining these mononucleoside pairs in quaternary 1:1:1:1 mixtures (for instance: **G** + **C** + **A** + **U** or **G** + **C** + ***i*****G** + ***i*****C**; see Figure S2A), only minor changes were detected in the ^1^H NMR spectra. However, NOESY experiments displayed cross-peaks between multiple pairs, which suggests the absence of self-sorting phenomena (Figure 2a,b). This is not surprising for the **G** + **C** + ***i*****G** + ***i*****C** mixture, which exhibited cross-peaks between all possible combinations of regular (**G**:**C**, ***i*****G**:***i*****C**) and reverse (**G**:***i*****C**, ***i*****G**:**C**) Watson–Crick pairs (see Figure S1A), as well as between **G** and ***i*****G**. However, the **G** + **C** + **A** + **U** mixture also displayed cross-peaks between all possible pairs (**G**:**C**, **A**:**U**, and weaker **G**:**U**, **A**:**C**, **G**:**A**, and **C**:**U** cross-peaks), which indicates that the complementary nature of the diverse H-bonding fragments is not enough to generate strong recognition or discrimination phenomena between nucleosides under these particular conditions.

We then turned our attention to the behavior of 1:1 mixtures of dinucleosides (**GC** + **AU** or **GC** + ***i*****G*****i*****C**) in similar experimental conditions. The **GC** + **AU** combination, comprising two dinucleosides with orthogonal complementary pairs, displayed no change in their ^1^H NMR spectra upon mixing, and NOESY experiments clearly revealed that G only binds to C, while A only binds to U (Figures 2c and S2B). Therefore, in contrast to the corresponding mononucleosides, the **GC** + **AU** dinucleoside mixture exhibits strong narcissistic self-sorting in the corresponding cyclic tetramers *c*(**GC**)_4_ and *c*(**AU**)_4_, which is also what we would expect in view of the H-bonding pattern of the two Watson–Crick pairs involved. Now, in the case of the **GC** + ***i*****G*****i*****C** combination, G:C/*i*G:*i*C Watson–Crick and G:*i*C/*i*G:C reverse Watson–Crick pairs may be formed with virtually identical association strength (see Figure S1A), which would lead to a complex mixture of cyclic and open oligomers. However, ^1^H and NOESY NMR spectra (Figures 2d and S2B) clearly showed again that only the corresponding tetrameric cycles (*c*(**GC**)_4_ and *c*(***i*****G*****i*****C**)_4_) are formed, where G only binds to C, whereas *i*G binds exclusively to *i*C, and G:*i*C or *i*G:C cross-peaks are not detected. Therefore, the high propensity of each dinucleoside to form the respective cyclic tetramer with high cooperativities, in which G:C and *i*G:*i*C (or A:U) Watson–Crick interactions are demanded, rules narcissistic self-sorting here. Unfortunately, as described in Section S2 (see Figure S2C), **AU** + ***i*****G*****i*****C** mixtures could not be studied because we were not able to find a common solvent that provided at the same time sufficient stability for the *c*(**AU**)_4_ cycle and sufficient solubility for the *c*(***i*****G*****i*****C**)_4_ assembly under the experimental NMR conditions.

This supramolecular scenario can be modeled through speciation curves (Figure 2a–d, more details can be found in the Supporting Information, Section S1) in which the relative abundance of the different possible species in solution is represented as a function of the overall concentration. First, when comparing quaternary mixtures of mononucleosides, it is clear that the orthogonality of the Watson–Crick complementary H-bonding patterns is decisive to achieve relatively high self-sorting fidelities, which are represented by the ratio of the concentration of a molecule in a target associated species and the sum of the concentrations of such molecules in all possible species.^47^ For instance, in the **G** + **C** + **A** + **U** mixture (Figure 2a) at the ^1^H NMR concentration range (10^–1^–10^–3^ M; marked with a gray-shadowed area), the abundance of the **G** molecule in the **G**:**C** pair represents about 90% of all associated species in which this mononucleoside is present, the others being the **G**_2_ dimer and noncomplementary **G**:**U** and **G**:**A** pairs, whose abundance is about 2–4% each. Due to their weaker association, **A**:**U** complexes are less abundant in these conditions. In contrast, in the **G** + **C** + ***i*****G** + ***i*****C** mixture (Figure 2b), the **G** molecule is shared in equal amounts by the **G**:**C** and **G**:***i*****C** complexes, due to the virtually identical association strength of these pairs.^35^

**Figure 3:**
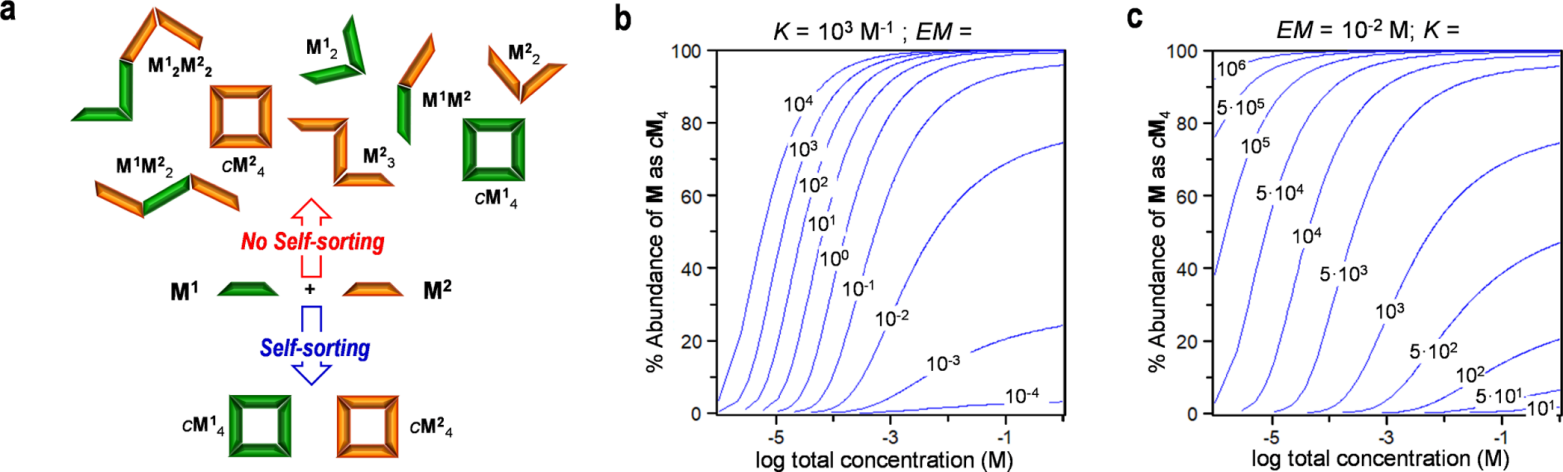
(a) Hypothetic situation in which two monomers (**M**^**1**^ and **M**^**2**^) are mixed that are endowed with complementary binding units at the edges, similar to **GC** + ***i*****G*****i*****C**. Each monomer can form linear supramolecular oligomers with itself or with the other with an identical association constant *K*. In addition, each monomer can self-associate into cyclic tetramer species with identical effective molarity EM. Narcissistic self-sorting occurs when **M**^**1**^ and **M**^**2**^ exclusively self-associate into cyclic *c***M**_4_^**1**^ and *c***M**_4_^**2**^ species. (b, c) Relationship between self-sorting fidelity (% relative abundance of **M**^**1**^ (or **M**^**2**^) in the cyclic *c***M**_4_^**1**^ (or *c***M**_4_^**2**^) species) as a function of total concentration: (b) cyclotetramerization EM of *c***M**_4_^**1**^ (or *c***M**_4_^**2**^) at a fixed *K* = 10^3^ M^–1^, or (c) association constant *K* between **M**^**1**^ and/or **M**^**2**^ fixing EM at 10^–2^ M.

Turning our attention to the dinucleosides, it is clear from the simulations that the moment that at least one of these molecules is able to cyclize with strong cooperativities, such cycle becomes fully populated and narcissistic self-sorting is complete under association conditions. This is observed for both the **GC** + **AU** mixture (Figure 2c) and the **GC** + ***i*****G*****i*****C** combination (Figure 2d), so it is independent on the orthogonality of the H-bonding patterns. If chelate cooperativity is not high enough, however, self-sorting is drastically reduced. Figure 3 shows how self-sorting fidelity depends on chelate cooperativity by simulating hypothetic situations, considering a similar mixture of **GC** + ***i*****G*****i*****C** but with varying EM (Figure 3b) and *K* (Figure 3c) values for both cycles. In each case, the other thermodynamic parameter (*K* or EM, respectively) was arbitrarily fixed at *K* = 10^3^ M^–1^ and EM = 10^–2^ M, since these are very typical values found for cyclic assemblies in solution. It is clear that self-sorting fidelity is close to 100% over a wider range of concentrations only when chelate cooperativity is sufficiently high, which can be achieved by increasing either EM, *K*, or both. Otherwise, the cyclic assemblies are in equilibrium with nonsorted linear oligomers and, at low concentrations, with the unbound monomers. Figure S1B shows the complete distribution of supramolecular species at several *K*–EM combinations for this hypothetical monomer mixture.

While NMR experiments already provided a reasonably clear picture of the self-assembly of mixtures of mono- and dinucleosides, we complemented these studies with CD and fluorescence spectroscopy experiments using the molecules labeled with **d**, **a**_**1**_, and **a**_**2**_ FRET dyes. The manifestation of narcissistic self-sorting in these mixtures of π-functional dinucleosides would allow us to organize each of them into independent assemblies with independent absorption and emission features. Otherwise, whenever donor and acceptor molecules are mixed in the same cyclic assembly, energy transfer processes would be triggered that would primarily result in donor emission quenching. We followed the same rationale as in the NMR experiments: the spectroscopic features of mononucleoside complementary pairs or of dinucleosides were examined first, and then, the relevant mixtures were generated and investigated. In contrast to the NMR results, some self-sorting tendency was observed in 1:1:1:1 mixtures of two orthogonal complementary nucleobase pairs bearing donor and acceptor FRET functions, like **dG** + **dC** + **a**_**1**_**A** + **a**_**1**_**U** (Figure S3A), in comparison with mixtures that have a single complementary pair, such as **dG** + **dC** + **a**_**1**_**G** + **a**_**1**_**C**, or mixtures with nonorthogonal pairs, like **d*****i*****G** + **d*****i*****C** + **a**_**1**_**G** + **a**_**1**_**C** in which donor:acceptor complexes can be formed that result in donor emission quenching due to FRET to the acceptor counterpart.

**Figure 4:**
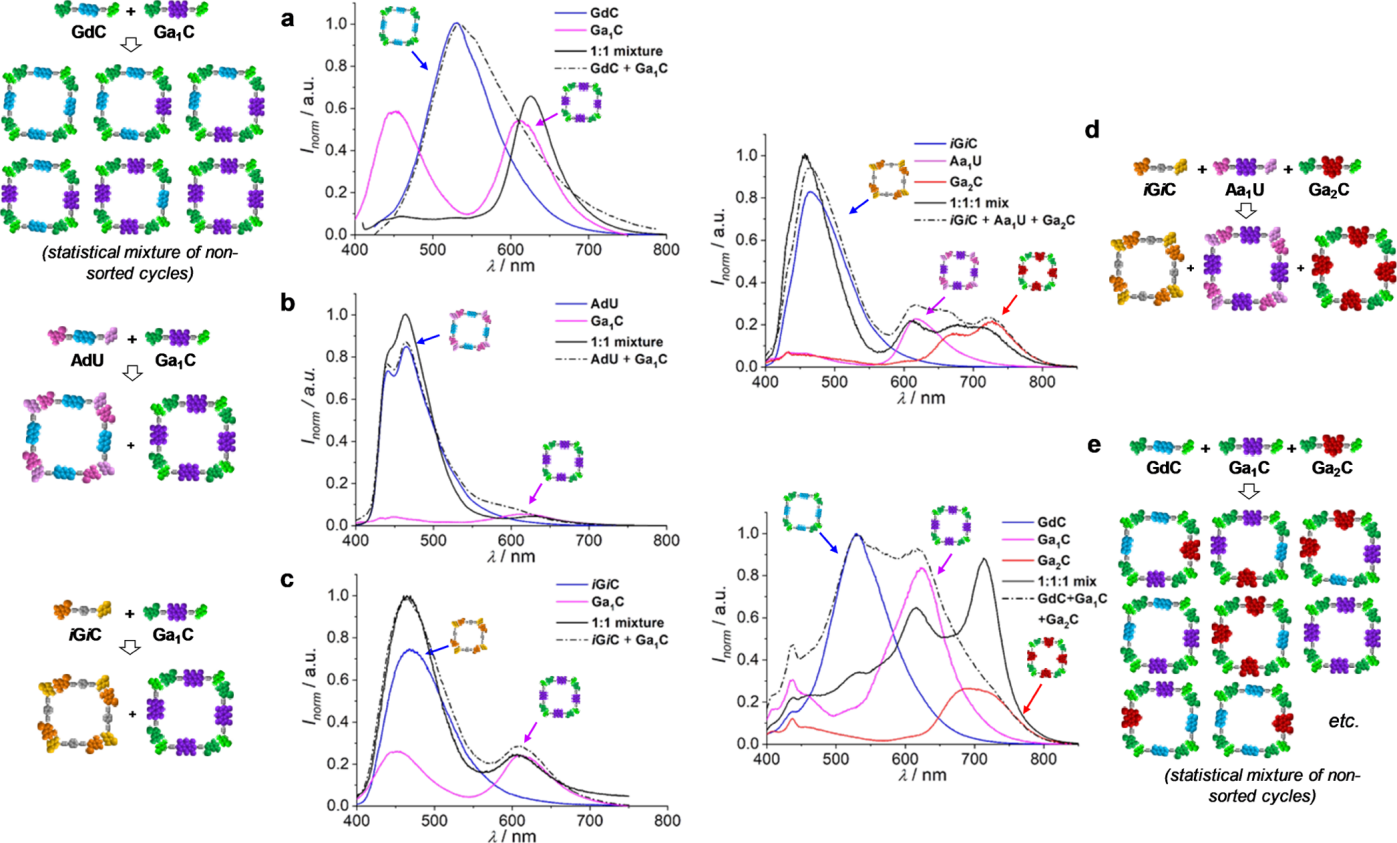
Emission spectra in toluene of (a) **GdC**, **Ga**_**1**_**C**, and their 1:1 mixture (λ_exc_ = 385 nm), (b) **AdU**, **Ga**_**1**_**C**, and their 1:1 mixture (λ_exc_ = 360 nm), (c) ***i*****G*****i*****C**, **Ga**_**1**_**C**, and their 1:1 mixture (λ_exc_ = 381 nm), (d) ***i*****G*****i*****C**, **Aa**_**1**_**U**, **Ga**_**2**_**C**, and their 1:1:1 mixture (λ_exc_ = 381 nm), or (e) **GdC**, **Ga**_**1**_**C**, **Ga**_**2**_**C**, and their 1:1:1 mixture (λ_exc_ = 386 nm). In all cases, the sum spectrum of the individual samples is shown with a dotted line so as to compare it with the experimental spectrum of the corresponding binary/ternary mixtures.

However, in agreement with the NMR results, self-sorting was greatly enhanced in the dinucleoside mixtures, independent of their H-bonding pattern (see Figures 4 and S3B–D). When donor and acceptor dinucleosides having the same complementary pairs were 1:1 mixed (like **GdC** + **Ga**_**1**_**C** or **AdU** + **Aa**_**1**_**U**; Figures 4a and S3B), mixed cyclic tetramers, some of them containing both donors and acceptors in close proximity, are formed, and a strong donor emission quenching is then recorded. In contrast, when donor and acceptor dinucleosides having orthogonal complementary pairs were 1:1 mixed (for instance, **GdC** + **Aa**_**1**_**U**, **AdU** + **Ga**_**1**_**C**, ***i*****Gd*****i*****C** + **Aa**_**1**_**U**, or **Aa**_**1**_**U** + **Ga**_**2**_**C**; Figures 4b and S3C), spectroscopic changes were virtually negligible with respect to the precursor solutions, indicating that FRET was not activated and thus that each molecule remained associated narcissistically in the corresponding cyclic tetramer. Even if the two pairs are not orthogonal (like ***i*****G*****i*****C** + **Ga**_**1**_**C**; Figures 4c and S3D) and the H-bonding pattern does not play any role, self-sorting is again ruled by the strong tendency of each monomer to cyclize narcissistically with strong cooperativities.

To push the system further, ternary mixtures containing the three Watson–crick complementary pairs and the three dyes were also generated (Figures 4d–e and S3E). As can be appreciated in Figure 4d, the emission spectrum of a ***i*****G*****i*****C** + **Aa**_**1**_**U** + **Ga**_**2**_**C** 1:1:1 mixture is basically the sum of the emission spectra of the three individual components, which supports strong narcissistic self-sorting. This is in sharp contrast with a control experiment in which **d**, **a**_**1**_, and **a**_**2**_ dyes were 1:1:1 mixed in dinucleosides with the same complementary base pair, namely, **GdC** + **Ga**_**1**_**C** + **Ga**_**2**_**C**. As shown in Figure 4e, this ternary blend exhibits substantial quenching of the **GdC** emission, weaker quenching of **Ga**_**1**_**C** emission, and significant emission enhancement of **Ga**_**2**_**C**, which indicates that a nonsorted mixture of all possible macrocycles, where donors and acceptors are combined in the same assembly and FRET is activated, is formed in solution.

**Figure 5:**
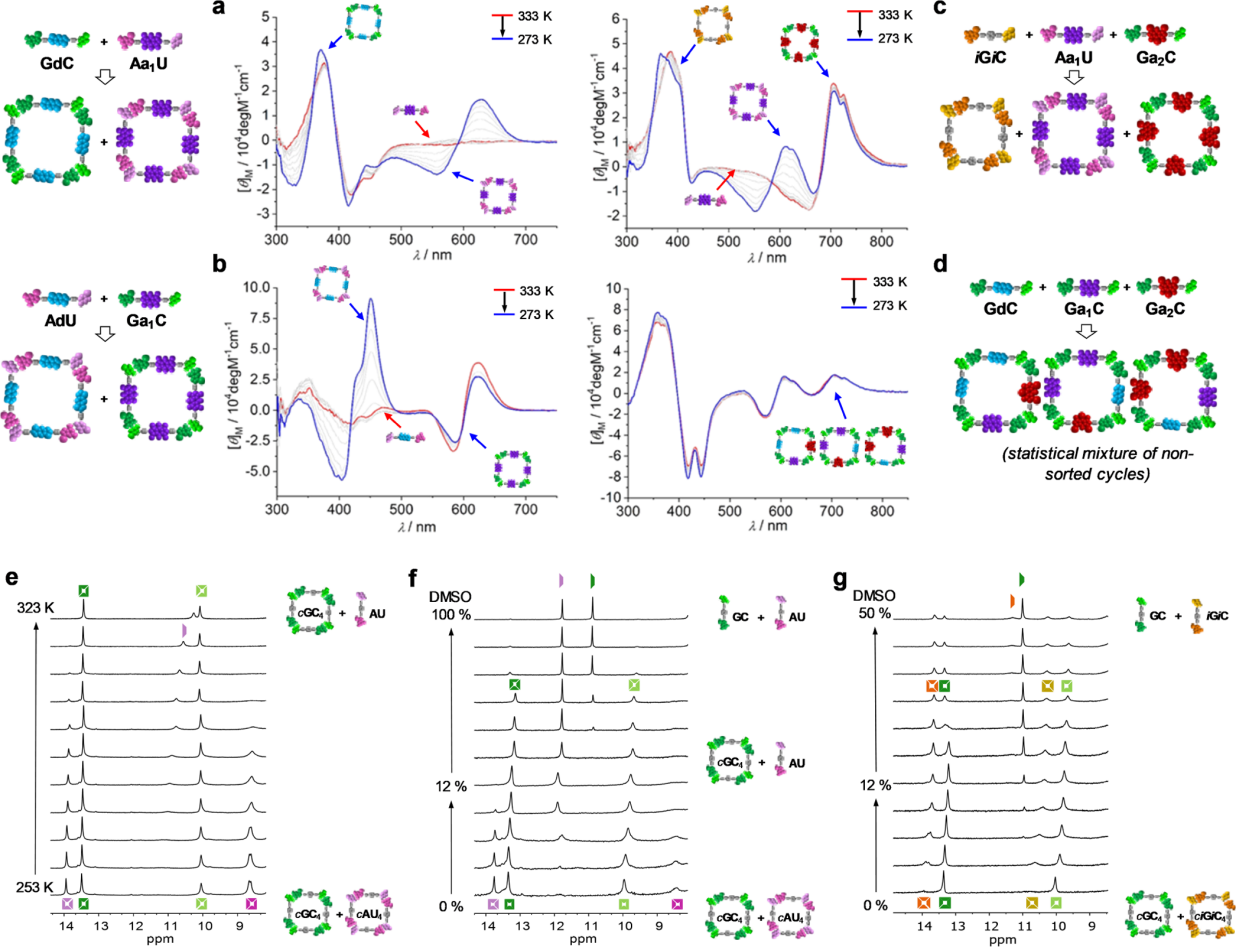
Selective cyclic tetramer dissociation. (a–d) Temperature-dependent CD spectra in toluene of (a) a 1:1 **GdC** + **Aa**_**1**_**U** mixture, (b) a 1:1 **AdU** + **Ga**_**1**_**C** mixture, (c) a 1:1:1 ***i*G*i*C** + **Aa**_**1**_**U** + **Ga**_**2**_**C** mixture, and (d) a 1:1:1 **GdC** + **Ga**_**1**_**C** + **Ga**_**2**_**C** mixture. (e, f) Downfield region of the ^1^H NMR spectra of a 1:1 mixture of **GC** + **AU** in (e) CDCl_3_ with increasing temperature or (f) CDCl_3_:CCl_4_ (2:3) with increasing DMSO-D_6_ content. (g) Downfield region of the ^1^H NMR spectra of a 1:1 mixture of **GC** + ***i*****G*****i*****C** in CDCl_3_ with increasing DMSO-D_6_ content. In the last mixture, the ^1^H signals of the *c*(***i*****G*****i*****C**)_4_ species are initially broad due to strong aggregation in pure CDCl_3_. A small amount of DMSO needs to be added to achieve complete solubility. For proton NMR codes, see Figure 1.

Moreover, due to the different macrocycle stability, we could selectively dissociate the weaker *c*(**AU**)_4_ macrocycles in the presence of stronger *c*(**GC**)_4_ and *c*(***i*****G*****i*****C**)_4_ cycles.^45^ This can be monitored by NMR or CD as a function of temperature or solvent composition (see Figures 5 and S4A–C). As previously demonstrated in all of our published work so far with these dinucleosides endowed with chiral groups,^37−39,42,43,45,46^ a characteristic CD signal emerges upon cyclotetramerization. The conformational “freezing” of the monomer skeleton upon cyclization allows the ribose groups to interact and transfer their chiral information to the π-conjugated backbone. In this way, a Cotton effect appears upon cyclization that matches the NMR trends in the same conditions and that can be used to monitor and quantify cyclic tetramer formation in a complementary manner and within a more dilute concentration window, whereby monomers or other noncyclic oligomers are CD-inactive.

Thus, as an example, Figure 5a,b shows the temperature-dependent CD spectra of 1:1 mixtures of **GdC** + **Aa**_**1**_**U** and **AdU** + **Ga**_**1**_**C**, respectively, in toluene. That is, the bithiophene **d** and BODIPY **a1** functional blocks, absorbing in different regions of the UV–vis spectrum, are swapped here in the corresponding GC and AU dinucleosides. At high temperatures, the weaker *c*(AU)_4_ macrocycles are almost entirely dissociated, and the corresponding **Aa**_**1**_**U** and **AdU** monomers afford no CD signal. Only when the temperature is decreased, the signals attributed to *c*(**Aa**_**1**_**U**)_4_ (Figure 5a) or *c*(**AdU**)_4_ (Figure 5b) arise.

The stronger *c*(**GdC**)_4_ or *c*(**Ga**_**1**_**C**)_4_ macrocycles, on the contrary, remain associated at all temperatures in these conditions. We also analyzed the previous ***i*****G*****i*****C** + **Aa**_**1**_**U** + **Ga**_**2**_**C** combination in a similar way. Figure 5c shows the CD spectrum of this ternary mixture, revealing the CD signatures of each self-sorted macrocycle in different spectral regions. When heating up to 333 K, only the CD signal of the weaker *c*(**Aa**_**1**_**U**)_4_ cycle, in the 450–650 nm region, was seen to vanish, as it progressively dissociates in the CD-silent **Aa**_**1**_**U** monomer. In contrast,^37^ the much stronger *c*(***i*****G*****i*****C**)_4_ and *c*(**Ga**_**2**_**C**)_4_ ensembles resisted the heating cycle without appreciable dissociation. Conversely, the control, nonsorted **GdC** + **Ga**_**1**_**C** + **Ga**_**2**_**C** blend revealed no CD change in these experiments (Figure 5d) because, as shown before, all G:C-bound macrocycles formed are sufficiently stable and do not dissociate under these conditions. A related example, now monitored by ^1^H NMR, is shown in Figure 5e, where we increased the temperature of a 1:1 **GC** + **AU** mixture in CDCl_3_. At high temperatures, only the weaker *c*(**AU**)_4_ cycle is dissociated, which is evidenced by the disappearance of the H-bonded U-imide proton signal at about 14 ppm and the concomitant appearance of a signal just below 11 ppm, attributed to the same proton in a mixture of monomer and small open oligomer species in fast equilibrium. This result is in line with previous observations monitored by CD. Similar results were obtained by increasing the volume fraction of DMSO-D_6_ in (2:3) CDCl_3_/CCl_4_ solutions of 1:1 **GC** + **AU** mixtures (Figure 5f), which led to the observation of two clear regimes. In the first one, from 0 to 12% v/v of DMSO-D_6_, *c*(**AU**)_4_ is progressively dissociated in the presence of the stronger *c*(**GC**)_4_ macrocycle, which show no sign of denaturation yet. This is evidenced by the appearance of the **AU** monomer U-imide signal at *ca.* 11.8 ppm. In the second regime, starting over *ca.* 20% DMSO-D_6_, *c*(**GC**)_4_ is then dissociated to the monomeric species, showing a G-amide signal at 10.9 ppm. It should be remarked, as noted in our previous work,^37,45^ that cyclic tetramers are always in slow exchange in the NMR timescale with their respective monomeric/oligomeric species, which highlights the strong cooperativity of the cyclotetramerization processes. When performing the same experiment with the **GC** + ***i*****G*****i*****C** mixture (Figure 5g), having similar *K*_a_ and EM values,^45^ cyclic tetramer dissociation occurs in parallel, and both **GC** and ***i*****G*****i*****C** monomers are detected in slow exchange at *ca.* 10.8 ppm after a 80% DMSO-D_6_ was added (Figure S4A).

In short, all of these results clearly demonstrate that self-sorting of cyclic assemblies is ruled *mainly* (for the G–C + A–U pair) or *exclusively* (for the G–C + iG–iC combination) by chelate cooperativity.

### Self-Sorting in Mixtures of Di- and Mononucleosides Sharing the Same Watson–Crick Interaction

Our next challenge then consisted in making the same intermolecular and intramolecular interaction to compete. In other words, we examined if self-sorting occurred in a mixture of mononucleosides and dinucleosides that share the same Watson–Crick H-bonding interaction. We selected two systems of very different cooperativity:^45^*c*(AU)_4_ (EM_AU_ ∼ 10^–1^–10^–2^ M) and *c*(GC)_4_ (EM_GC_ ∼10^2^–10^3^ M) and combined them with 1:1 mixtures of the corresponding A + U and G + C mononucleosides.

**Figure 6:**
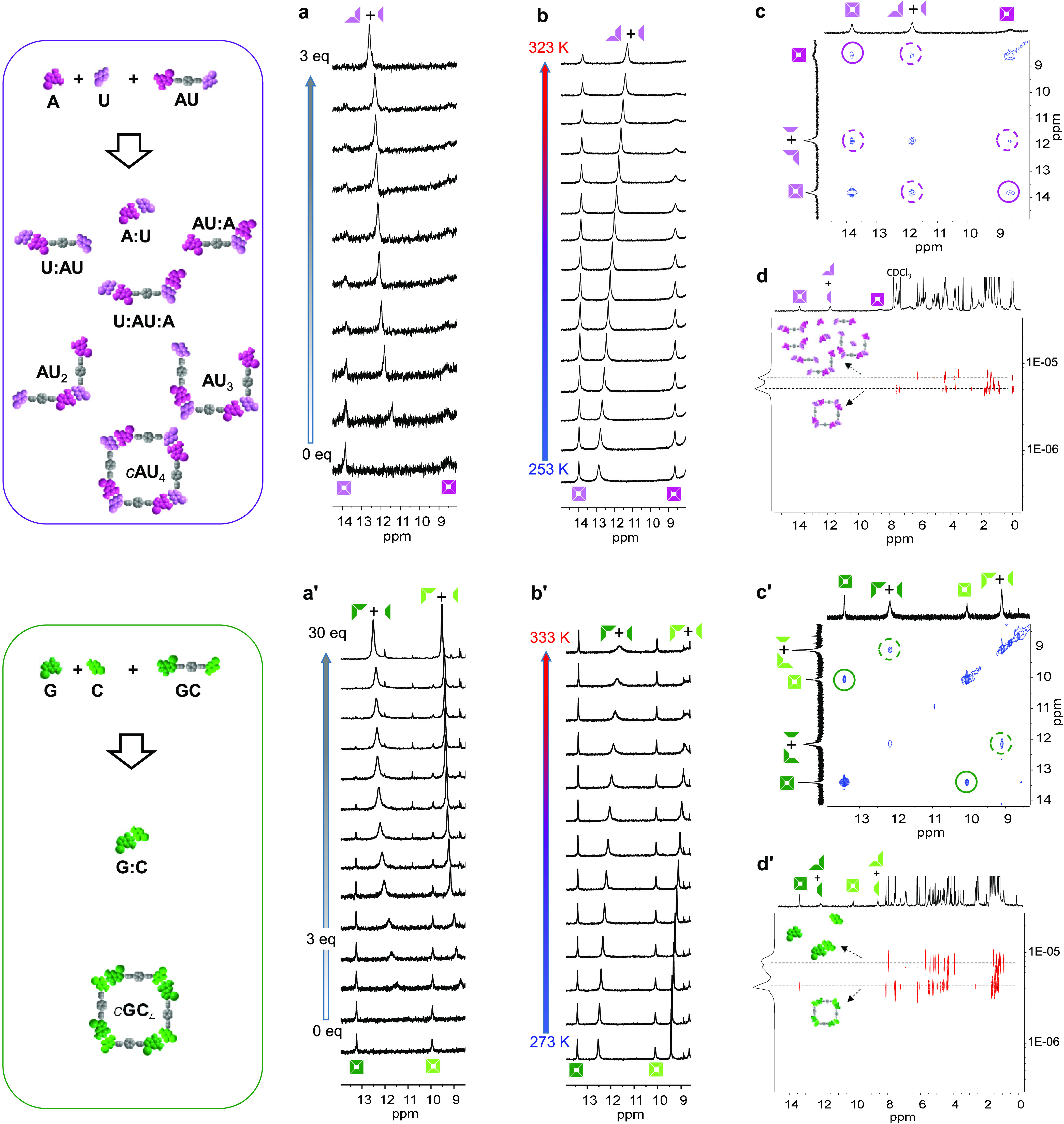
Analysis of ternary mixtures of dinucleoside and complementary mononucleosides. Top: **AU** + **A** + **U**. The *c*(**AU**)_4_ macrocycle having a low chelate cooperativity is expected to self-sort the mixture, to a small extent, leading to different associated species. Bottom: **GC** + **G** + **C**. On the contrary, the high cooperativity of the *c*(**GC**)_4_ macrocycle leads mainly to a narcissistically self-sorted mixture of *c*(**GC**)_4_ and the **G**:**C** complex. (a, a′) Titration experiments of a 1:1 mixture of mononucleosides onto a dinucleoside solution monitored in the 8–15 ppm region of the ^1^H NMR spectra, where the most relevant H-bonded proton signals are found: (a) **AU** + 1:1 **A** + **U** in CDCl_3_:CCl_4_ (2:3) and (a′) **GC** + 1:1 **G** + **C** in THF-D_8_. (b, b′) Evolution of the 8–15 ppm region of the ^1^H NMR spectra as a function of temperature for (b) a 1:1:1 mixture of **AU** + **A** + **U** and (b′) a 1:2:2 mixture of **GC** + **G** + **C**. (**c**, **c**′) NOESY spectra at *τ*_m_ = 500 ms and (d, d′) DOSY spectra for (c, d) a 1:1:1 mixture of **AU** + **A** + **U** in CDCl_3_:CCl_4_ (2:3) and (c′, d′) a 1:2:2 mixture of **GC** + **G** + **C** in THF-D_8_ (see Figure S5A–D for more details). For proton NMR codes, see Figure 1.

A first remarkable difference was seen in titration experiments of the dinucleoside, initially associated as cyclic tetramers, with the 1:1 mononucleoside mixture (Figures 6a,a′ and S5A). These experiments were conducted in a CDCl_3_:CCl_4_ (2:3) solvent mixture of **AU** and in THF-D_8_ for **GC**. These solvent systems were chosen so as to regulate the association constant (*K*) of the corresponding Watson–Crick pairs and maintain an adequate population of associated species within the concentration range studied.

**Figure 7:**
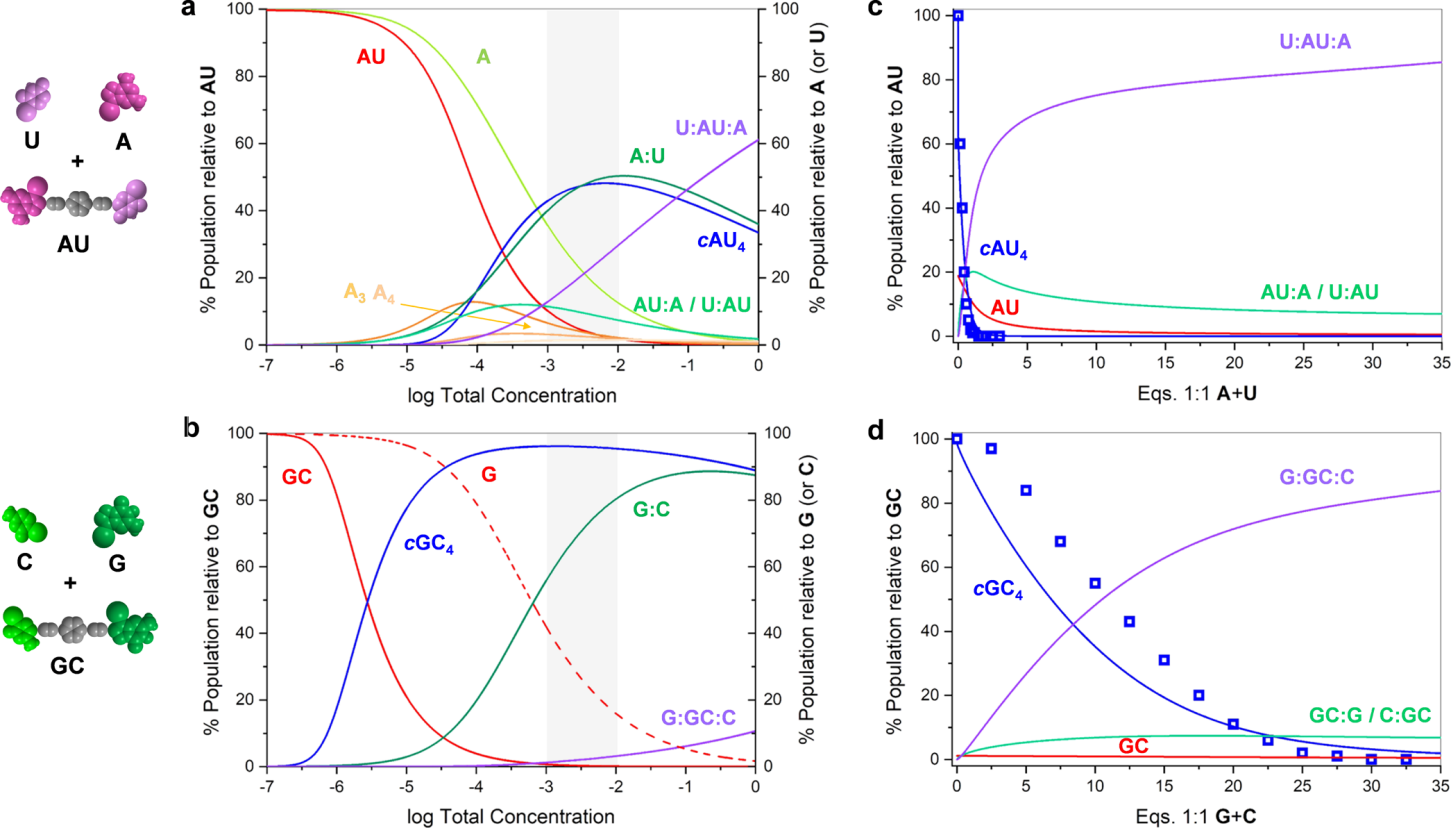
Simulation of ternary mixtures of dinucleoside and complementary mononucleosides. (a, b) Speciation curves showing the abundance of diverse species as a function of total concentration for 1:1:1 mixtures of (a) **AU** + **A** + **U** and (b) **GC** + **G** + **C**. (c, d) Distribution of species as a function of the amount of 1:1 mononucleoside mixture added: (c) **AU** + 1:1 **A** + **U** and (d) **GC** + 1:1 **G** + **C**. In both cases, the experimental titration data (squares; see Figure 6a,a′), obtained by ^1^H NMR signal integration, has been overlapped for comparison. Simulations were obtained using reported association constants and effective molarities (see Section S1).

Upon addition of a few equivalents (*i.e.*, <3 equiv.) of the competing 1:1 A + U mixture, the *c*(**AU**)_4_ cycle, which is in slow exchange at the NMR timescale, was rapidly dissociated by formation of additional Watson–Crick pairs between the added **A** + **U** mononucleosides and the terminal bases in the dinucleoside, leading to **U**:**AU**, **AU**:**A**, or **U**:**AU**:**A** associated species (see Figures 6a and 7c), which are in fast NMR exchange with other short oligomers, the **A**:**U** pair, and dissociated A and U. This was not the case of the more robust *c*(**GC**)_4_ macrocycle for which the intensity of its ^1^H NMR signals was not measurably reduced after the addition of a few equivalents of **G** + **C** (see Figures 6a′ and 7d). This means that the intra- and intermolecular versions of the G:C Watson–Crick pair can coexist self-sorted in solution without much interference, as long as the relative amount of mononucleosides is not high. After the addition of a high excess of G + C (above *ca*. 25 equiv; Figure 7d), the *c*(**GC**)_4_ species fully vanishes.

Self-sorting of *c*(**AU**)_4_/*c*(**GC**)_4_ and **A**:**U**/**G**:**C** complexes can also be evaluated in concentration- or temperature-dependent experiments (Figures 6b,b′ and S5B). For instance, due to the stronger cooperativity of *c*(**GC**)_4_, we can selectively dissociate the intermolecular G:C interaction by increasing the temperature in THF-D_8_ without affecting the population of *c*(**GC**)_4_ macrocycles. As shown in Figure 6b′, the signals attributed to the G-amide and C-amine in the mononucleosides shift upfield with temperature, due to a lower involvement in H-bonding, whereas the same signals in the dinucleoside remain unaltered in size, shape, and relative intensity, since *c*(**GC**)_4_ is not appreciably dissociated in these conditions. A similar behavior was noted for the **AU** + **A** + **U** combination, but in this case the relative amount of *c*(**AU**)_4_ does decrease with increasing temperature (see Figure S5B) due to the lower resistance of this weaker cycle.

A strong chelate cooperativity not only dominates self-sorting from a thermodynamic point of view but also the exchange kinetics of the dinucleoside molecule in the cyclic tetramer or in the mixture of oligomers is different for *c*(**AU**)_4_ and *c*(**GC**)_4_. Figure S5C displays the NOESY NMR spectra of **AU** + **A** + **U** and **GC** + **G** + **C** mixtures taken at different mixing times (τ_*m*_). At sufficiently long mixing times (like *τ*_*m*_ = 500 ms, as shown in Figure 6c), the exchange cross-peaks between **AU** in *c*(**AU**)_4_ in the fast-exchanging mixture of species are observed, and an exchange rate constant could be calculated as *k* = 1.8 s^–1^. In the **GC** + **G** + **C** mixture (Figure 6c’), no exchange cross-peaks were detected even at the longest mixing times or with higher amounts of competing **G** + **C** mononucleosides, which highlights the kinetic stability of the self-sorted *c*(**GC**)_4_ + **G**:**C** mixture. Furthermore, DOSY NMR experiments, as shown in Figures 6d,d′ and S5D, clearly revealed different diffusion coefficients for the two sets of species in slow exchange: (1) the larger *c*(**AU**)_4_ and *c*(**GC**)_4_ macrocycles and (2) the mixture of fast-exchanging oligomers (for **AU**) or the **G**:**C** pair (for **GC**).

Using the reference *K* and EM values, speciation curves could be generated for each of these **AU** + **A** + **U** and **GC** + **G** + **C** mixtures (Figure 7a,b) that simulate reasonably well our experimental results and provide a quantitative insight into the degree of self-sorting in these mixtures sharing the same interaction. As can be deduced from these curves, a high chelate cooperativity is demanded to achieve close to quantitative self-sorting in a wide range of concentrations. For instance, at the experimental 10^–3^–10^–2^ M NMR concentration (shadowed area in Figure 7a,b) in 1:1:1 **GC** + **G** + **C** mixtures, the molar fraction of **GC** dinucleoside molecules associated as *c*(**GC**)_4_ is well above 95% (Figure 7b). The main competitor for *c*(**GC**)_4_, especially at higher concentrations, is the trimolecular **C**:**GC**:**G** complex, and to a much lower extent, the **C**:**GC** and **GC**:**G** bimolecular complexes. On the other hand, within this concentration range in THF, the **G**:**C** complex accounts for *ca*. 80% of the total **G** concentration, the rest being dissociated **G** and the mentioned **C**:**GC**:**G** complex. If we now turn our attention to the 1:1:1 **AU** + **A** + **U** mixture at the experimental conditions employed, the *c*(**AU**)_4_ cycle abundance is only *ca*. 40–50%, and the participation of **U**:**AU** (or **AU**:**A**) bimolecular and **U**:**AU**:**A** trimolecular complexes is notable (i.e., each of them >10%; Figure 7a). This evidences a far lower degree of self-sorting when chelate cooperativity is not so powerful. A more detailed analysis of the dependence of self-sorting on chelate cooperativity, represented by the product *K·*EM, as a function of total concentration, can be found in Figure S1C.

Finally, Figure 7c,d shows how the distributions of **AU** and **GC** species, at an initial 10^–3^ M concentration, change as increasing amounts of, respectively, **A** + **U** and **G** + **C** mononucleosides are added, thus simulating the experiments displayed in Figure 6a,a′. Considering that mean *K* and EM values, obtained from previously published work, were used in these simulations, the agreement with the experimental data from the corresponding titrations (shown as colored squares) is reasonable. In both cases, the cyclic tetramer population decreases at the expense of **U**:**AU**, **AU**:**A**, **U**:**AU**:**A**/**C**:**GC**, **GC**:**G**, and **C**:**GC**:**G** species, but the *c*(**GC**)_4_ assembly, exhibiting a stronger cooperativity, can resist higher amounts of the mononucleoside mixture. Remarkably, self-sorting fidelity for *c*(**GC**)_4_ can be maintained as high as >95%, provided the amount of **G** + **C** added does not surpass *ca.* 5 equiv.

## Conclusions

In summary, a combination of one-dimensional (1D) and two-dimensional (2D) NMR, CD, and fluorescence spectroscopy with donor–acceptor FRET pairs, in diverse solvents and concentration ranges, clearly confirms the dominant role that chelate cooperativity can have in relatively complex mixtures. If the product(s) *K·*EM for a given (set of) cyclic species is (are) high enough, self-sorting (in this case, narcissistic self-sorting) can become quantitative. On one hand, we have proven this phenomenon in mixtures of dinucleoside molecules with identical geometry that are able to self-assemble into Watson–Crick H-bonded cyclic tetramers. It is here the strong propensity of (some of) the dinucleosides to form independently its own macrocycle, and not H-bonding complementarity, which drives narcissistic self-sorting. On the other hand, we have demonstrated that cyclic and noncyclic species that are bound by the same noncovalent interaction, or, in other words, the intra- and intermolecular version of a noncovalent interaction, can independently coexist as long as the cyclic species enjoys a strong intramolecular cooperativity and their relative concentration is comparable.

This is certainly not the first case in which chelate cooperativity has a strong influence on the self-sorting distribution of a mixture of supramolecular cyclic species. However, this work does represent the first qualitative and quantitative study on the relevance of chelate cooperativity on self-sorting and provides the first examples, to the best of our knowledge, in which an exclusive dominance is clearly demonstrated. The quantitative analysis performed herein is of course designed for these specific supramolecular structures. Due to the monomer structure and the geometry of Watson–Crick pairs, the dinucleoside molecules studied herein arrange in rectangular assemblies with corners showing 90° associations. This results in the absence of the structural strain and is one of the reasons for the high chelate cooperativities attained upon cyclotetramerization. Any structural deviation from this geometry changes the supramolecular scenario completely. Still, qualitative conclusions from this work are certainly applicable to other systems that, due either to a different monomer structure or a different binding geometry, associate in macrocycles or prisms with diverse molecularities and thermodynamic stabilities. Hence, the tools employed and conclusions attained here are general for any related supramolecular system in which chelate cooperativity is present, and intra- and intermolecular interactions are made to compete.

## Supporting Information Available

Experimental procedures and compound characterization data, along with Figures S1–S5 (PDF)
