# Supplementary material for: Self-Sorting Governed by Chelate Cooperativity
Source: J Am Chem Soc. 2022 Mar 19;144(12):5450–60. doi: 10.1021/jacs.1c13295 (PMC8972263; doi:10.1021/jacs.1c13295)
Supplement: Supplementary file 1 — ja1c13295_si_001.pdf [file ja1c13295_si_001.pdf]

## SUPPORTING INFORMATION

# SELF-SORTING GOVERNED BY CHELATE COOPERATIVITY

David Serrano-Molina,[ǂ] Carlos Montoro-García,[ǂ] María J. Mayoral,[ǂ,‡] Alberto de Juan,[ǂ] and David González-Rodríguez[ǂ,†]*

[ǂ]Nanostructured Molecular Systems and Materials Group, Departamento de Química Orgánica, Facultad de Ciencias, Universidad Autónoma de Madrid, 28049 Madrid, Spain.

[‡]Departamento de Química Inorgánica, Facultad de Ciencias Químicas, Universidad Complutense de Madrid, 28040 Madrid, Spain.

[†]Institute for Advanced Research in Chemical Sciences (IAdChem), Universidad Autónoma de Madrid, 28049 Madrid, Spain

## SUPPORTING INFORMATION

## TABLE OF CONTENTS

**S0. Synthesis and Characterization** ........ 2

**S0.1. New synthetic procedures and characterization data** ........ 4

**S0.2. $^{1}$H NMR, $^{13}$C NMR and MS spectra of the new compounds** ........ 9

**S1. Building Speciation Profiles** ........ 15

**S2. $^{1}$H NMR and NOESY Spectroscopy Measurements** ........ 20

**S2.1. $^{1}$H NMR and NOESY Spectroscopy Measurements of Mononucleoside Mixtures** ........ 20

**S2.2. $^{1}$H NMR and NOESY Spectroscopy Measurements of Dinucleoside Mixtures** ........ 22

**S3. CD and Emission Spectroscopy Measurements** ........ 26

**S3.1. CD and Emission Spectroscopy Measurements of Mononucleoside Mixtures** ........ 26

**S3.2. CD and Emission Spectroscopy Measurements of Dinucleoside Mixtures** ........ 28

**S4. Selective Dissociation Studies** ........ 31

**S5. Self-sorting in mixtures of mono- and dinucleosides** ........ 34

## S0. Synthesis and Characterization

**General Methods. ESI-HRMS** spectra were obtained from an *Applied Biosystems QSTAR* equipment, **PCI-HRMS** on a *Bruker MAXIS II* spectrometer, and **MALDI-TOF HRMS** on a Bruker Reflex III spectrometer. **NMR** spectra were recorded with a *BRUKER AVANCE-II* (300 MHz) instrument and *BRUKER DRX* 500 MHz. The temperature was actively controlled at 298 K. Chemical shifts are measured in ppm using the signals of the deuterated solvent as the internal standard [$CDCl_3$ calibrated at 7.26 ppm ($^1$H) and 77.0 ppm ($^{13}$C), DMSO-$D_6$ calibrated at 2.50 ppm ($^1$H) and 39.5 ppm ($^{13}$C) and THF-$D_8$ calibrated at 3.58 ($^1$H)]. **Column chromatography** was carried out on silica gel *Merck-60* (230-400 mesh, 60 Å), and TLC on aluminium sheets precoated with silica gel 60 F254 (Merck). **CD** and **UV-Visible** spectra were recorded with a *JASCO* V-815 equipment (measurement Information: data interval = 1 nm, data pitch = 1 nm, sensitivity = standard, D.I.T. = 1 sec, slit width = 1000 um). **Emission** spectra were recorded in a JASCO Fp-8600 equipment. Quartz cuvettes (1 and 0.1 cm path length) were used for the measurements. In these three instruments, the temperature was controlled using a *JASCO* Peltier thermostatted cell holder with a range of 263–383 K, adjustable temperature slope, and accuracy of ± 0.1 K. **Hyss** (Hyperquad Simulation and Speciation) program, developed by http://www.hyperquad.co.uk/index.htm, was used to generate the speciation plots.

**Starting materials and synthetic precursors.** Chemicals were purchased from commercial suppliers and used without further purification. Solid hygroscopic reagents were dried in a vacuum oven before use. Reaction solvents were thoroughly dried before use using standard methods. The syntheses of the great majority of the mononucleosides (Figure S0A) and dinucleosides (Figure S0B) employed herein, as well as their precursors, like **d-Br**, have already been reported by us.[1,2,3,4,5] Mononucleosides **G**, **C**, ***i*G**, ***i*C**, **A**, **U** were reported in ref. 1, whereas mononucleosides **dG**, **dC**, **dA**, **dU**, **a$_1$G**, **a$_1$C**, **a$_1$A**, **a$_1$U** were reported in ref. 2. The synthesis and characterization of mononucleosides **d*i*G** and **d*i*C** is described herein (see below). For mononucleoside compound structure, see Figure S0A. Dinucleoside **GC** was reported in ref. 3, dinucleosides ***i*G*i*C** and **AU** were reported in ref.4, whereas dinucleosides **GdC**, **AdU**, **Ga$_1$C** and **Aa$_1$U** were reported in ref.5. The synthesis and characterization of dinucleosides and ***i*Gd*i*C** and **Ga$_2$C** is described herein (see below). For dinucleoside compound structure, see Figure S0B. Compound **Ia$_1$I**[6,7] has been prepared following a procedure reported in the bibliography. **Ia$_2$I**[8,9] has been prepared employing a modified procedure from the one reported, as detailed below. In those cases where these intermediate compounds are known, their identity was checked exclusively by $^1$H NMR. **Br-d-Br** was purchased from commercial suppliers.

---

[1] Camacho-García, J.; Montoro-García, C.; López-Pérez, A. M.; Bilbao, N.; Romero-Pérez, S.; González-Rodríguez D. *Org. Biomol. Chem*. **2015**, *13*, 4506–4513.
[2] Mayoral, M. J.; Camacho-García, J.; Magdalena-Estirado, E.; Blanco-Lomas, M.; Fadaei, E.; Montoro-García, C.; Serrano-Molina, D.; González-Rodríguez, D. *Org. Biomol. Chem.* **2017**, *15*, 7558–7565.
[3] Monotoro-García, C.; Camacho-García, J.; López-Pérez, A. M.; Bilbao, N.; Romero-Pérez, S.; Mayoral, M. J.; González-Rodríguez, D. *Angew. Chem. Int. Ed.* **2015**, *54*, 6780–6784.
[4] Montoro-García, C.; Camacho-García, J.; López-Pérez, A. M.; Mayoral, M. J.; Bilbao, N.; González-Rodríguez, D. *Angew. Chem. Int. Ed.* **2016**, *55*, 223–227.
[5] Mayoral, M. J.; Serrano Molina, D.; Camacho-García, J.; Magdalena-Estirado, E.; Blanco-Lomas, M.; Fadaei, E.; González-Rodríguez, D. *Chem. Sci.* **2018**, *9*, 7809–7821.
[6] Mao, M.; Wang, J.-B.; Xiao, Z.-F.; Dai S.-Y.; Song Q.-H., *Dyes & Pigments*, **2012**, *94*, 224–232.
[7] Wang, L.; Wang, J.-W.; Cui, A.-j.; Cai, X.-X.; Wan, Y.; Chen, Q.; He M.-Y.; Zhang, W. *RSC Adv.* **2013**, *3*, 9219–9222.
[8] Kusaka, S.; Sakamoto, R.; Kitagawa, Y.; Okumura, M.; Nishihara, H. *Chem. Asian. J.* **2013**, *8*, 723–727.
[9] Huang, L.; Zhao, J.; Guo, S.; Zhang, C.; Ma, J. *J. Org. Chem.* **2013**, *78*, 5627–5637.

## MONONUCLEOSIDES

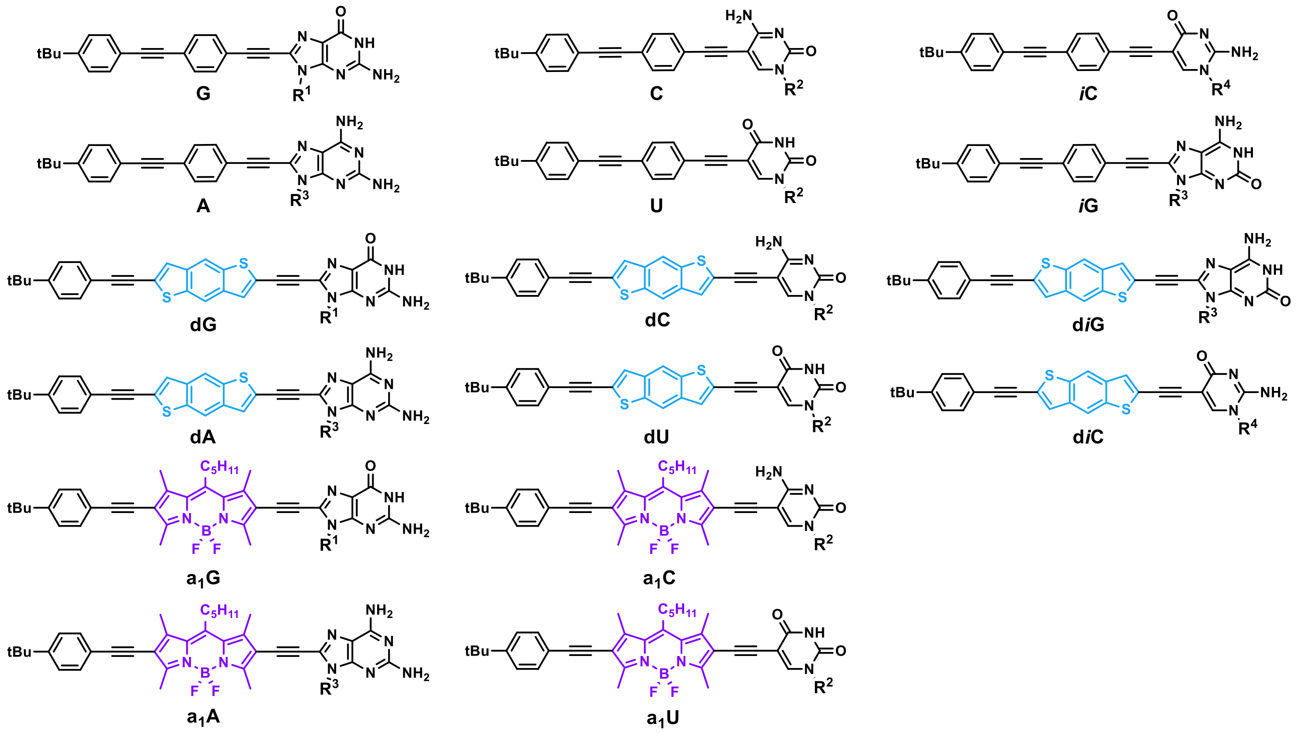


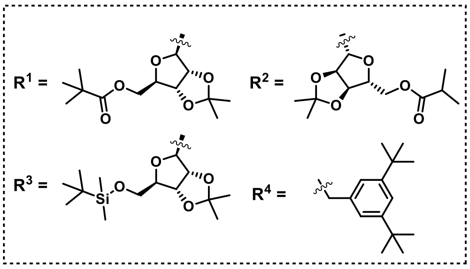


## DINUCLEOSIDES

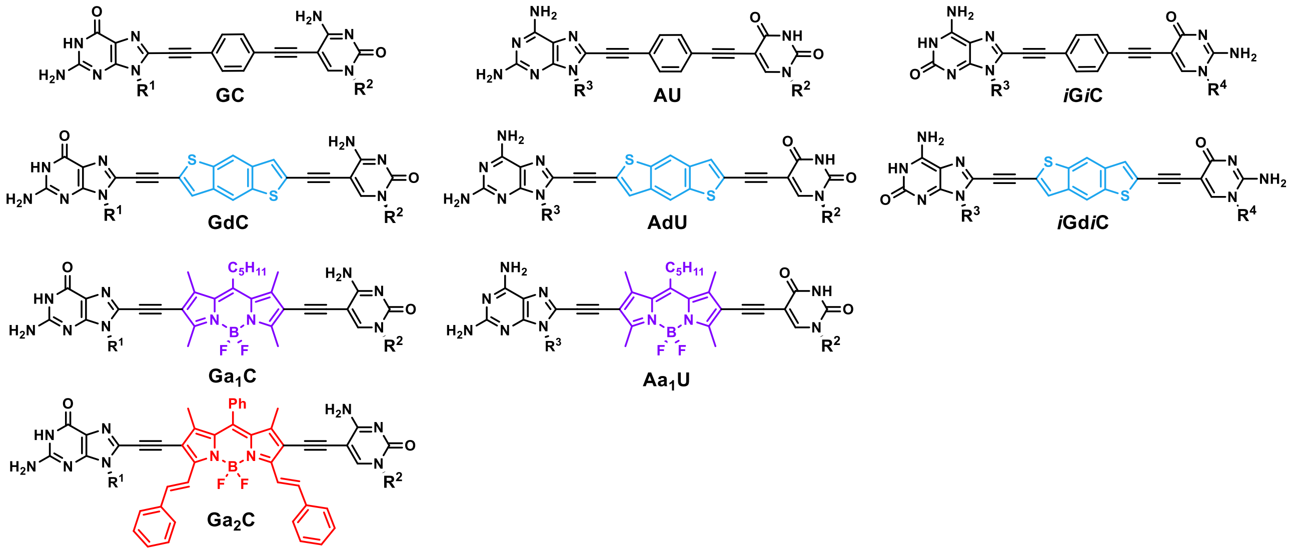


**Figure S0**. Structure of the dye-labelled and non-labelled mononucleosides and dinuclosides employed in this work.

## S0.1. New synthetic procedures and characterization data

### Synthesis of **I-a$_2$-I** (adapted from reported procedures)[8,9]

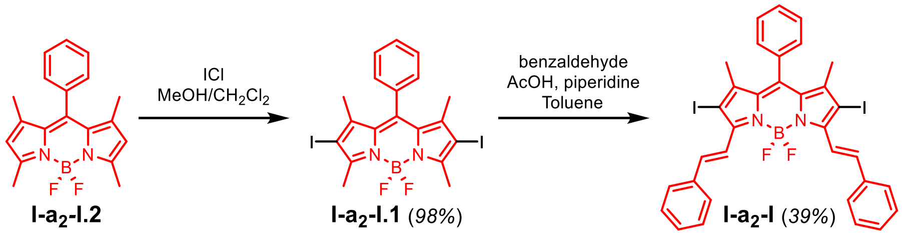


**Scheme S0A**. Synthetic route to **I-a$_2$-I**.

**I-a$_2$-I.1**. To a solution of **I-a$_2$-I.2**[10] (1 eq., 1.05 g, 3.24 mmol) in 240 mL of $CH_2Cl_2$/MeOH (1:1), ICl (3.4 eq., 1.79 mg, 11.01 mmol) dissolved in 20 mL of MeOH was added dropwise and stirred at room temperature for 1 h. The crude was then washed with aqueous $Na_2S_2O_5$(sat) (3x30 mL), water (3x30 mL) and brine (1x30 mL). **I-a$_2$-I.1** was obtained as a red solid in 98% yield. $^1$H NMR (300 MHz, $CDCl_3$) δ (ppm) = 7.47-7.40 (m, 3H), 7.21-7.13 (m, 2H), 2.57 (s, 6H), 1.31 (s, 6H).

**I-a$_2$-I**. **I-a$_2$-I.1** (1.0 eq., 500 mg, 0.87 mmol), benzaldehyde (4.0 eq., 0.35 mL, 3.47 mmol), piperidine (1.3 mL) and AcOH (1.0 mL) were refluxed in 75 mL of toluene using a Dean-Stark apparatus to remove the water formed during the reaction. After 5 h, the mixture was cooled down to room temperature and washed with aqueous $NaHCO_3$(sat) (3x10 mL) and water (3x10 mL). After column chromatography using $CHCl_3$ as eluent, **I-a$_2$-I** was obtained as a green solid in 39% yield. $^1$H NMR (300 MHz, $CDCl_3$) δ (ppm) = 8.15 (d, $J$ = 17.3 Hz, 2H), 7.67 (d, $J$ = 7.9 Hz, 5H), 7.58-7.51 (m, 3H), 7.46-7.27 (m, 9H), 1.46 (s, 6H).

### General procedure for the Sonogashira cross-coupling reaction

A dry THF, DMF, THF/$NEt_3$ or DMF/$NEt_3$ (4:1) mixture was subjected to deoxygenation by three freeze-pump-thaw cycles with argon. It was then poured over a round-bottom flask containing the corresponding amount of the compound bearing the ethynyl group, the right proportion of halogenated species, $Pd(PPh_3)_2Cl_2$ (0.02 eq.) and CuI (0.01 eq.). The resulting mixture was stirred under argon atmosphere at the corresponding temperature for each case. Once completed, the mixture was filtrated over a celite plug and the solvent was evaporated under reduced pressure. The residue was purified by silica gel column chromatography using the respective eluent to give the desired products. Any slight modification of this procedure will be remarked in each case.

[10] Chen, Y.; Zhao, J.; Xie, L.; Guo, H.; Li, Q. *RSC Advance* **2012**, *2*, 2942–3953.

## Synthesis of d*i*G and d*i*C

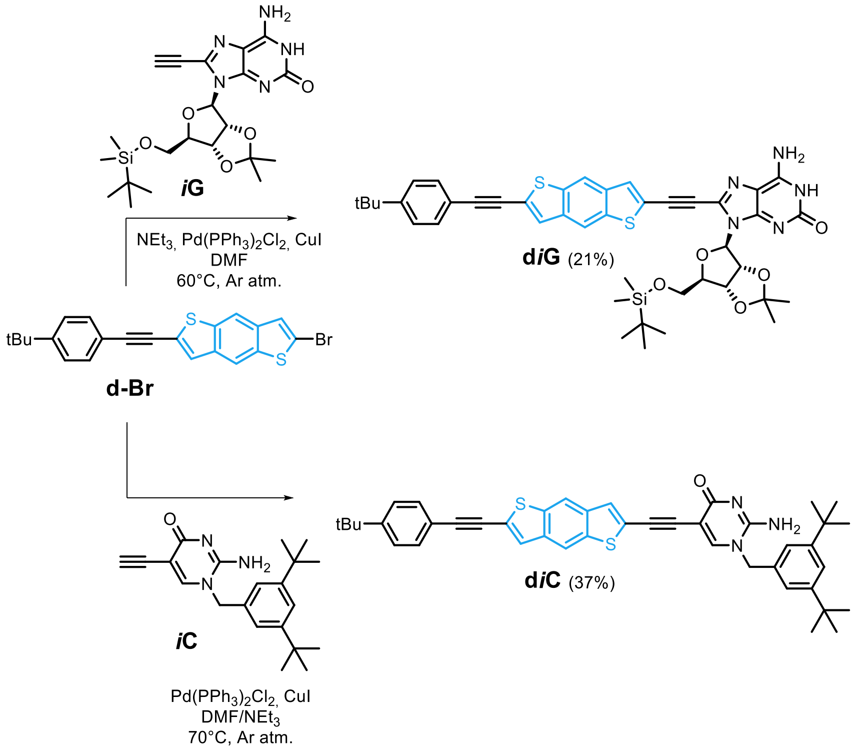

**Scheme S0B**. Synthesis of **d*i*G** and **d*i*C** from **d-Br** and the corresponding nucleobase (***i*G** / ***i*C**) through a Sonogashira cross-coupling reaction.

**d*i*G**. Following the general procedure for the Sonogashira cross-coupling reaction described above, this compound was prepared from **d-Br**[2] (1.0 eq., 31 mg, 0.071 mmol), ***i*G** (1.0 eq., 33 mg, 0.071 mmol), $NEt_3$ (1.2 eq., 12 µL, 0.085 mmol) and DMF as solvent (3 mL). The reaction mixture was stirred at 70ºC overnight. The crude was purified using $CHCl_3$/MeOH (30:1) as eluent. **d*i*G** was obtained as a yellow solid in 21% yield. $^1H$ NMR (300 MHz, DMSO-$D_6$) δ (ppm) = 10.76 (bs, 1H), 8.47 (s, 1H), 8.45 (s, 1H), 7.94 (s, 1H), 7.75 (s, 1H), 7.52 (d, $J$ = 6.8 Hz, 2H), 7.44 (d, $J$ = 7.2 Hz, 2H), 7.15 (bs, 2H), 6.04 (s, 1H), 5.62-5.53 (m, 1H), 5.02-4.93 (m, 1H), 4.17-4.06 (m, 1H), 3.86-3.65 (m, 2H), 1.56 (s, 3H), 1.35 (s, 3H), 1.29 (s, 9H), 0.77 (s, 9H), -0.08 (s, 6H). $^{13}C$ NMR (76 MHz, DMSO-$D_6$) δ (ppm) = 152.5, 138.5, 137.9, 137.8, 137.4, 131.4, 130.7, 128.2, 125.8, 124.5, 120.9, 118.9, 117.7, 117.2, 113.7, 96.3, 89.4, 87.7, 87.6, 84.9, 82.7, 82.6, 82.2, 63.8, 34.9, 31.3, 27.4, 26.0, 25.7, 18.3, -5.0, -5.1. HRMS (ESI+): Calculated for $C_{43}H_{47}N_5O_5S_2Si$: 806.2788 $[M]^+$. Found: 806.2891 $[M]^+$.

**d*i*C**. Following the general procedure for the Sonogashira cross-coupling reaction described above, this compound was prepared from **d-Br**[2] (1.0 eq., 40 mg, 0.093 mmol), ***i*C** (1.0 eq., 32 mg, 0.093 mmol) and a mixture of DMF/ $NEt_3$ (4:1) as solvent (3 mL). The reaction mixture was stirred at 60 ºC during 2 h. The crude was purified using $CHCl_3$/MeOH (30:1) as eluent. **d*i*C** was obtained as a brown solid in 37% yield. $^1H$ NMR (300 MHz, DMSO-$D_6$) δ (ppm) = 9.03 (bs, 1H), 8.59 (s, 1H), 8.58 (s, 1H), 8.31 (s, 1H) 8.15 (s, 1H), 7.82 (s, 1H), 7.60-7.53 (d and bs, $J$ = 7.4 Hz, 3H), 7.49 (d, $J$ = 8.49 Hz, 2H), 7.43 (s, 1H), 7.24 (s, 2H), 5.46 (s, 2H), 1.37-1.25 (m, 27H). $^{13}C$ NMR (125 MHz, DMSO-$D_6$) δ (ppm) = 169.5, 154.8, 152.9, 151.8, 151.6, 138.3,

137.9, 137.3, 132.3, 131.7, 130.6, 126.6, 124.1, 123.8, 123.9, 122.8, 122.6, 118.8, 118.5, 118.2, 113.5, 102.0, 99.9, 96.6, 82.7, 79.6, 57.0, 35.1,31.7, 31.6, 31.3, 29.6, 29.1, 23.5, 22.5, 19.6, 14.4, 13.9. HRMS (APCI+): Calculated for $C_{43}H_{43}N_3OS_2$: 682.2847 $[M+H]^+$. Found: 682.2923 $[M+H]^+$.

### Synthesis of Ga₂C and *i*Gd*i*C

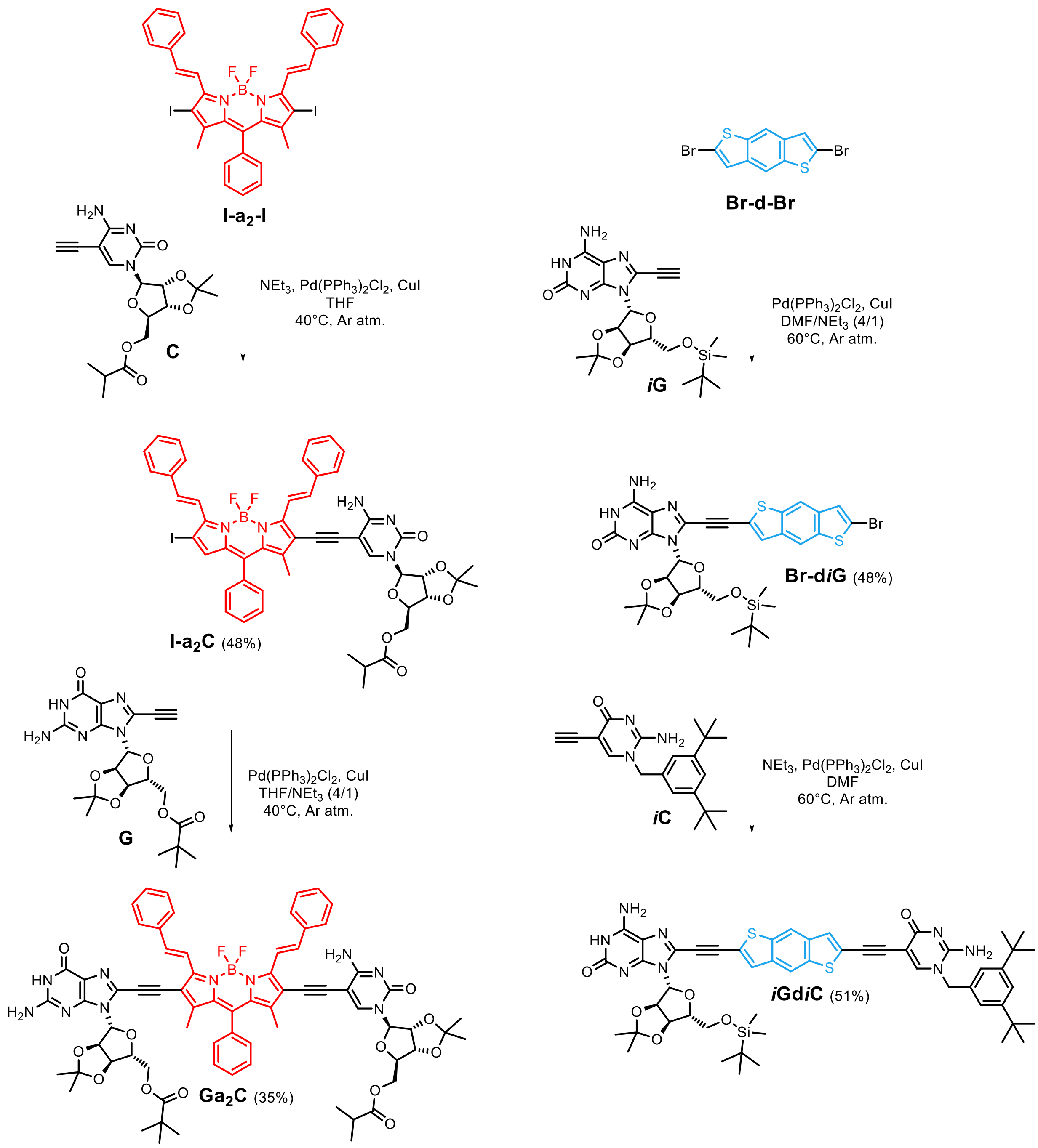


**Scheme S0C**. Synthesis of the dye-labelled **Ga2C** and ***i*Gd*i*C** dinucleosides *via* two consecutive Sonogashira reactions between the central block and the corresponding nucleobase derivatives.

**I-a₂C**. Following the general procedure for the Sonogashira cross-coupling reaction described above, this compound was prepared from **I-a₂-I** (2.0 eq., 100mg, 0.132 mmol), **C** (1.0 eq., 25 mg, 0.066 mmol), $NEt_3$ (2.4

eq, 22 µL, 0.158 mmol) and THF (5 mL) as solvent. The reaction mixture was stirred at 40°C overnight. The crude was purified using $CHCl_3$/MeOH (50:1) as eluent. **I-a$_2$C** was obtained as a dark blue solid in 48% yield. $^1$H NMR (300 MHz, $CDCl_3$/DMSO-*$D_6$*) δ (ppm) = 8.15 (d, *J* = 17.0 Hz, 2H), 7.78 (s, 1H), 7.72-7.12 (m, 19H), 6.02 (bs, 1H), 5.71 (s, 1H), 4.93 (d, *J* = 3.9 Hz, 1H), 4.74 (s, 1H), 4.37-4.14 (m, 3H), 2.16 (sep, *J* = 7.0 Hz, 1H), 1.47 (s, 3H), 1.43 (s, 3H), 1.23-1.13 (m, 6H), 1.06-0.96 (m, 6H). $^{13}$C NMR (76 MHz, DMSO-*$D_6$*) δ (ppm) = 182.7, 175.5, 145.5, 138.8, 135.6, 133.7, 132.6, 131.4, 131.4, 131.3, 131.2, 131.2, 129.2, 129.1, 128.4, 128.1, 127.9, 127.4, 127.0, 126.9, 112.8, 94.7, 85.0, 84.5, 80.6, 63.5, 33.0, 31.2, 28.9, 28.6, 26.6, 24.7, 21.9, 21.5, 18.3, 18.2, 16.8, 13.5, 12.8. HRMS (MALDI+): Calculated for $C_{51}H_{47}BF_2IN_5O_6$: 1001.2632 $[M]^+$. Found: 1001.2641 $[M]^+$.

**Ga$_2$C**. Following the general procedure for the Sonogashira cross-coupling reaction described above, this compound was prepared from **I-a$_2$C** (1.0 eq., 44 mg, 0.044 mmol), **G** (1.0 eq., 19 mg, 0.044 mmol) and a mixture of THF/$NEt_3$ (4/1) as solvent (3 mL). The reaction mixture was stirred at 40°C during 2.5 h. The crude was purified using $CHCl_3$/MeOH (20:1) as eluent. **Ga$_2$C** was obtained as a dark blue solid in 35% yield. $^1$H NMR (300 MHz, $CDCl_3$/DMSO-*$D_6$*) δ (ppm) = 10.85 (bs, 1H), 8.24 (d, *J* = 16.4 Hz, 1H), 8.23 (d, *J* = 16.3 Hz, 1H), 8.02 (s, 1H), 7.93 (bs, 1H), 7.73-7.27 (m, 17H), 7.06 (bs, 1H), 6.44 (bs, 2H), 6.11 (s, 1H,), 5.74 (s, 1H,), 5.30 (d, *J* = 6.6 Hz, 1H,), 5.21-5.14 (m, 1H,), 4.95 (d, *J* = 6.0 Hz, 1H,), 4.78-4.71 (dd, *J* = 6.2 and 3.4 Hz, 1H,), 4.41-3.98 (m, 6H), 2.43 (sept, *J* = 7.2 Hz, 1H), 1.49 (s, 3H) 1.47 (s, 3H), 1.31-1.16 (m, 18 H), 1.07 (s, 9H). $^{13}$C NMR (125 MHz, DMSO-*$D_6$*) δ (ppm) = 177.2, 175.5, 156.3, 153.7, 151.5, 149.8, 146.1, 145.9, 140.4, 139.4, 138.9, 135.7, 135.6, 133.3, 132.3, 132.0, 129.6, 129.3, 129.2, 129.1, 128.6, 128.4, 127.4, 127.3, 127.2, 117.8, 113.1, 113.0, 99.9, 94.6, 89.3, 87.2, 86.9, 85.5, 85.1, 84.5, 83.6, 81.7, 80.7, 63.8, 63.6, 45.6, 39.5, 38.0, 33.0, 31.2, 28.9, 26.6, 26.4, 25.0, 22.0, 18.4, 18.3, 8.2. HRMS (ESI+): Calculated for $C_{71}H_{71}BF_2N_{10}O_{12}$ : 1305.5314 $[M+H]^+$. Found: 1305.5372 $[M+H]^+$.

**Br-d*i*G**. Following the general procedure for the Sonogashira cross-coupling reaction described above, this compound was prepared from **Br-d-Br** (3.0 eq., 97 mg, 0.605 mmol), ***i*G** (1.0 eq., 80 mg, 0.093 mmol), and a DMF/$NEt_3$ (4:1) mixture as solvent (5 mL). The reaction mixture was stirred at 60ºC overnight. The crude was purified using $CHCl_3$/MeOH (30:1) as eluent. **Br-d*i*G** was obtained as a yellow solid in 48% yield. $^1$H NMR (300 MHz, DMSO-*$D_6$*) δ (ppm) = 10.74 (bs, 1H, NH), 8.44 (s, 1H, *H*-f or *H*-k), 8.37 (s, 1H), 7.91 (s, 1H), 7.64 (s, 1H), 6.04 (s, 1H), 5.58 (d, *J* = 6.6 Hz, 1H), 4.97 (dd, *J* = 6.4 and 3.4 Hz, 1H), 4.15-4.07 (m, 1H), 3.85-3.65 (m, 2H), 1.55 (s, 3H), 1.34 (s, 3H), 0.77 (s, 9H), -0.09 (s, 6H). $^{13}$C NMR (76 MHz, DMSO-*$D_6$*) δ (ppm) = 149.4, 138.2, 138.0, 137.29, 136.8, 135.9, 132.8, 130.5, 126.0, 123.9, 122.1, 121.1, 120.0, 117.2, 115.9, 113.1, 96.6, 87.1, 82.2, 82.0, 81.3, 63.2, 26.9, 25.5, 25.1, 17.7, -5.5, -5.6. HRMS (ESI+): Calculated for $C_{31}H_{34}BrN_5O_5S_2Si$: 728.0954 $[M+H]^+$. Found: 728.1024 $[M+H]^+$.

***i*Cd*i*G**. Following the general procedure for the Sonogashira cross-coupling reaction described above, this compound was prepared from **Br-d*i*G** (1.0 eq., 31 mg, 0.042 mmol), ***i*C** (1.2 eq., 17 mg, 0.051 mmol), $NEt_3$ (1.2 eq, 7 µL, 0.051 mmol) and DMF as solvent. The reaction mixture was stirred at 60ºC during 2 h. The crude was purified using $CHCl_3$/MeOH (30:1) as eluent. ***i*Cd*i*G** was obtained as a brown solid in 51% yield.

$^{1}$H NMR (300 MHz, DMSO-$D_6$) δ (ppm) = 10.90 (bs, 1H), 8,44 (bs, 1H) 8.39 (s, 1H), 8.33 (s, 1H), 8.16 (s, 1H) 7.87 (s, 1H), 7.59 (s, 1H), 7.31-7.11 (m, 6H), 6.03 (s, 1H), 5.61-5.53 (m, 1H), 4.96 (s, 2H), 4.15-4.06 (m, 1H), 3.84-3.66 (m, 3H), 1.55 (s, 3H), 1.33 (s, 3H), 1.21 (s, 18H), 0.77 (s, 9H), -0.09 (s, 6H). $^{13}$C NMR (76 MHz, DMSO-$D_6$) δ (ppm) = 149.9, 138.1, 137.3, 135.8, 130.1, 127.6, 126.2, 125.7, 124.6, 121.2, 120.2, 116.9, 116.4, 115.6, 113.1, 99.5, 89.0, 88.9, 87.1, 84.0, 82.2, 82.1, 81.7, 63.3, 34.2, 31.2, 31.1, 28.9, 28.6, 25.47, 21.9, 17.8, 13.7, 13.2, -5.6, -5.7. HRMS (ESI+): Calculated for $C_{52}H_{60}N_8O_6S_2Si$: 985.3846 $[M+H]^+$. Found: 985.3905 $[M+H]^+$.

## S0.2. $^1$H NMR, $^{13}$C NMR and MS spectra of the new compounds

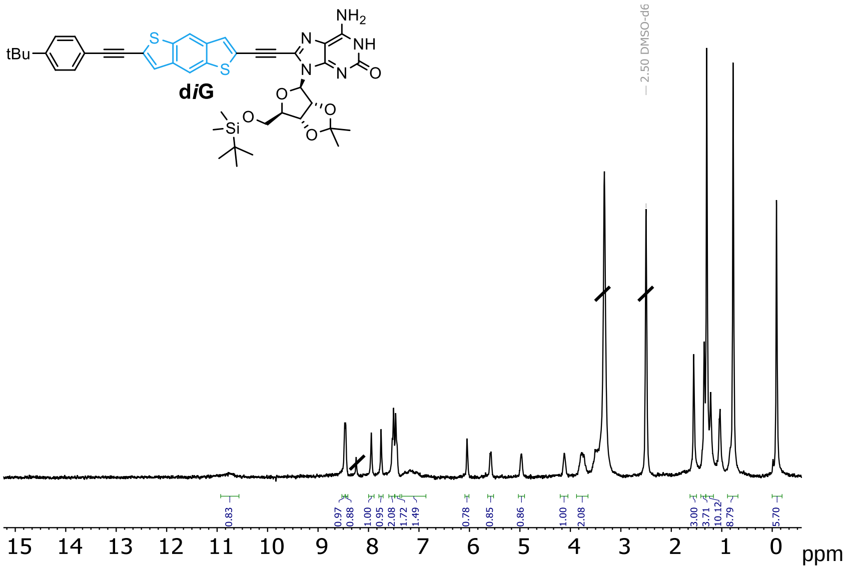


$^1$H-NMR spectrum of **d*i*G** (300 MHz, DMSO-$D_6$)

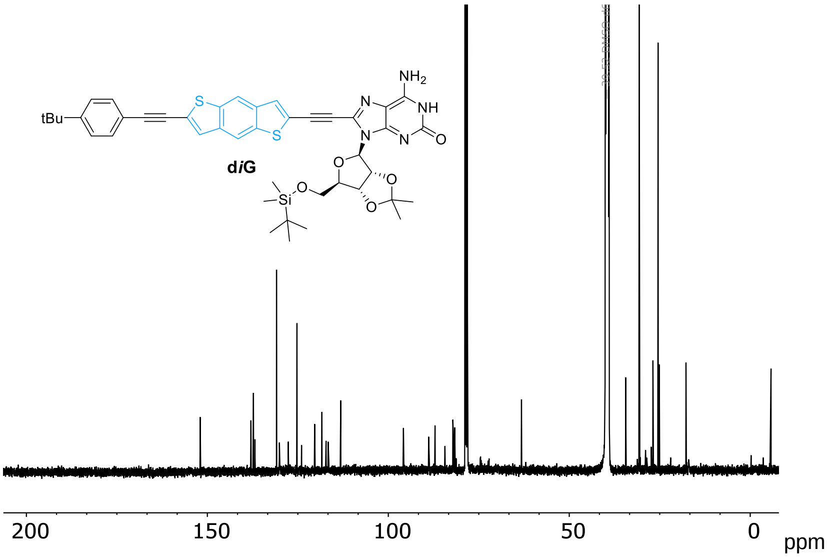


$^{13}$C-NMR spectrum of **d*i*G** (76 MHz, DMSO-$D_6$)

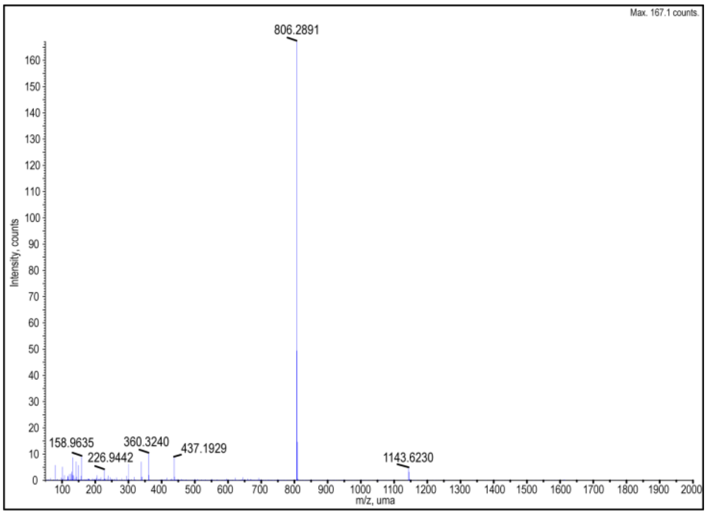


HRMS (ESI+) spectrum of **d*i*G**

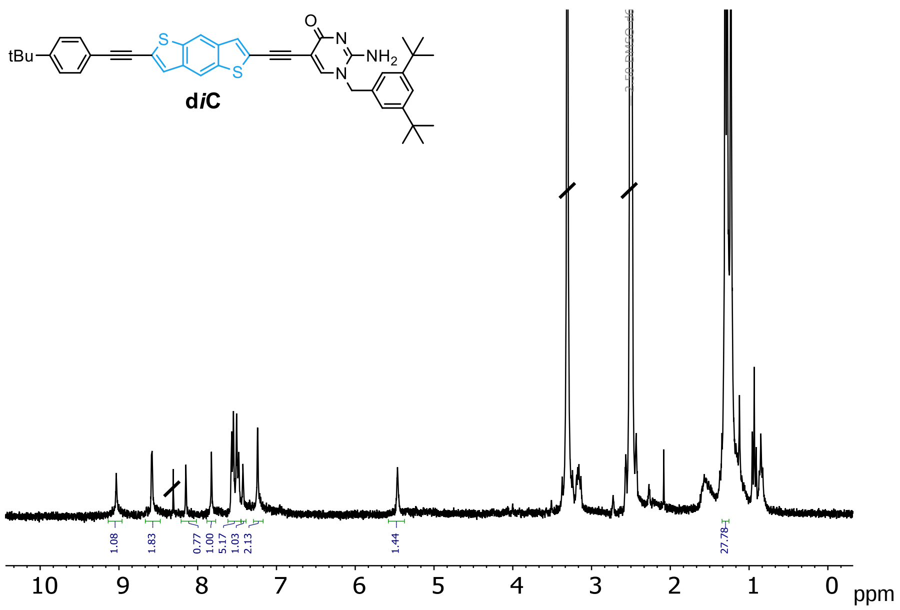


$^{1}$H-NMR spectrum of **d*i*C** (300 MHz, DMSO-$D_6$)

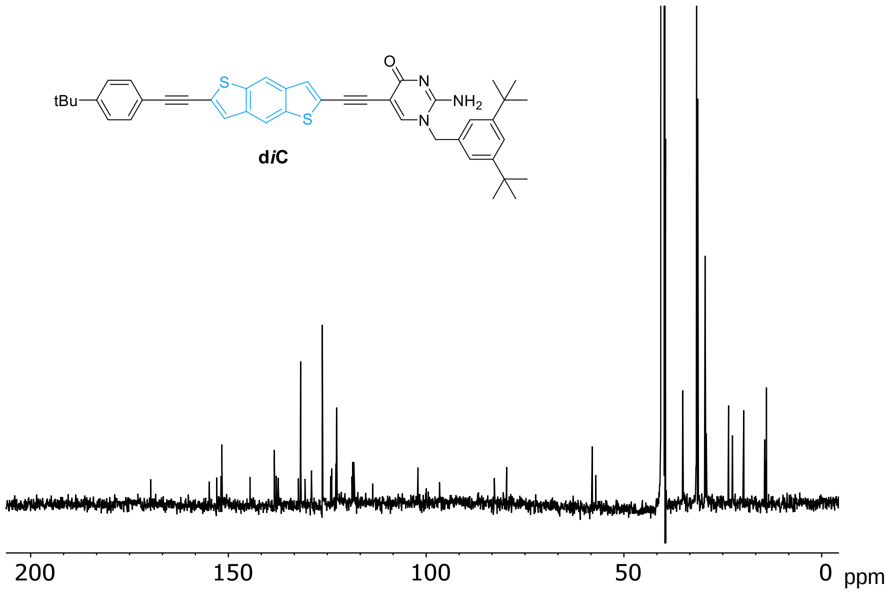


$^{13}$C-NMR spectrum of **d*i*C** (125 MHz, DMSO-$D_6$)

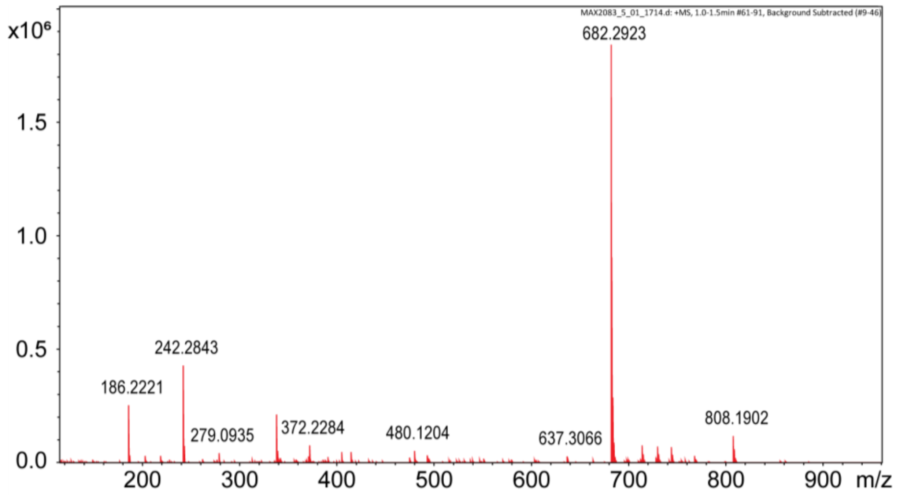


HRMS (APCI+) spectrum of **d*i*C**

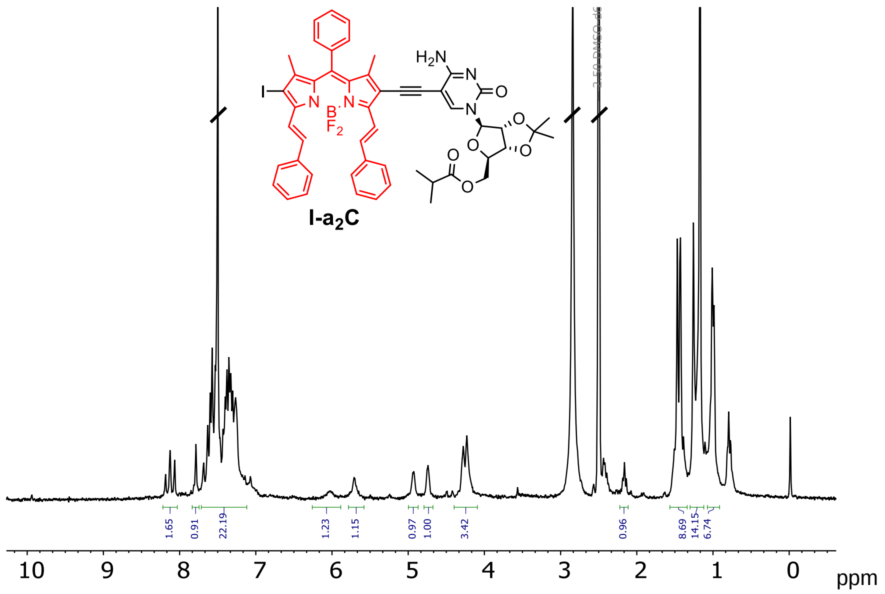


$^{1}$H-NMR spectrum of **I-a$_2$C** (300 MHz, $CDCl_3$/DMSO-*$D_6$*)

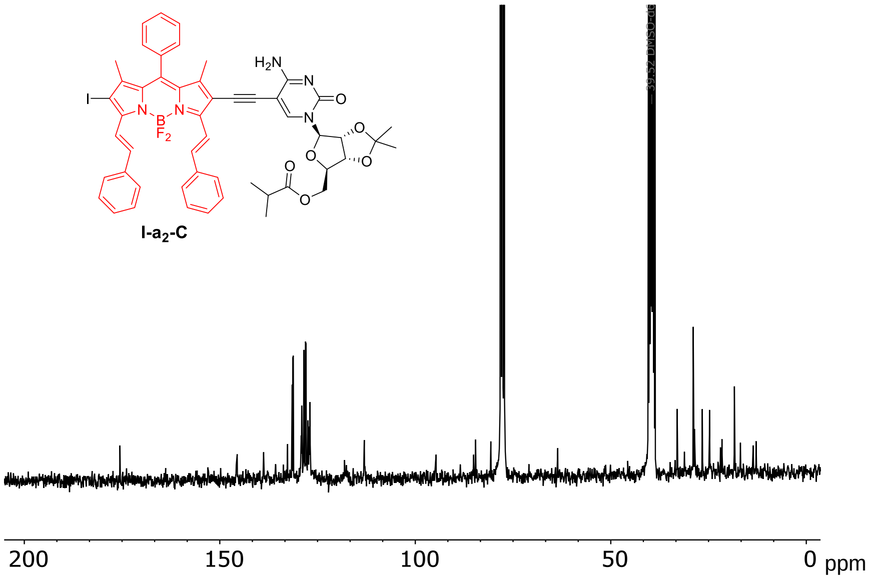


$^{13}$C-NMR spectrum of **I-a$_2$C** (76 MHz, DMSO-*$D_6$*)

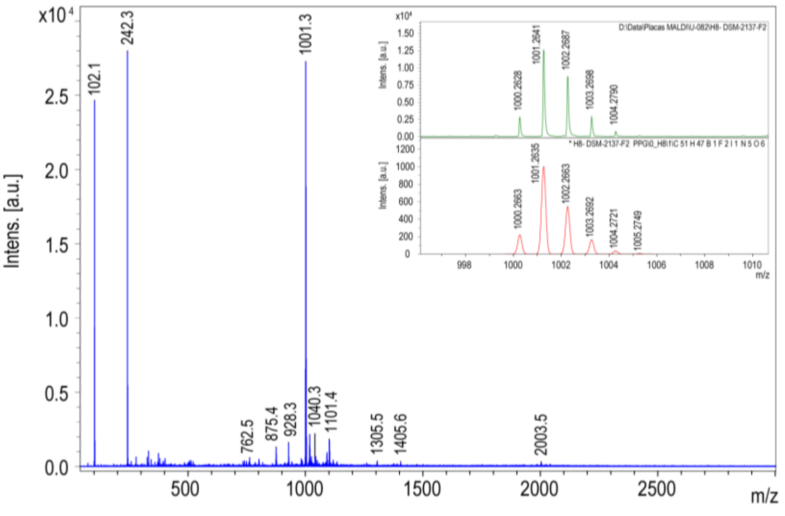


HRMS (MALDI+) spectrum of **I-a$_2$C**

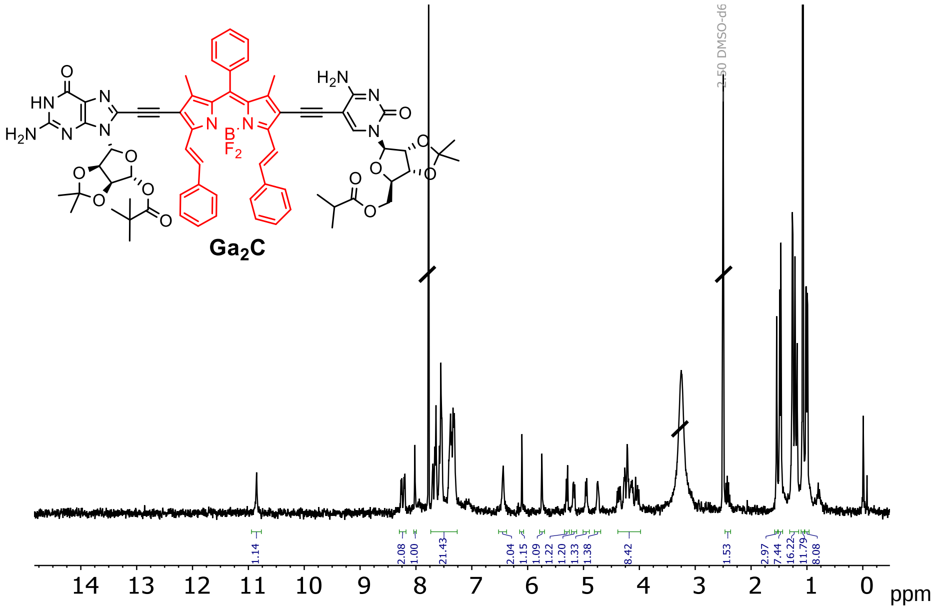


$^{1}$H-NMR spectrum of **Ga$_2$C** (300 MHz, $CDCl_3$/DMSO-*D$_6$*)

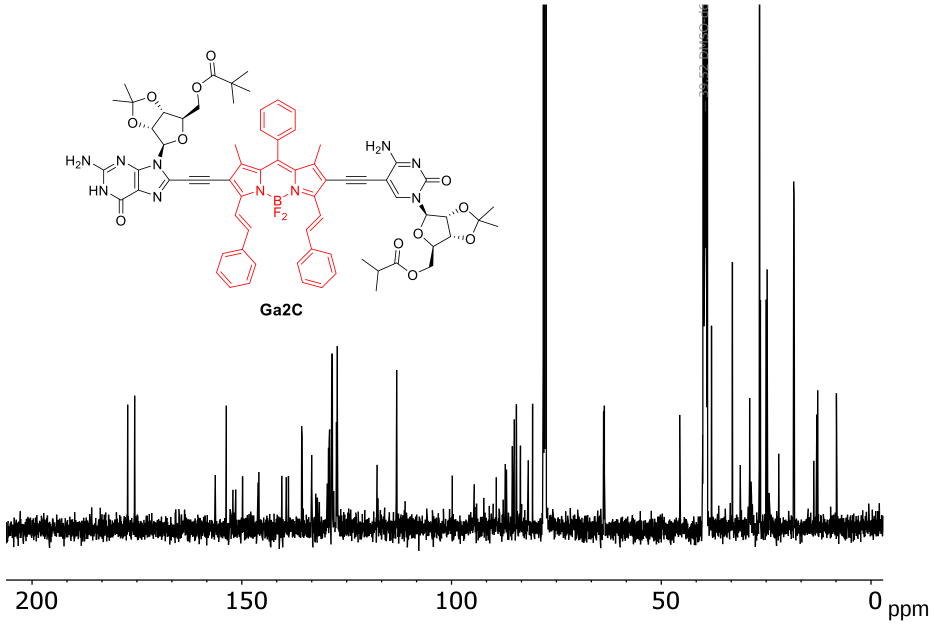


$^{13}$C-NMR spectrum of **Ga$_2$C** (125 MHz, DMSO-*D$_6$*)

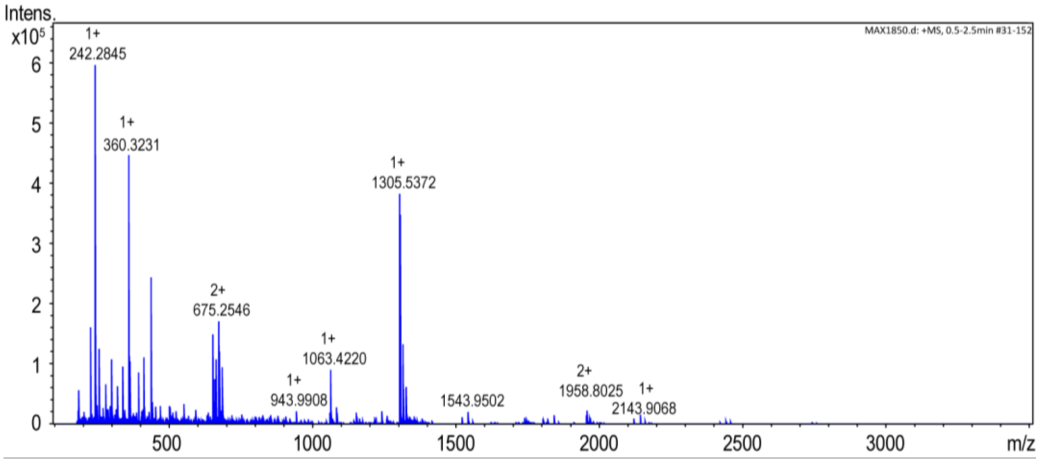


HRMS (ESI+) spectrum of **Ga$_2$C**

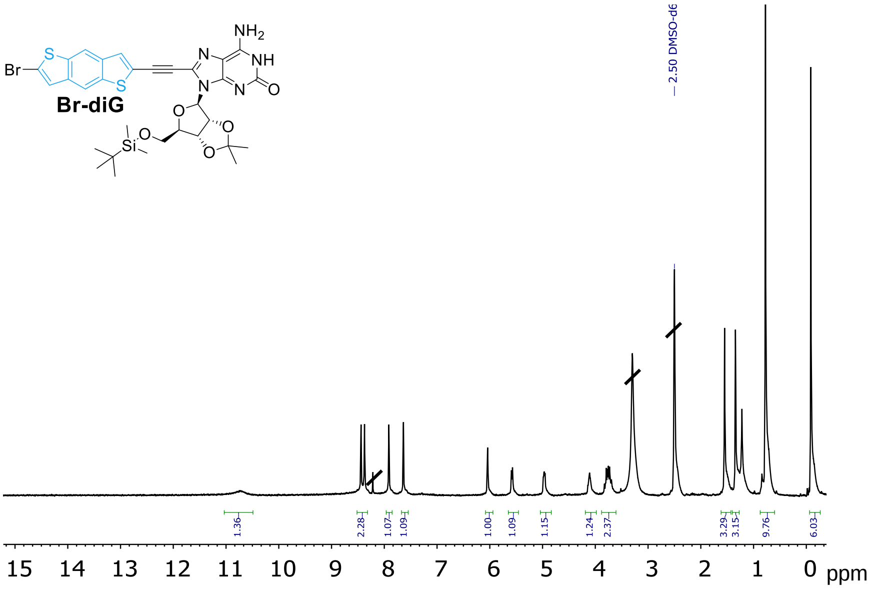


$^{1}$H-NMR spectrum of **Br-d*i*G** (300 MHz, DMSO-*D6*)

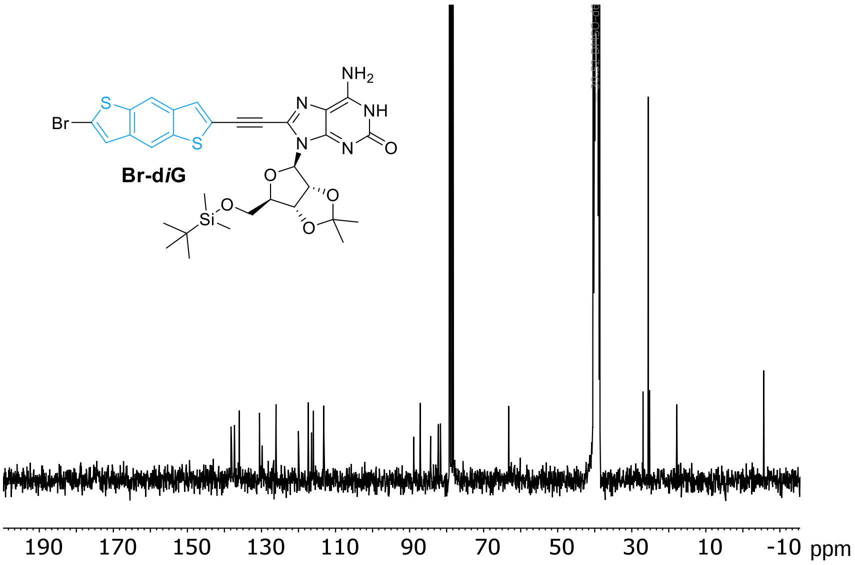


$^{13}$C-NMR spectrum of **Br-d*i*G** (76 MHz, DMSO-*D6*)

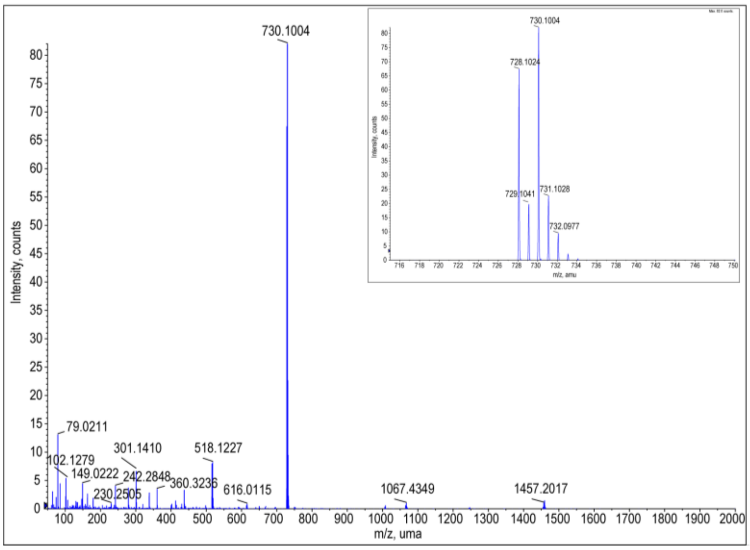


HRMS (ESI+) spectrum of **Br-d*i*G**

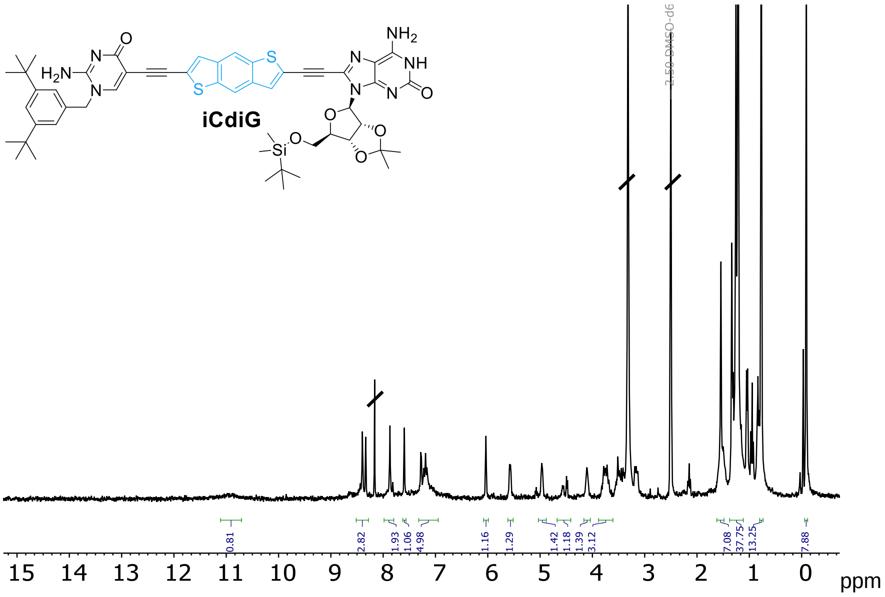


$^{1}$H-NMR spectrum of ***iCdiG*** (300 MHz, DMSO-*D*$_6$)

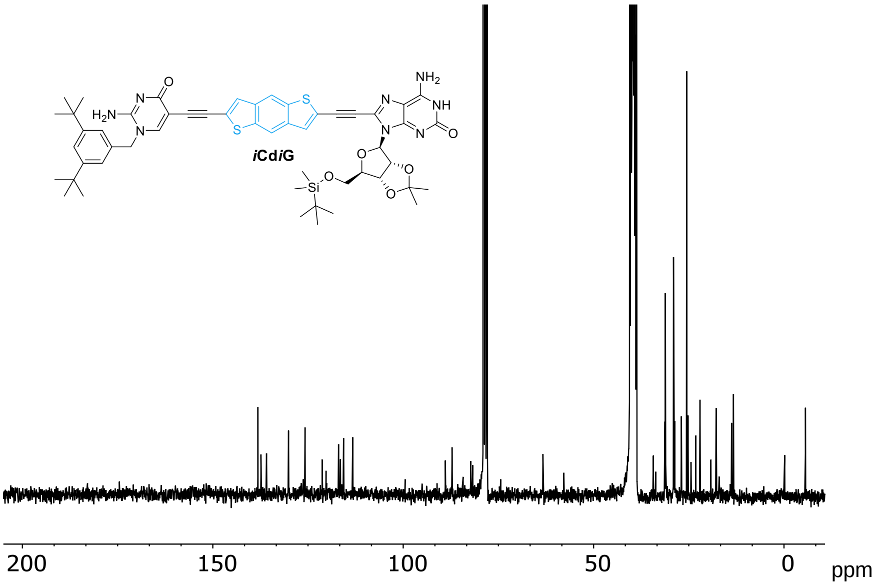


$^{13}$C-NMR spectrum of ***iCdiG*** (76 MHz, DMSO-*D*$_6$)

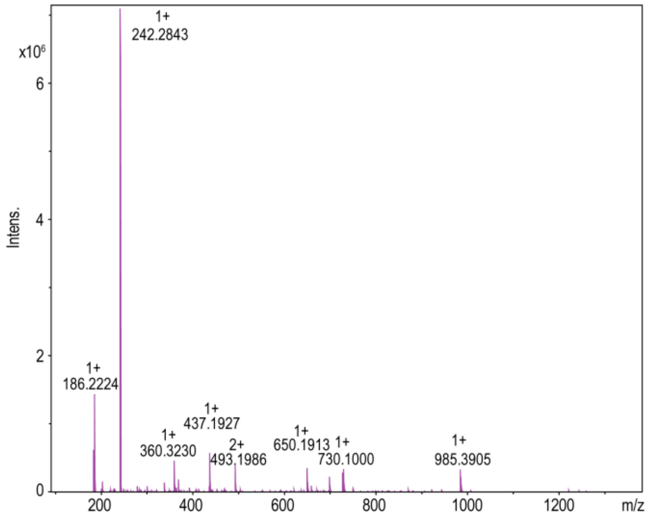


HRMS (ESI+) spectrum of ***iCdiG***

## S1. Building Speciation Profiles

Speciation curves were generated using the *Hyss* (Hyperquad Simulation and Speciation) software, version 4.0.31, developed by http://www.hyperquad.co.uk/index.htm. Simulations were built considering the following equilibrium constants (*K*; see Figure S1A) and effective molarity (*EM*) values:

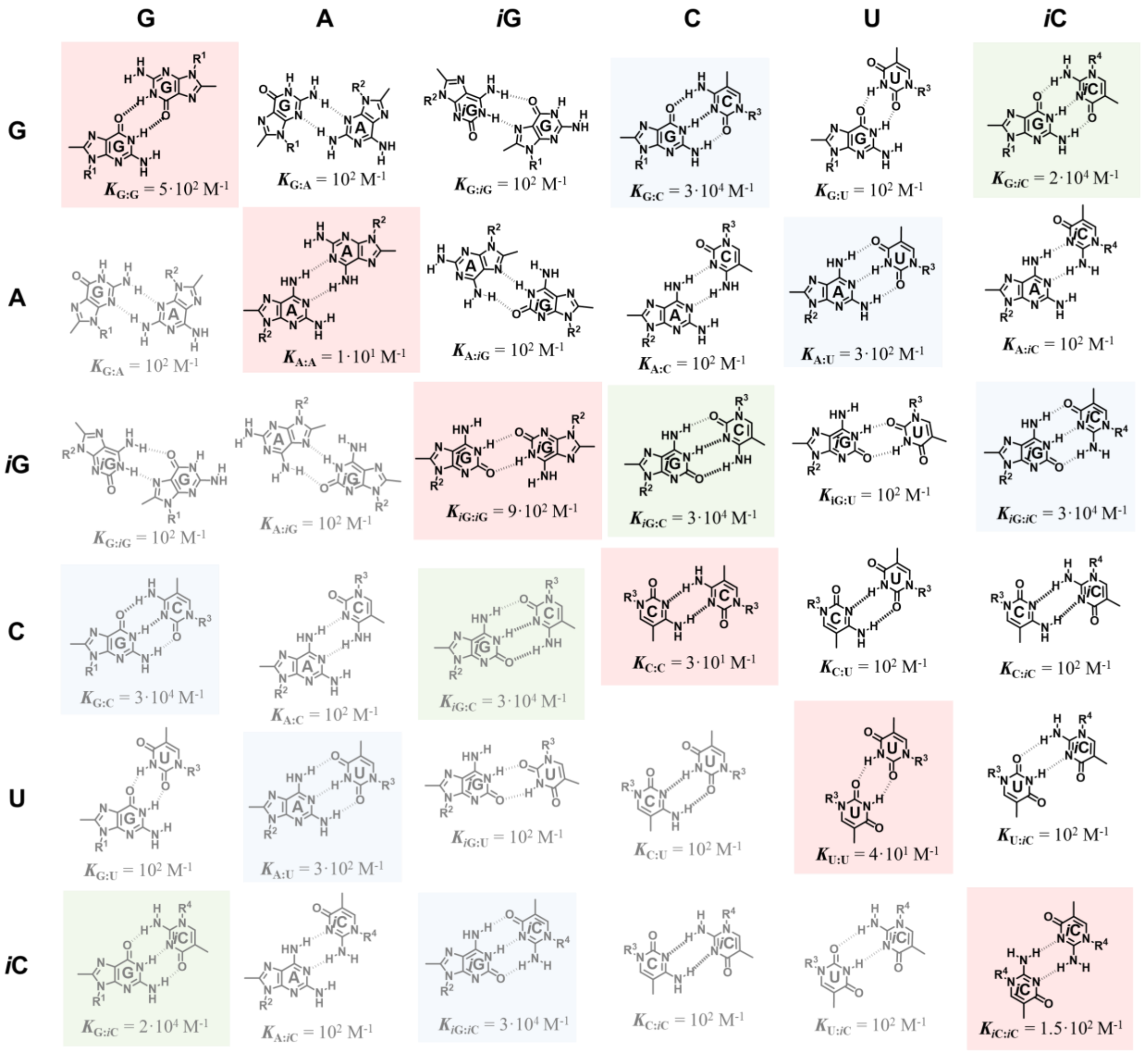


**Figure S1A**. *Dimerization (red-shadowed area) and association constants (in $CHCl_3$) between nucleobases used in the Hyss simulations.* The triply H-bonded Watson-Crick and reverse Watson-Crick are shown within blue- and green-shadowed areas, respectively. Please note that the A:U pair may also bind through reverse Watson-Crick interactions. For the non-complementary nucleobase pairs or the dimerizations, only one possible doubly H-bonded complex is represented, although there are many other possible configurations. Due to the nature of the diagram, the associated structures are duplicated (in grey), with the exception of the dimerizations.

1) Dimerization of mononucleosides with a given dimerization constant ($K_{G:G}$, $K_{iG:iG}$, $K_{A:A}$, $K_{U:U}$, $K_{C:C}$, $K_{iC:iC}$; red-shadowed area in Figure S1A), as determined in ref. 1 in $CHCl_3$ or in $CHCl_3/CCl_4$ 2:3.

2) Binding interactions between different mononucleosides with a given association constant in $CHCl_3$, $CHCl_3/CCl_4$ 2:3, or THF-$D_8$. For the triply H-bonded complementary regular ($K_{G:C}$, $K_{iG:iC}$, $K_{A:U}$; blue-shadowed area in Figure S1A) or reverse ($K_{G:iC}$, $K_{iG:C}$; green-shadowed area in Figure S1A) Watson-Crick pairs, the association constant values determined in ref. 1 and 2 were taken. For all unknown non-complementary

pairs, bound by *DA-AD* double H-bonding interactions ($K_{G:A}$, $K_{G:iG}$, $K_{G:U}$, $K_{A:iG}$, $K_{A:C}$, $K_{A:iC}$, $K_{iG:U}$, $K_{C:U,}$ $K_{C:iC,}$ $K_{U:iC}$ see Figure S1A below), an arbitrary association constant of $10^2$ $M^{-1}$ in $CHCl_3$ (which is probably a higher limit) or in $CHCl_3/CCl_4$ 2:3 was used.

3) The same association constants between the nucleobases were employed in the dinucleosides. In addition, *EM* values of $10^3$ M for *c*(GC)$_4$ and *c*(*i*G*i*C)$_4$ and a *EM* value of $10^{-2}$ M for *c*(AU)$_4$, as calculated in ref. 4, were used in the simulations.

Speciation curves were then obtained with these values using a total concentration window that goes from 1 M up to $10^{-7}$ M, as displayed in the main text or in this Supplementary Information.

Figure S1B completes Figure3 in the text and shows how the distribution of species in a hypothetical mixture of two monomers ($\mathbf{M^1}$ and $\mathbf{M^2}$) changes as a function of the magnitude of the association constant between them (*K*; horizontal direction) and/or as a function of the effective molarity of the $c(\mathbf{M^1})_4$ and $c(\mathbf{M^2})_4$ macrocycles (*EM*; vertical direction). These two monomers would resemble the experimentally studied **GC** + ***i*G*i*C** mixture in the sense that they are endowed with complementary H-bonding units at the edges, so that each monomer can bind to itself or to the other with identical association constant (*i.e.* $K(\mathbf{M^1{:}M^1}) = K(\mathbf{M^2{:}M^2}) = K(\mathbf{M^1{:}M^2}) = K$) to form supramolecular oligomers. In addition, each monomer can self-associate into cyclic tetramer species with identical effective molarities (*i.e.* $EM\ (c(\mathbf{M^1})_4) = EM\ (c(\mathbf{M^2})_4) = EM$).

In each graph, the population of $\mathbf{M^1}$ (or $\mathbf{M^2}$) monomers (red), $\mathbf{M^1{:}M^1}$ (or $\mathbf{M^2{:}M^2}$) self-associated linear oligomers (like $\mathbf{M^1}_2$, $\mathbf{M^1}_3$, or $\mathbf{M^1}_4$; light blue), $\mathbf{M^1{:}M^2}$ mixed linear oligomers (like $\mathbf{M^1}$:$\mathbf{M^2}$, $\mathbf{M^1}_2$:$\mathbf{M^2}$, $\mathbf{M^1}$:$\mathbf{M^2}_2$, $\mathbf{M^1}_2$:$\mathbf{M^2}_2$, $\mathbf{M^1}_3$:$\mathbf{M^2}$, or $\mathbf{M^1}$:$\mathbf{M^2}_3$; light green), or $c(\mathbf{M^1})_4$ (or $c(\mathbf{M^2})_4$) self-sorted cyclic species, is displayed as a function of the overall concentration. The population of the last cyclic species represents self-sorting fidelity. At the right or at the bottom, the different graphs in each row or column, respectively, are overlaid.

It is clear that narcissistic self-sorting is complete over a wider range of concentrations only when chelate cooperativity is sufficiently high, which can be achieved by increasing either *EM* or *K*. Otherwise, the cyclic assemblies are in equilibrium with non-sorted linear oligomers and, at low concentrations, with the unbound monomers.

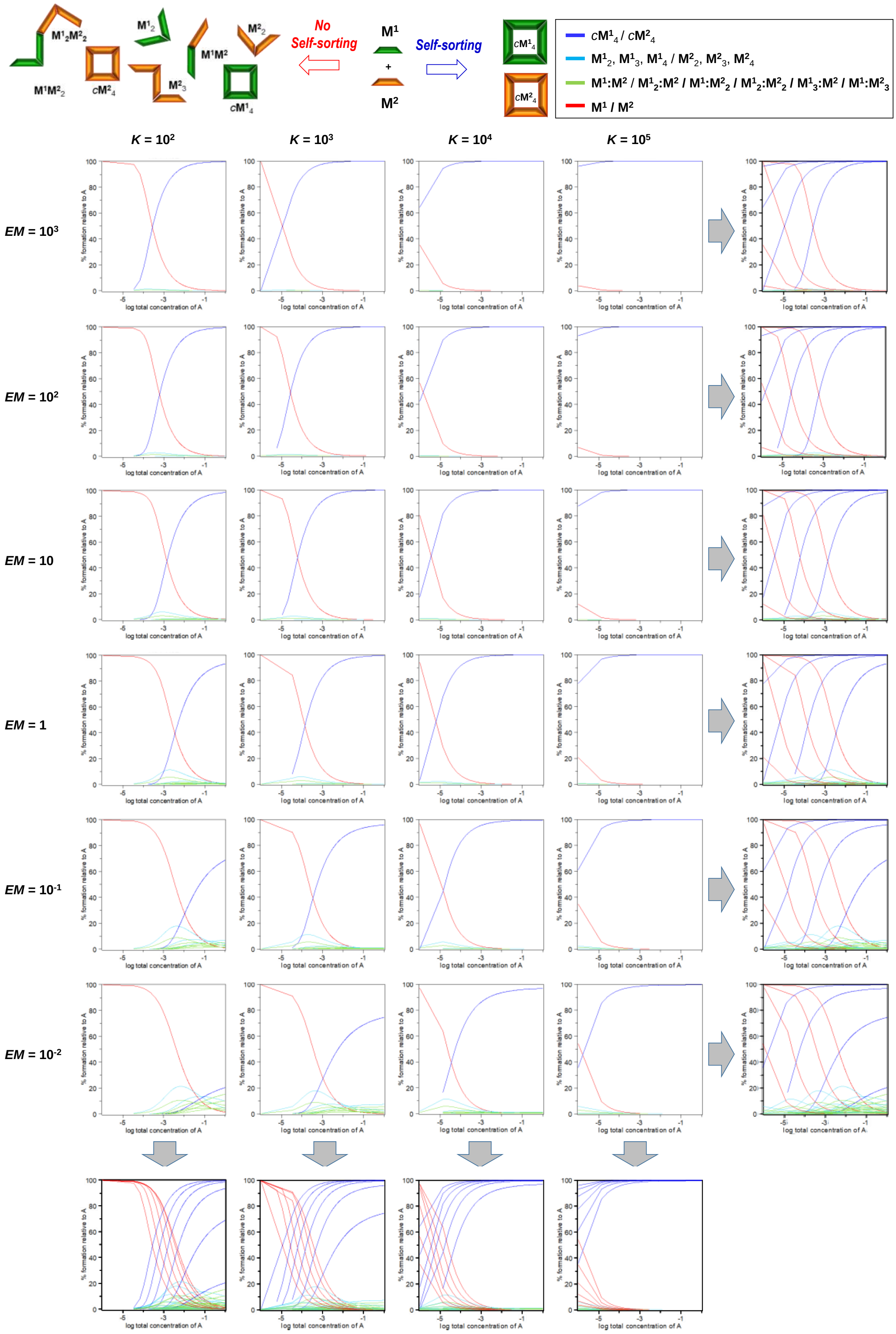


**Figure S1B**. *Self-sorting of a mixture of dinucleosides.* Speciation curves showing the distribution of species in a 1:1 hypothetical mixture of two dinucleoside monomers (called here $\mathbf{M^1}$ and $\mathbf{M^2}$) with the same supramolecular features as the experimentally studied **GC+*iGiC*** system, as a function of the association constant (*K*; horizontal direction) and/or the hypothetical effective molarity of both the *c*$\mathbf{M^1}_4$ and *c*$\mathbf{M^2}_4$ macrocycles (*EM*; vertical direction).

Figure S1C completes Figure 7 in the text and shows how the distribution of species in a **GC**+**G**+**C** 1:1:1 mixture changes as a function of the magnitude of the association constant between this Watson-Crick pair (*K*; horizontal direction) and/or as a function of the effective molarity of the $c(\mathbf{GC})_4$ macrocycle (*EM*; vertical direction).

It is clear that virtually complete narcissistic self-sorting would be achieved when chelate cooperativity is strong, that is, at high $K{\cdot}EM$ values (top-right corner in Figure S1C). Even though, complete self-sorting can only be achieved within an intermediate concentration window. For instance, at $K = 10^5$ $M^{-1}$ and $EM = 10^3$ M (top-right simulation) within the $10^{-6}$-$10^{-2}$ M concentration range, >95% of **GC** molecules are associated as cycles, while **G** and **C** establish an equilibrium between **G:C** associated and dissociated species. Lower concentrations obviously lead to dissociation of both bimolecular **G:C** complexes and cyclic assemblies. On the other hand, higher concentrations are against intramolecular associations and the trimolecular **C:GC:G** complex, integrating all species, start to compete. Such competition is more important as the *EM* of the cyclic system diminishes (see the evolution along the right column), so that, for instance, if *EM* would be $10^{-2}$ M (bottom-right simulation), the $c(\mathbf{GC})_4$ macrocycle could only reach a maximum of <75% relative abundance, and narcissistic self-sorting would be lost, even at relatively high *K* values. It is interesting to note, on the other hand, that the association constant *K* does not influence the relative abundance of **C:GC:G**, but instead dominates the relative abundance of dissociated *vs* associated species, either cyclic or non-cyclic (see the evolution along the top line).

If we now analyze the simulations shown in Figure S1C from the other corner (bottom-left), self-sorting is totally absent when both *K* and *EM* are low, and actually supramolecular association can only be achieved at relatively high concentrations. But again, even maintaining a low association strength, a decent degree of narcissistic self-sorting can be achieved if *EM* values are sufficiently high (see the evolution along the left column), so that the $c(\mathbf{GC})_4$ macrocycle could reach 90% relative abundance at $K = 10^2$ $M^{-1}$ and $EM = 10^3$ M (top-left simulation) close to a $10^{-2}$ M concentration.

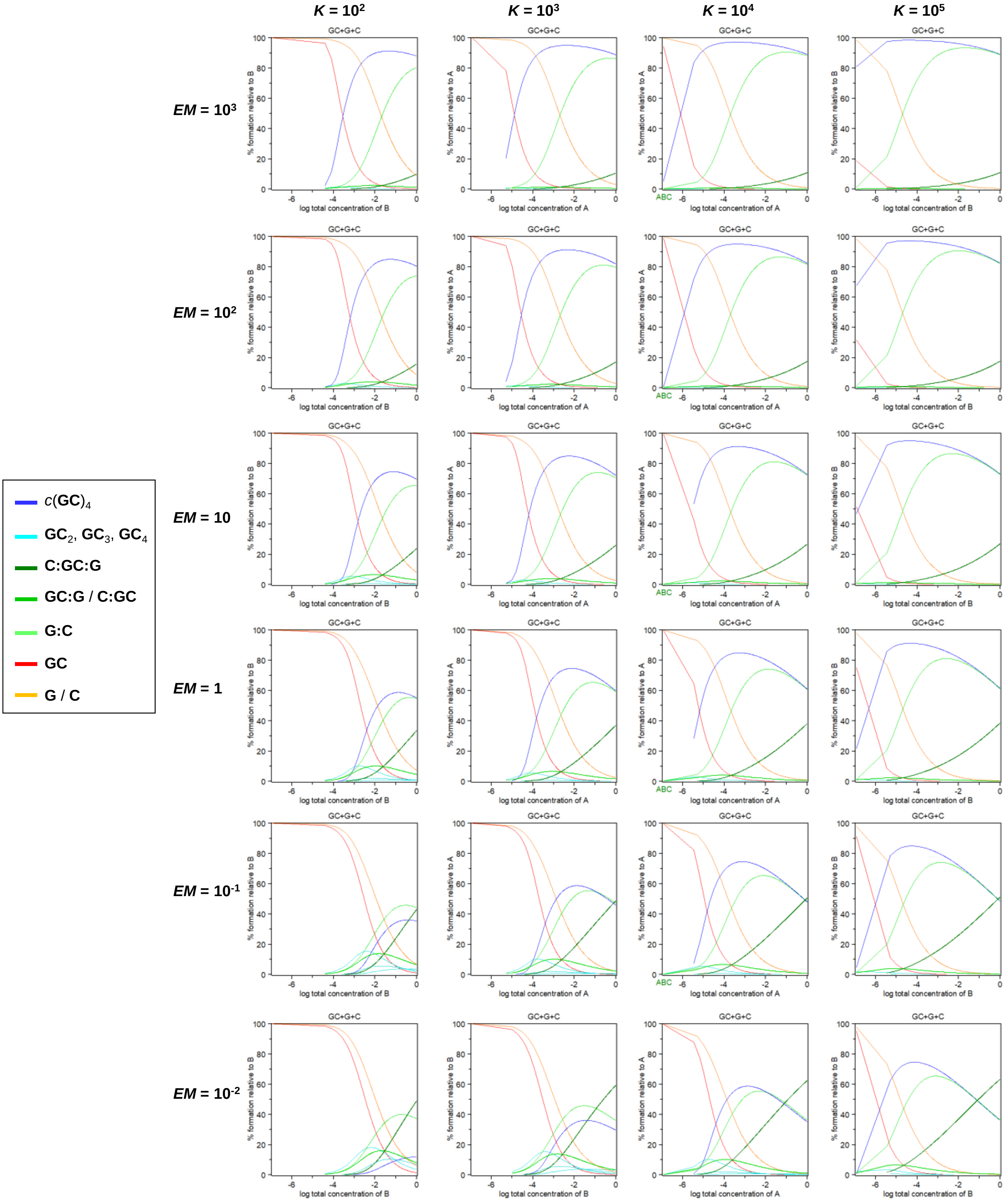


**Figure S1C**. *Self-sorting of a mixture of mono- and dinucleosides sharing the same Watson-Crick interaction.* Speciation curves showing the distribution of species in a **GC**+**G**+**C** 1:1:1 mixture changes as a function of the magnitude of the association constant (*K*; horizontal direction) and/or as a function of the hypothetical effective molarity of the *c*(**GC**)$_4$ macrocycle (*EM*; vertical direction).

## S2. $^1$H NMR and NOESY Spectroscopy Measurements

### S2.1. $^1$H NMR and NOESY Spectroscopy Measurements of Mononucleoside Mixtures

We started by examining the $^1$H NMR spectra in 1:1 mixtures of complementary mononucleosides (**G**+**C**, **A**+**U** and ***i*G**+***i*C**) at a fixed concentration ($1.0 \cdot 10^{-2}$ M) and temperature (298 K) in $CDCl_3$. As can be observed in Figure S2A, H-bonding formation between complementary pairs becomes evident when examining the upfield shift experienced by the different protons involved and their cross-peaks in NOESY experiments. For instance in the complementary ***i*G**+***i*C**, **G**+**C** and **A**+**U** mixtures (Figure S2Aa), the ***i*G**-amide and ***i*C**-amine protons appear at 13.9 and 10.2 ppm, the **G**-amide and **C**-amine protons at 13.2 and 8.3 ppm, while the **U**-imide and **A**-amine protons are found at 11.8 and 6.3 ppm, respectively. These chemical shifts, when compared to those of the fully bound species,[4] indicate that **G:C** and ***i*G:*i*C** H-bonded pairs are almost fully formed in these conditions, but the relative abundance of the **A**:**U** pair with respect to unbound **A** and **U** is small in these conditions. Still, as shown at Figure S2A, clear NOESY cross-peaks were detected between all these relevant H-bonded protons in each complementary **G:C**, ***i*G:*i*C** and **A**:**U** pair.

These mononucleoside pairs were further combined in 1:1:1:1 mixtures (**G**+**C**+**A**+**U** and **G**+**C**+***i*G**+***i*C**). In the case of the **G**+**C**+**A**+**U** mixture, no significant changes in the $^1$H NMR spectrum were detected Figure S2Ab. Only for the **G**+**C**+***i*G**+***i*C** combination, a slight broadening and shift of some signals was observed. 2D NOESY experiments performed in the same conditions could confirm the proximity of the relevant H-bonded protons in the complementary pairs and provide an assessment whether they self-sort or not in their 1:1:1:1 mixtures. As shown at Figure S2Ac, the **G**+**C**+***i*G**+***i*C** mixture exhibit cross-peaks between all possible combinations of Watson Crick and reverse Watson-Crick pairs (**G**:**C**, ***i*G**:***i*C**, **G**:***i*C** and ***i*G**:**C**), but also between **G** and ***i*G**. To our surprise, the **G**+**C**+**A**+**U** mixture also displayed cross-peaks between all possible pairs (**G**:**C**, **A**:**U**, **G**:**U**, **A**:**C**, **G**:**A** and **C**:**U**). This may be due to the formation of non-complementary (or mismatched) pairs and/or to the association in higher-order species (trimolecular complexes. etc.), but in any case these results clearly show that no binding selectivity is observed in the quaternary mononucleoside mixtures and any kind of self-sorting phenomena is absent.

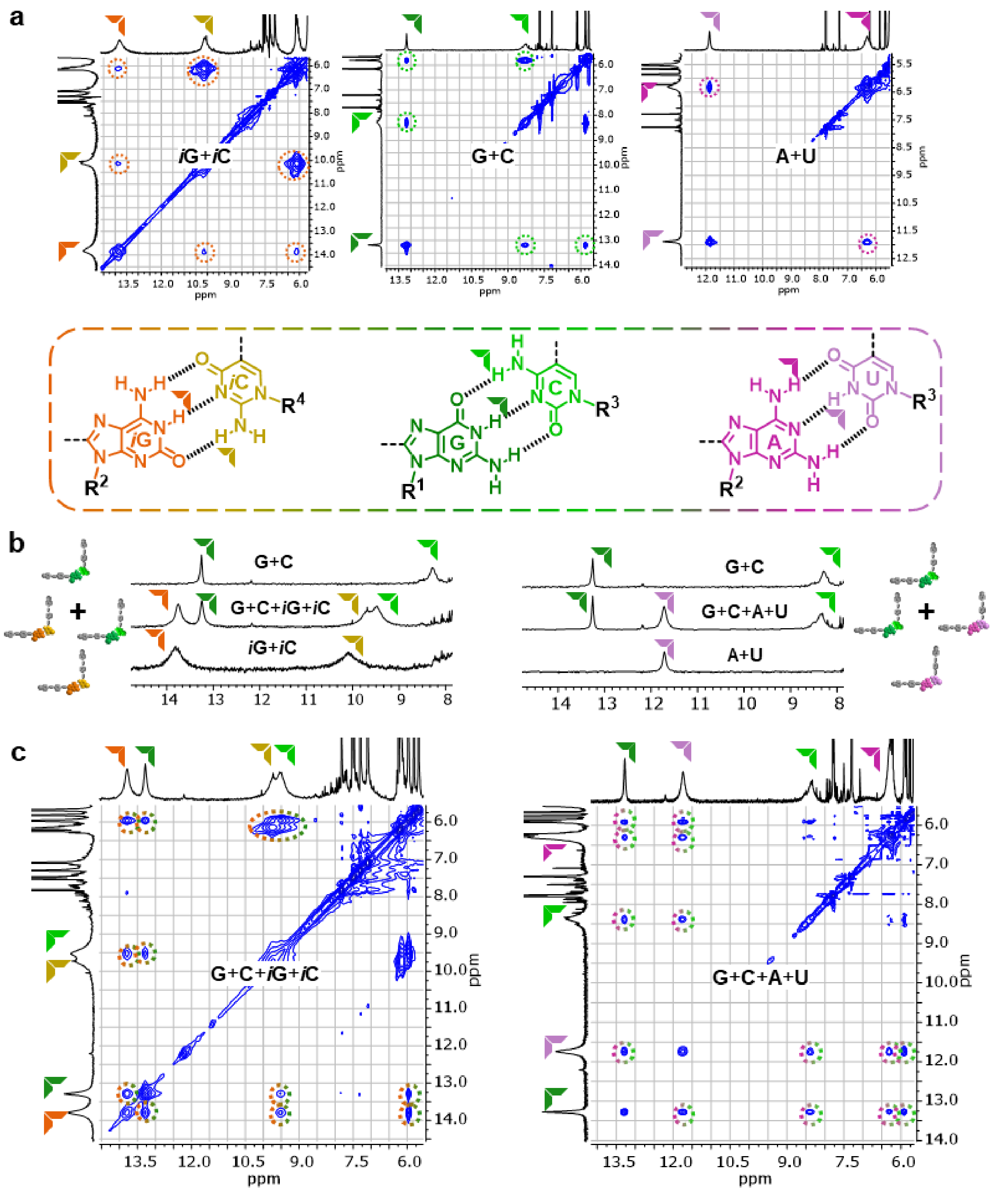


**Figure S2A**. *Quaternary mononucleoside combinations examined by $^1$H and 2D NOESY NMR*. (**a**,**c**) From left to right: downfield region of the NOESY NMR spectrum of 1:1 mixtures of ***i*G**+***i*C**, **G**+**C** and **A**+**U**, and 1:1:1:1 mixtures of **G**+**C**+***i*G**+***i*C** and **G**+**C**+**A**+**U**, respectively. (**b**) Downfield region of the $^1$H NMR spectra in $CDCl_3$ showing the chemical shift of the complementary 1:1 Watson-Crick pair combinations (**G**+**C**, ***i*G**+***i*C**, **A**+**U**) and their 1:1:1:1 mixtures (**G**+**C**+***i*G**+***i*C** and **G**+**C**+**A**+**U**). $C = 1.0 \times 10^{-2}$ M in $CDCl_3$ and $T = 298$ K in all cases. (**c**) NOESY spectra of a 1:1:1:1 mixture of **G**+**C**+***i*G**+***i*C** ($CDCl_3$; $10^{-2}$ M; 298 K) and a 1:1:1:1 mixture of **G**+**C**+**A**+**U** ($CDCl_3$; $10^{-2}$ M; 298 K).

## S2.2. $^1$H NMR and NOESY Spectroscopy Measurements of Dinucleoside Mixtures

We then turned our attention to the behavior of 1:1 mixtures of dinucleosides in similar conditions. We first recorded the $^1$H and NOESY NMR spectra of the individual dinucleosides (Figure S2Ba), where the relevant protons are now forming strong H-bonds according to their chemical shifts. For instance, the *i*G-amide, *i*C-amine and *i*G-amine protons appear at 13.7, 10.4 and 9.8 ppm, respectively, the G-amide and C-amine protons at 13.4 and 10.0 ppm, while the U-imide and the two A-amine protons are now found at 14.0, 8.6 and 7.4 ppm, respectively.

As shown in Figure S2Bb, mixing the non-complementary **GC** + **AU** dinucleosides produced no change in their $^1$H NMR spectra. NOESY experiments furthermore reveals that G only binds to C, while A only binds to U (Figure S2Bc; right). Looking at the G-amide signal, cross-peaks are only seen with the two C-amine protons (dashed green circles). Similarly, when examining the U-imide signal, cross-peaks were only seen with the A-amine protons (dashed pink circles). Therefore, the **GC + AU** mixture exhibits clear narcissistic self-sorting characteristics, which is what we would expect in view of the non-complementary H-bonding patterns of the 2 Watson-Crick pairs involved.

Now, in the case of the **GC** + ***i*G*i*C** mixture, the H-bonding patterns are complementary and these 4 bases could in principle bind through all combinations of Watson Crick and reverse Watson-Crick pairs, which would lead to a complex mixture of cyclic and open oligomers. However, $^1$H NMR (Figure S2Bb) reveals that, when mixed, the relevant H-bonded protons do not suffer significant changes, and the spectrum resembles the sum of both cyclic tetramers separately. On the other hand, NOESY spectra (Figure S2Bc; left) clearly show that only their respective cyclic tetramers are formed, where G only binds to C (dashed green circles) whereas iG binds exclusively to iC (dashed orange circles), and G:iC or iG:C cross-peaks were not detected. Therefore, in this particular case, *narcissistic self-sorting is clearly not ruled by H-bonding complementarity, but by chelate cooperativity*, that is, by the strong tendency of both dinucleoside molecules to form cyclic tetramers with high *EM*s. Only when **GC** and ***i*G*i*C** associate independently, each cyclic tetramer species can be assembled because a Watson-Crick 90° angle is required.

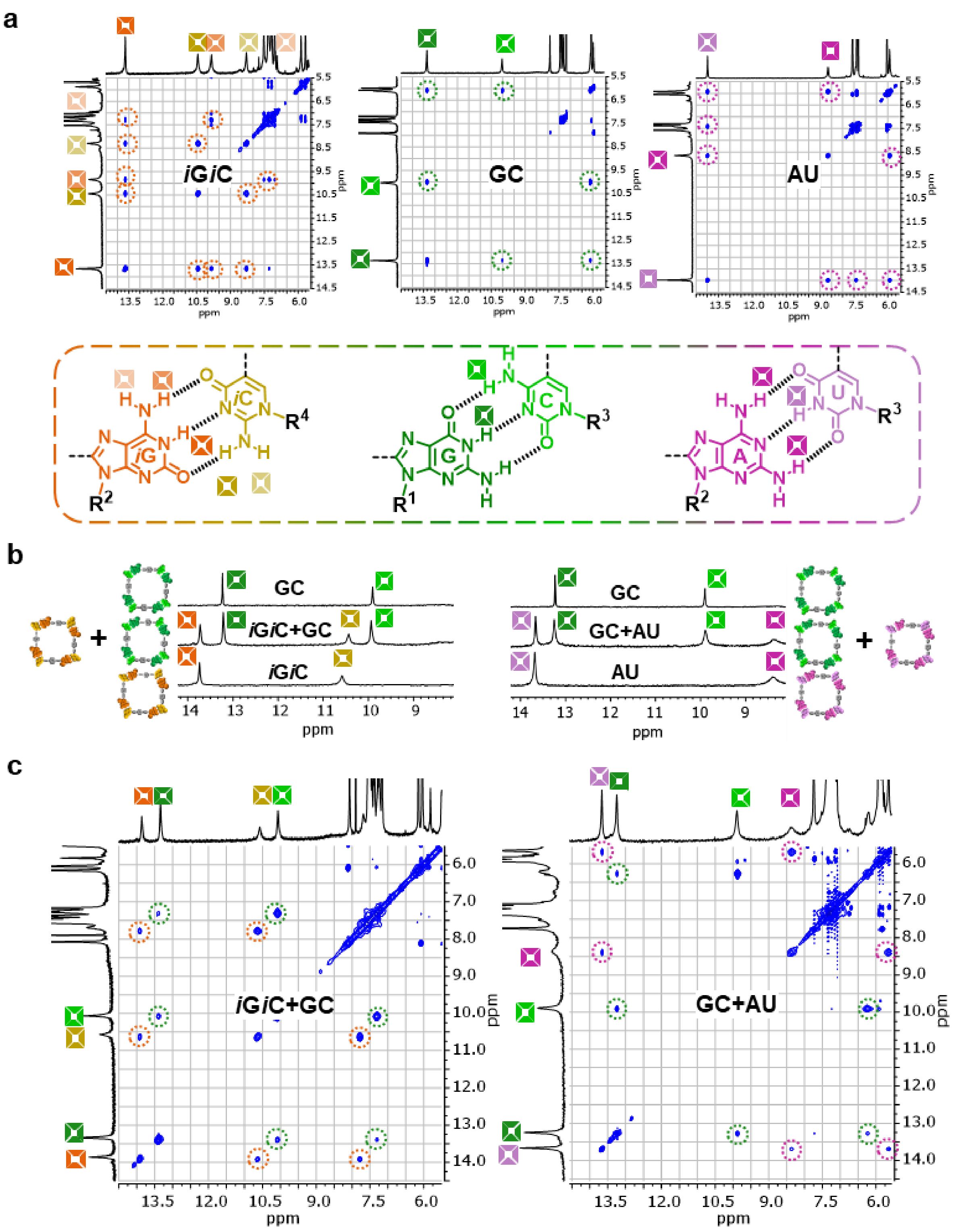


**Figure S2B**. *Binary dinucleoside combinations examined by $^1$H and 2D NOESY NMR*. *Cyclic tetramer self-sorting.* From top to bottom, downfield region of the (**a**) $^1$H NMR spectra in $CDCl_3$ in all cases with the exception of **GC** + ***iGiC*** and ***iGiC***, where THF-$D_8$ was employed. (**b**) NOESY NMR spectra of ***iGiC*** ($CD_2Cl_2$ (+1% DMSO) at 238 K), **GC** ($CDCl_3$), and **AU** ($CDCl_3$ at 253 K), and their respective 1:1 mixtures: (**c**) **GC** + ***iGiC*** (THF-$D_8$) and **AU** + **GC** ($CDCl_3$:$CCl_4$ (2:3)) showing cross-peaks between the H-bonded proton signals. In all cases $C$ = 1.0 x $10^{-2}$ M.

Unfortunately, we were not able to properly study the 1:1 mixtures of ***iGiC*** + **AU** (or the 1:1:1 ternary mixtures of the three dinucleosides **GC** + ***iGiC*** + **AU**) due to a combination of solubility and stability problems. On one hand, a solvent of lower polarity than $CDCl_3$ or low temperatures are needed to assemble *c***AU**$_4$ quantitatively. Figure S2Ca shows the downfield region of the $^1$H NMR spectra of a 1:1 **GC** + **AU** mixture. In 100% $CDCl_3$, while *c***GC**$_4$ is formed quantitatively, the *c***AU**$_4$ assembly is in equilibrium with mixtures of short open oligomers (signal around 10.5 ppm; see our previous work).[4] In 100% $CCl_4$, the sample containing these two dinucleosides was not totally dissolved and the spectrum is not well-resolved. As increasing amounts of $CDCl_3$ were added to $CCl_4$ while maintaining overall concentration, the solubility of both monomers is enhanced and *c***AU**$_4$ could be formed quantitatively, in the presence of *c***GC**$_4$, in a $CDCl_3$/$CCl_4$ (2:3) mixture. However, and unfortunately, ***iGiC*** revealed rather broad $^1$H NMR spectra already in 100% $CDCl_3$ (or $CD_2Cl_2$) and the samples are not exceptionally soluble. As shown in Figure S2Cb, this can be solved by the addition of a tiny amount of DMSO-$D_6$ or directly using a more polar solvent such as THF-$D_8$. Both problems combined precluded the NMR measurement of 1:1 mixtures of ***iGiC*** + **AU**. In a first scenario, where we used $CDCl_3$:$CCl_4$ mixtures as solvent to assemble *c***AU**$_4$ quantitatively, ***iGiC*** was completely insoluble. In a second scenario where we employed a small amount of DMSO-$D_6$ or 100% THF-$D_8$ to solubilize ***ciGiC***$_4$ properly, **AU** would not be able to form discrete cyclic systems and the monomer would be the most abundant entity present in such relatively polar solvents.

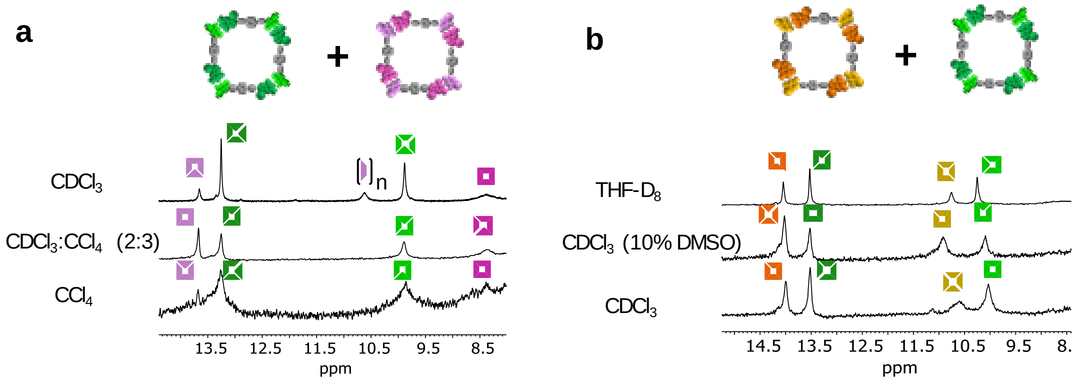


**Figure S2C**. *Tuning solvent composition for dinucleoside mixtures with **AU** or **iGiC**.* Downfield region of the $^1$H NMR spectra of (**a**) **GC** + **AU** in $CCl_4$, $CDCl_3$, and mixtures. (**b**) **GC** + ***iGiC*** in $CDCl_3$, a mixture with DMSO-$D_6$ and in THF-$D_8$. In all cases $C$ = 1.0 x $10^{-2}$ M and $T$ = 298 K.

It should be noted that the absence of narcissistic self-sorting phenomena would not only lead to open oligomeric species. Other cyclic tetramers could be formed by double H-bonding A:C or G:U association, as shown in Figure S2Da. In addition, if the 210° reverse Watson-Crick G:*i*C or *i*G:C association is established, an unstrained cyclic dodecamer may be formed in solution as well, as shown in Figure S2Db. However, it is clear from the NOESY experiments that these mixed cyclic assemblies are not formed. This is, on the other hand, quite logical, since in the first case only two H-bonds are formed and the binding strength should decrease with respect to a regular Watson-Crick H-bonding. In addition, two-component cyclic species should enjoy much lower *EM* values than one-component macrocycles. In the second case, additionally, the *EM* of an hypothetic dodecamer, being made of a much higher number of monomers, should decrease even further, certainly much lower than those observed for our cyclic tetramers.[11]

[11] Ercolani, G. *Struct. Bond.* **2006**, *121*, 167–215

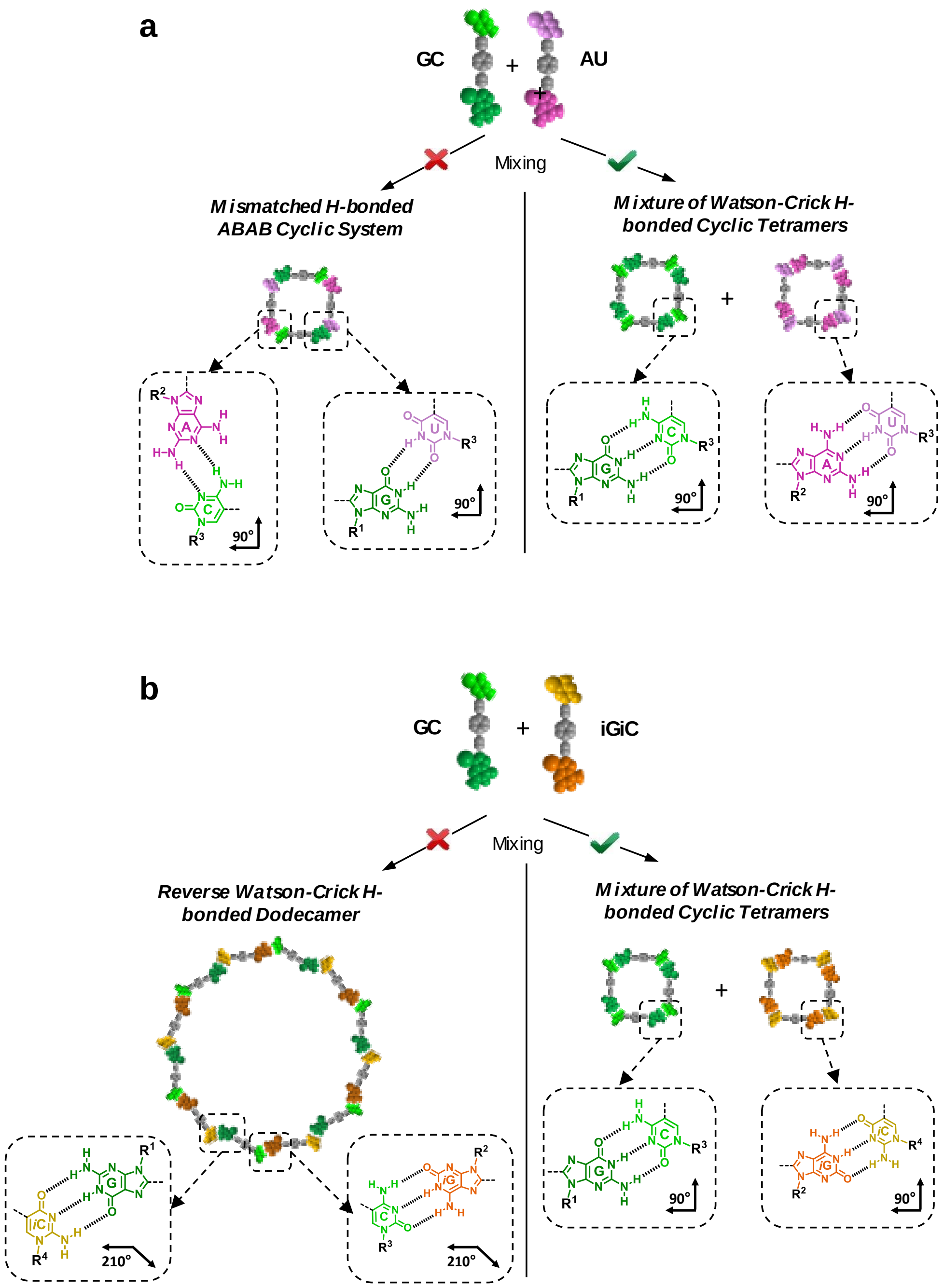


**Figure S2D**. *Possible formation of other H-bonded macrocycles.* (**a**) *Mismatched vs Watson-Crick H-bonding patterns:* (**b**) *Watson-Crick vs Reverse Watson-Crick H-bonding:*

## S3. CD and Emission Spectroscopy Measurements

## S3.1. CD and Emission Spectroscopy Measurements of Mononucleoside Mixtures

While NMR experiments already provided a reasonably clear picture of the self-assembly of mixtures of mono- and dinucleosides, we complemented these studies with CD and emission spectroscopy experiments using donor and acceptor FRET pairs.[5] In these measurements, concentration was lowered to the $10^{-4}$-$10^{-6}$ M regime and toluene was used to increase binding strength between base pairs (particularly the weaker A:U pair). As we determined in previous studies,[2] association constants in this apolar solvent are increased in about one order of magnitude with respect to $CHCl_3$ (over $10^5$ $M^{-1}$ for G:C/iG:iC and over $10^3$ $M^{-1}$ for the A:U pair).

So, CD and emission spectroscopy work in these conditions as complementary tools to study our self-assembling mixtures. First, CD reveals the existence of macrocycles since, as determined in all of our previous work,[1,4,5,12] the dinucleoside molecules reveal clear Cotton effects only upon cyclotetramerization, and the monomer, possible open oligomers or bimolecular Watson-Crick pairs are not CD-active. This is attributed to the fact that cyclization, in contrast to unbound molecules or open oligomers, fixes to a higher extent the conformation of the π-conjugated backbone. Since we have endowed our dinucleosides with chromophores that absorb in different regions of the spectral window, it should be easy to differentiate macrocycles made of any of these dyes by CD spectroscopy. Second, if mono- or dinucleosides that bear FRET-complementary donor and acceptor pairs (*i.e.* **d** and $\mathbf{a_1}$ or $\mathbf{a_1}$ and $\mathbf{a_2}$) are in close proximity because of intermolecular binding, an energy transfer process would be activated that will quench donor fluorescence emission and, most often, enhance acceptor emission.

We followed the same rationale as in the previous NMR experiments: the spectroscopic features of mononucleoside complementary pairs or of dinucleosides were examined first at a given concentration, and then the relevant mixtures were generated at that concentration and spectroscopic changes were monitored with time until the equilibrium was reached. Therefore, stock solutions of the mono- and dinucleosides were prepared and divided in two fractions: one of them was diluted to reach the desired concentration of the individual monomers whereas the remaining stock fractions were mixed. Thus, depending on whether they are employed in binary, ternary or quaternary mixtures, stock solutions were prepared doubling, tripling and quadrupling, respectively, the concentration at which the experiment will be carried out

We again started examining the mononucleoside mixtures by emission spectroscopy, since they are not CD active. We 1:1-mixed complementary pairs first (*i.e.* G+C, A+U and *i*G+*i*C) bearing either FRET donor or acceptor functions and recorded their emission spectra at the appropriate excitation wavelengths. Then, we mixed some of these combinations of complementary mononucleosides, in which one pair now bears the energy donor function and the other pair the acceptor chromophore, so as to generate the final 1:1:1:1 mixtures, and recorded their emission spectra in the same conditions, including solvent, temperature, concentration, excitation wavelength and any other instrument settings.

[12] Montoro-García, C.; Mayoral, M. J.; Chamorro, R.; González-Rodríguez, D. *Angew. Chem. Int. Ed.* **2017**, *56*, 15649–15653.

Figure S3A shows an example of three of these combinations. For instance, Figure S3Aa,b display the emission spectra of the **dG**+**dC**+**a$_1$G**+**a$_1$C** and **d*i*G**+**d*i*C**+**a$_1$G**+**a$_1$C** mixtures, respectively, compared to the emission spectra of their parent solutions at the same concentration. In both cases, emission intensity in the donor area is noticeably quenched, which suggests that a FRET process becomes active in these mixtures. This is likely due to the formation of **dG**:**a$_1$C** and **dC:a$_1$G** pairs, in the first case, and **d*i*G**:**a$_1$G** and **d*i*C:a$_1$C** pairs in the second case, where FRET donor and acceptor dyes are interacting strongly. Self-sorting is therefore absent in these control mixtures due to the fact that we are not employing two pairs of self-complementary nucleobases.

Now, when analyzing the **dA**+**dU**+**a$_1$G**+**a$_1$C** 1:1:1:1 mixture (Figure S3Ac), where two pairs of self-complementary nucleobases are used, donor emission is not quenched appreciably, indicating that the Watson-Crick H-bonded **dA**:**dU** and **a$_1$G**:**a$_1$C** are the most abundant complexes in solution and thus that self-sorting operates to some extent. This result contrasts what was previously observed in the NOESY experiments at higher concentrations in $CDCl_3$, where we concluded that self-sorting mediated by H-bond complementarity was absent or was not very strong, but it is true that the experimental conditions, molecules and techniques employed are quite different.

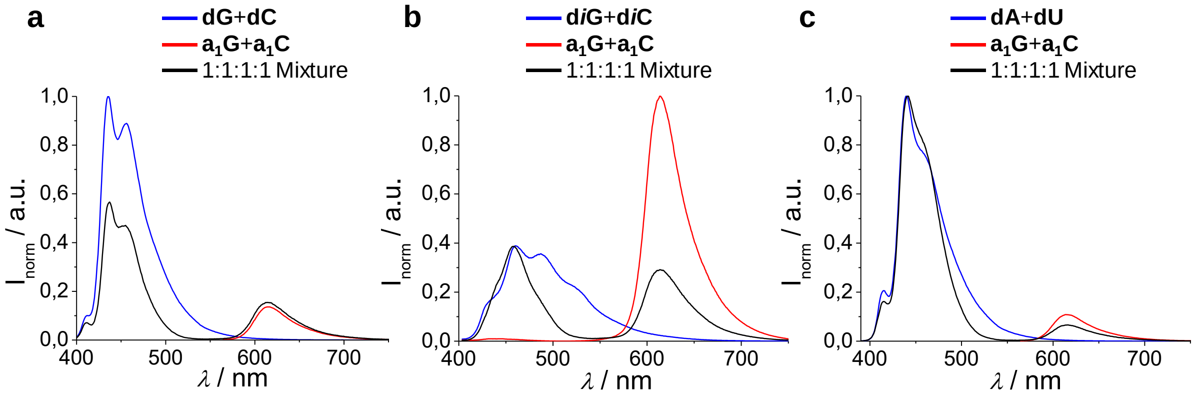


**Figure S3A**. *Evaluation of self-sorting in several 1:1:1:1 mononucleoside mixtures.* (**a**) Emission spectra ($\lambda_{exc}$ = 369 nm) of the **dG**+**dC** and **a$_1$G**+**a$_1$C** mixtures and their combination. (**b**) Emission spectra ($\lambda_{exc}$ = 397 nm) of the **d*i*G**+**d*i*C** and **a$_1$G**+**a$_1$C** mixtures and their combination. (**c**) Emission spectra ($\lambda_{exc}$ = 368 nm) of the **dA**+**dU** and **a$_1$G**+**a$_1$C** mixtures and their combination. In all cases the concentration of each nucleoside in toluene was $5.0 \cdot 10^{-5}$ M and *T* = 298 K.

## S3.2. CD and Emission Spectroscopy Measurements of Dinucleoside Mixtures

These results were then contrasted to the behavior of the 1:1 dinucleoside mixtures in the same conditions (Figures S3B-E). We again first performed control experiments in which energy donor and acceptor couples were combined in monomers having the same base pairs (*i.e.* **GdC**+**Ga$_1$C** and **AdU**+**Aa$_1$U**) and recorded the spectroscopic changes experienced by the system as a function of time until chemical equilibrium is reached. At equilibrium, a statistical mixture of six different cyclic tetramers should be formed (see Figure S3B), since the central π-conjugated blocks have identical lengths and are end-capped with the same nucleobases. Donor and acceptor moieties are closely positioned in some of these macrocycles, thus allowing for resonance energy transfer to take place, which should be evidenced by a decrease of donor emission and, frequently, an increase in acceptor emission. It is interesting to note that, in contrast to what was seen with the mononucleosides, equilibrium is reached very slowly with these dinucleoside mixtures in toluene, within a timescale of several hours, which underlines the extraordinarily high kinetic stability of the cyclic assemblies. When performing the same experiments in $CHCl_3$ or THF, equilibrium was instead reached within a few minutes. Figure S3Ba and b show, respectively, the comparison of the emission spectra of **GdC**, **Ga$_1$C** and their 1:1 mixture, on one hand, and **AdU**, **Aa$_1$U** and their 1:1 mixture, on the other. It is clear that donor emission is significantly quenched in both cases, confirming the expected FRET phenomena and thus the formation of the (statistical) mixture of macrocycles due to the absence of self-sorting. Donor quenching is stronger for the G-C dinucleoside combination because cyclic tetramers are formed quantitatively in these experimental conditions, whereas, as determined in our previous work,[5] cyclotetramerization is not complete for the A-U monomers due to the weaker A:U interaction and lower *EM* values.

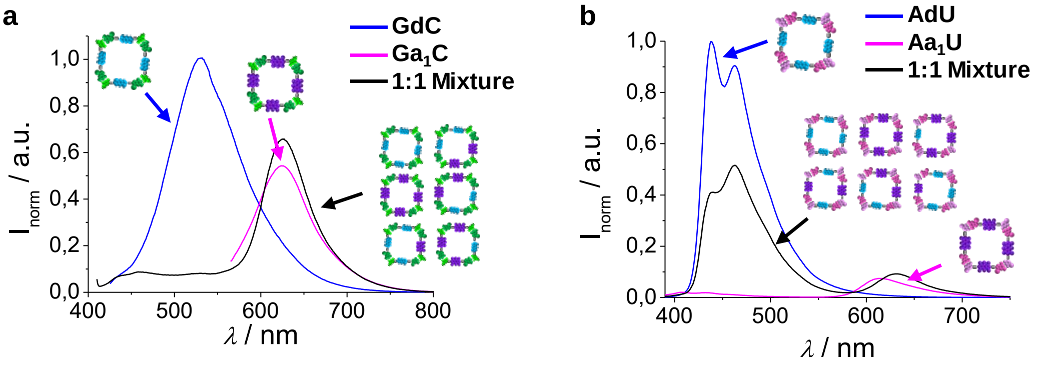


**Figure S3B**. *Evaluation of self-sorting in 1:1 dinucleoside mixtures with the same nucleobase pairs.* (**a**) Emission spectra ($\lambda_{exc}$ = 385 nm) of **GdC**, **Ga$_1$C** and their combination. (**b**) Emission spectra ($\lambda_{exc}$ = 360 nm) of **AdU**, **Aa$_1$U** and their combination. In all cases the concentration of each dinucleoside in toluene was $5.0 \cdot 10^{-5}$ M and $T$ = 298 K.

We then studied the scenario where the bases in the dye monomers are different. **GdC**+**Aa$_1$U** and **AdU**+**Ga$_1$C** mixtures where examined first (Figure S3Ca-b). In sharp contrast to what was observed before, negligible changes were detected over a period of 24 hours in the emission or CD spectra when these dinucleoside combinations were mixed together at the $10^{-4}$ - $10^{-6}$ M concentration range in toluene or $CHCl_3$. This indicated that a strong narcissistic self-sorting process takes place in solution, each dinucleoside interacting only with itself in the form of cyclic tetramers. The same results were found when combining iG-iC and A-U dinucleosides (Figure S3Cc) or changing the donor-acceptor FRET pair from **d**-**a$_1$** to **a$_1$**-**a$_2$** (Figure

S3Cd). This is in full agreement with the NMR results and confirms narcissistic self-sorting between cyclic tetramers when the bases are non-complementary in their H-bonding pattern.

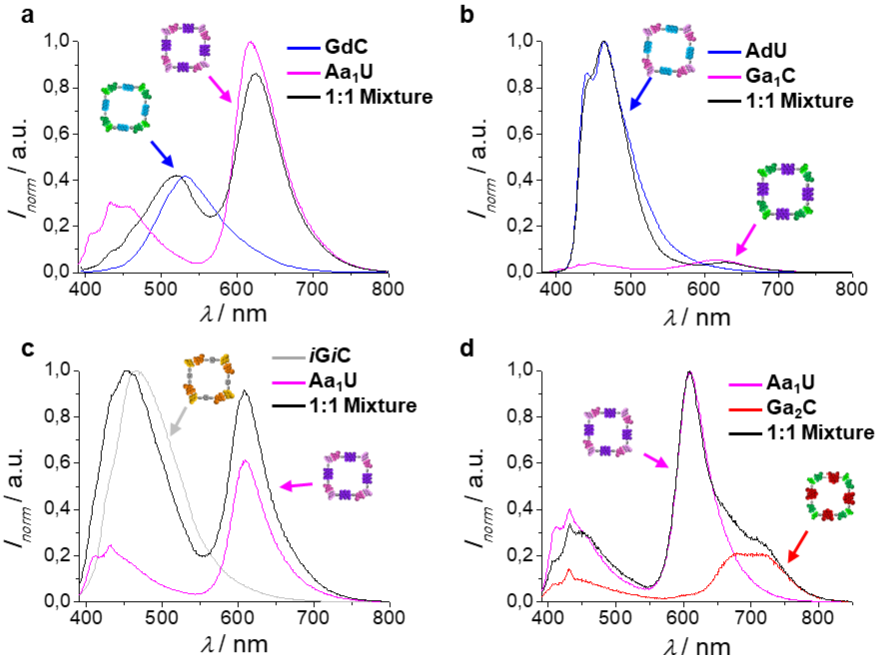


**Figure S3C**. *Evaluation of self-sorting in 1:1 dinucleoside mixtures with different nucleobase pairs.* (**a**) Emission spectra ($\lambda_{exc}$ = 381 nm) of **GdC**, **Aa₁U** and their combination. (**b**) Emission spectra ($\lambda_{exc}$ = 360 nm) of **AdU**, **Ga₁C** and their combination. (**c**) Emission spectra ($\lambda_{exc}$ = 381 nm) of ***iGiC***, **Aa₁U** and their combination. (**d**) Emission spectra ($\lambda_{exc}$ = 381 nm) of **Aa₁U**, **Ga₂C** and their combination. The concentration of each dinucleoside in toluene was 5.0·$10^{-5}$ M (**a**,**b**) or 2.0·$10^{-5}$ M (**c**,**d**) and *T* = 298 K.

The question now arises whether a donor-acceptor *i*G-*i*C + G-C mixture, having nucleobase pairs that do not promote self-sorting, would self-sort as well in the corresponding cyclic tetramers, as NOESY NMR experiments demonstrated. These experiments had to be performed with compound ***iGiC*** as donor and in $CHCl_3$ as solvent, due to the low solubility found in general for iG-iC dinucleosides, as noted above in the NMR measurements. Anyways, Figure S3Da shows that when ***iGiC*** and **Ga₁C** dinucleosides are combined, their equilibrium mixture exhibits virtually the same spectroscopic features as the sum of the spectra when these samples are analyzed separately. This is also the case when ***iGiC*** and **Ga₂C** are combined (Figure S3Db). This implies that *i*G-*i*C and G-C dinucleosides self-associate independently in their corresponding cyclic tetramers and no mixed assemblies, where G would bind to *i*C or *i*G to C, are formed.

In short, these experiments using optical spectroscopy and dyes that absorb and emit in different spectral regions also support the notion that *narcissistic self-sorting is primarily governed by the strong chelate cooperativity manifested by each dinucleoside monomer when assembled as a cyclic tetramer*.

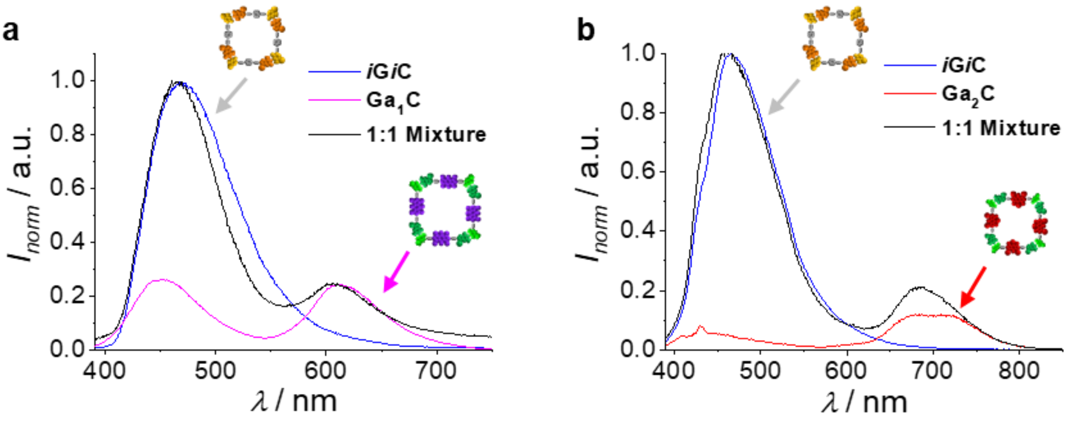


**Figure S3D**. *Evaluation of self-sorting in dinucleoside mixtures with different nucleobase pairs.* (**a**) Emission spectra ($\lambda_{exc}$ = 381 nm) of ***iGiC*** (*C* = 7.5·10$^{-5}$ M), **Ga$_1$C** (*C* = 1.5·10$^{-4}$ M) and their combination at the same concentrations in CHCl$_3$. (**b**) Emission spectra ($\lambda_{exc}$ = 381 nm) of ***iGiC*** (*C* = 2.0·10$^{-5}$ M), **Ga$_2$C** (*C* = 2.0·10$^{-5}$ M) and their combination at the same concentrations in toluene. *T* = 298 K.

Finally, once the study of self-sorting processes of binary mixtures of dinucleosides was completed and understood, we proceeded with the analysis of more complex ternary mixtures of the G-C, A-U and iG-iC dinucleosides. For such goal, we wanted to employ the three chromophores: **d**, **a$_1$** and **a$_2$** that absorb and emit in different regions of the spectrum and that constitute two pairs of FRET couples. Due to its higher association strength and high solubility and reliability, we decided to install **a$_2$** in the G-C scaffold (**Ga$_2$C**), while, for solubility reasons as mentioned above, ***iGdiC*** was substituted by ***iGiC***, which actually presented very similar absorption and emission features, only slightly blue-shifted with respect to ***iGdiC***. Hence the actual ternary mixture was ***iGiC*** + **Aa$_1$U** + **Ga$_2$C**, which, as clearly shown in Figure S3Ea, displayed virtually the same emission spectrum as the sum of the spectrum of the three components. This indicates, as demonstrated for the binary mixtures, that due to the strong self-sorting phenomena induced by the high chelate cooperativities of these systems, the three dinucleoside molecules can be mixed and each of them will associate independently in the corresponding cyclic tetramer. As a control experiment, **d**, **a$_1$** and **a$_2$** were mixed in dinucleosides with the same complementary nucleobases at the edges, namely **GdC** + **Ga$_1$C** + **Ga$_2$C**. As shown in Figure S3Eb, this ternary mixture exhibits substantial quenching of the **GdC** chromophore emission, weaker quenching of **Ga$_1$C** emission, and significant emission enhancement of **Ga$_2$C**, which strongly suggests that a mixture of all possible macrocycles is formed in solution where donors and acceptors are combined in the same assembly and FRET is activated.

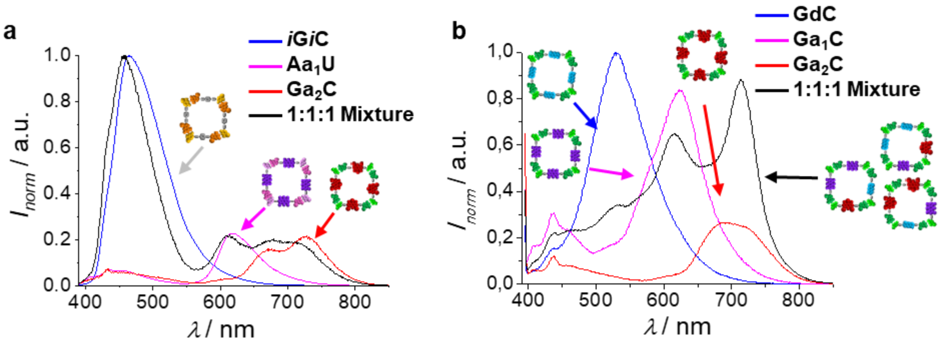


**Figure S3E**. *Evaluation of self-sorting in ternary 1:1:1 dinucleoside mixtures with different nucleobase pairs.* (**a**) Emission spectra ($\lambda_{exc}$ = 381 nm) of ***iGiC***, **Aa$_1$U**, **Ga$_2$C** and their ternary combination at the same concentrations (*C* = 5.0·10$^{-5}$ M) in toluene. (**b**) Emission spectra ($\lambda_{exc}$ = 386 nm) of **GdC**, **Ga$_1$C**, **Ga$_2$C** and their ternary combination at the same concentrations (*C* = 2.0·10$^{-5}$ M) in toluene. *T* = 298 K.

## S4. Selective Dissociation Studies

As stated in the main text, previous work performed in our group concluded that the thermodynamic stability of the *c***AU**$_4$ macrocycle is considerably lower than that of the *c***GC**$_4$ and *ci***G***i***C**$_4$ analogues due to both a weaker binding strength between complementary bases and a reduced chelate cooperativity stemming from the symmetric nature of the *DAD-ADA* H-bonding pattern.[4,5] We hence reasoned that gradually taking the binary or ternary systems to conditions where association is disfavored, either by a decrease in concentration, an increase in temperature or by addition of a polar cosolvent, would result in the selective and sequential dissociation of the cyclic tetramers as a function of their relative thermodynamic stability. In this section, we collect a number of experiments that demonstrate this idea through diverse spectroscopies.

For instance, in temperature-dependent experiments in $CDCl_3$ within the 253-323 K range (see Figure S4Aa), only the *c***AU**$_4$ macrocycle is dissociated at high temperatures, whereas *c***GC**$_4$ remains intact in the whole temperature range. This is clearly evidenced in the disappearance of the H-bonded U-imide and A-amine proton signals at 14.0 and 8.6 ppm, and the concomitant appearance of the solvent-bound U-imide proton signal at around 11-10 ppm. A very similar result was observed by changing solvent composition. Addition of *DMSO-*$D_6$ to (2:3) $CDCl_3$:$CCl_4$ solutions of **GC**+**AU** mixtures led to the observation of two clear regimes (Figure S4Ab). In the first one, from 0 to 12% v/v of DMSO-$D_6$, *c***AU**$_4$ is progressively dissociated in the presence of the stronger *c***GC**$_4$ macrocycle, which show no sign of denaturation. This is evidenced by the appearance of the **AU** monomer U-imide signal at *ca.* 11.8 ppm. In the second regime, starting over *ca.* 20% DMSO-$D_6$, *c***GC**$_4$ is then dissociated to the monomeric species, showing a G-amide signal at 10.9 ppm. It should be remarked that both cyclic tetramers are in slow exchange in the NMR timescale with their respective monomeric species, and that no other associated species is detected in these experiments, which highlights the extraordinarily strong cooperativity of the cyclotetramerization process. Figure S4Ac shows the same DMSO-$D_6$ titrations with the **GC**+***i*****G*****i*****C** mixture. In this case, due to the similar $K_a$ and *EM* values displayed by *c***GC**$_4$ and *ci***G***i***C**$_4$,[4] cyclic tetramer dissociation occurs in parallel, and both **GC** and ***i*****G*****i*****C** monomers are detected in slow exchange at *ca.* 10.8 ppm after a DMSO-$D_6$ volume fraction of 80%.

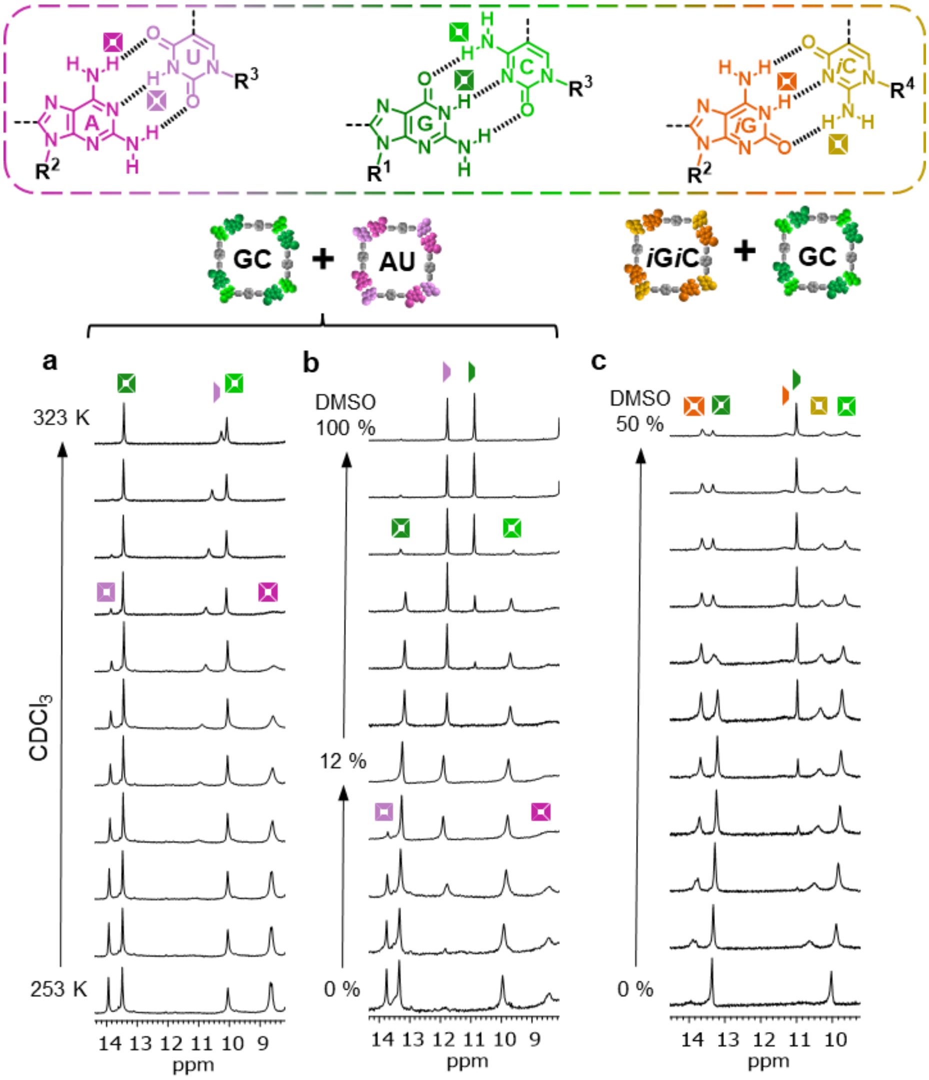


**Figure S4A**. *Selective cyclic tetramer denaturation experiments.* Downfield region of the $^{1}H$ NMR spectra of (**a**) Temperature-dependent measurements of **GC** + **AU** in $CDCl_3$, (**b**) **GC** + **AU** in $CDCl_3$:$CCl_4$ (2:3) with increasing DMSO-$D_6$ content, and (**c**) **GC** + ***iGiC*** in $CDCl_3$ with increasing DMSO-$D_6$ content, showing the slow exchange between tetramer (squares) and monomer (rods) proton signals. Initially, the $^{1}H$ signals of the *c*(***iGiC***)$_4$ species are broad due to strong aggregation in pure $CDCl_3$. A small amount of DMSO needs to be added to achieve complete solubility.

Furthermore, we could monitor the dissociation of the weaker A-U cyclic tetramers in the presence of the stronger G-C or iG-iC macrocycles by CD spectroscopy as a function of the temperature. Figures S4Ba and b display the temperature-dependent CD spectra of the **GdC**+**Aa$_1$U** and **AdU**+**Ga$_1$C** mixtures, respectively. At low temperatures each macrocycle produces a characteristic Cotton effect around their respective absorption maxima. As the temperature is increased, the macrocycles that are held together by A:U interactions (either *c*(**AdU**)$_4$ or *c*(**Aa$_1$U**)$_4$) are dissociated and the CD signal in the corresponding region disappears, whereas the *c*(**GdC**)$_4$ or *c*(**Ga$_1$C**)$_4$ are not significantly affected. This selective dissociation does not occur, on the contrary, when macrocycles of similar stability are mixed, like *c*(**iGiC**)$_4$ and *c*(**Ga$_1$C**)$_4$. As shown in Figure S4Bc, the CD spectrum of this mixture remains invariable in the studied concentration range due to the high thermodynamic stability of the *c*(***iGiC***)$_4$ and *c*(**Ga$_1$C**)$_4$ assemblies.

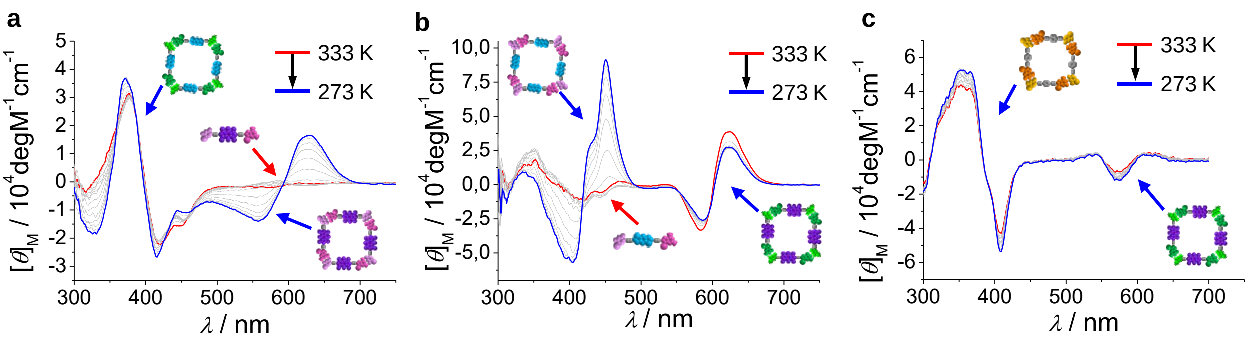


**Figure S4B**. *Selective dissociation of the weakest cyclic tetramer in a self-sorted mixture as a function of temperature.* (**a**) CD spectra of the **GdC** + **Aa$_1$U** mixture at different temperatures. (**b**) CD spectra of the **AdU** + **Ga$_1$C** mixture at different temperatures. (**c**) CD spectra of the **iGiC** + **Ga$_1$C** mixture at different temperatures. The concentration of each dinucleoside was $5.0\cdot10^{-5}$ M in toluene (**a**,**b**) and $7.5\cdot10^{-5}$ (***iGiC***) and $7.5\cdot10^{-5}$ M (**Ga$_1$C**) in $CHCl_3$ (**c**).

Finally, we studied the same ***iGiC*** + **Aa$_1$U** + **Ga$_2$C** ternary mixture as before (see Figure S3E and main text) by variable-temperature CD spectroscopy (Figure S4Ca). As expected, only the weaker **Aa$_1$U** cyclic tetramers is dissociated at high temperatures, while the other two ***iGiC*** and **Ga$_2$C** macrocycles resist. In the control **GdC** + **Ga$_1$C** + **Ga$_2$C** mixture (Figure S4Cb), however, the CD spectra remains invariable because all macrocycles present a similarly high stability and do not break under these conditions.

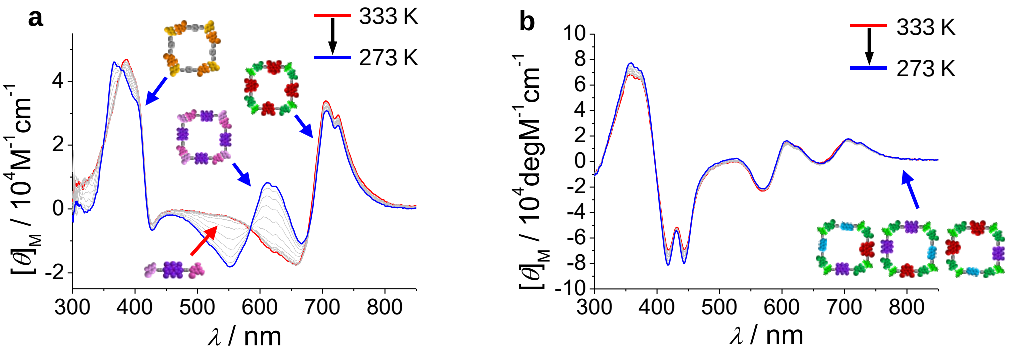


**Figure S4C**. *Selective dissociation of the weakest cyclic tetramer in a self-sorted mixture as a function of temperature.* (**a**) CD spectra of the ***iGiC*** + **Aa$_1$U** + **Ga$_2$C** ternary mixture at different temperatures. (**b**) CD spectra of the **GdC** + **Ga$_1$C** + **Ga$_2$C** mixture at different temperatures. All dinucleosides have the same concentration ($C$ = $2.0\cdot10^{-5}$ M) in toluene. $T$ = 298 K.

## S5. Self-sorting in mixtures of mono- and dinucleosides.

We next examined if self-sorting occurred in a mixture of mononucleosides and dinucleosides that share the *same* Watson Crick H-bonding interaction. We selected two systems of very different cooperativity: $c$(AU)$_4$ ($K_{AU}$ ($CDCl_3$) ~ 3·10$^2$ M$^{-1}$; $EM_{AU}$ ~ 10$^{-1}$-10$^{-2}$ M) and $c$(GC)$_4$ ($K_{GC}$ ($CDCl_3$) ~ 3·10$^4$ M$^{-1}$; $EM_{GC}$ ~10$^2$-10$^3$ M), and combined them with 1:1 mixtures of the corresponding A+U and G+C mononucleosides.

Figure S5A shows titration experiments in which 1:1 mixtures of the complementary mononucleosides (i.e. **A**+**U** or **G**+**C**) were added to the corresponding dinucleosides (**AU** or **GC**), which, in the starting conditions, are associated as cyclic tetramers ($c$(**AU**)$_4$ or $c$(**GC**)$_4$) in slow NMR exchange with the rest of supramolecular species. These experiments were made in a $CDCl_3$:$CCl_4$ (2:3) solvent mixture for **AU**, and in THF-$D_8$ for **GC**. These solvent systems were chosen so as to regulate the association constant ($K$) of the corresponding Watson-Crick pairs and maintain an adequate population of associated species within the concentration range studied. Along these titrations, the **AU**/**GC** concentration and the temperature are kept constant. As shown in Figure S5A, the cyclic tetramer is seen to disappear as increasing amounts of mononucleosides are added and mixed, non-sorted associated species, like **U:AU**, **AU:A** or **U:AU:A**, are formed, which coexist in fast exchange with other non-cyclic oligomers, the **A**:**U** pair, and dissociated **A** and **U**. The same applies to the mixture **GC**+**G**+**C**. The difference between the two dinucleosides is the amount of 1:1 mononucleoside mixture required to fully destroy the cyclic self-sorted assembly. This is lower than 3 equivalents for $c$(**AU**)$_4$, whereas $c$(**GC**)$_4$ can resist up to *ca.* 25 equivalents. This means that the intra- and intermolecular versions of the G:C Watson-Crick pair can indeed coexist in solution without much interference, giving rise to self-sorted assemblies, as long as the relative amount of competing mononucleoside mixture is not too high.

Figure 5B shows the changes observed in the $^1$H NMR spectra of a 1:1:1 **AU**+**A**+**U** and a 1:2:2 **GC**+**G**+**C** mixture as a function of temperature in $CDCl_3$:$CCl_4$ (2:3) and THF-$D_8$, respectively. As it happens for the $c$(**AU**)$_4$ macrocycle alone,[4] as the temperature increases the cycle is dissociated into short, non-cyclic (**AU**)$_n$ oligomers and **AU** monomer, which are in fast exchange between themselves, and in these conditions also with mixed species like **U:AU**, **AU:A** or **U:AU:A**, the **A**:**U** pair, and dissociated **A** and **U**. This is observed in a progressive intensity decay of the characteristic $c$(**AU**)$_4$ $^1$H signals, obtained by integration and represented in the graph at the right side, at the expense of the non-sorted mixture of species. Also, as the temperature increases, the abundance of dissociated **AU**, **A** and **U** increases, and the signals corresponding to this fast-exchanging mixture shift upfield. For the **GC**+**G**+**C** mixture, due to the much higher stability of the $c$(**GC**)$_4$ macrocycle, its relative population remains constant even as the temperature is increased and it is only the **G**:**C** pair that is seen to dissociate, since the signals for this complex shift upfield with temperature. Hence, due to the sufficiently strong chelate cooperativity of $c$(**GC**)$_4$, we can selectively break the intermolecular association without affecting the self-sorted, intramolecularly bound species.

Figure 5C displays different NOESY spectra taken at diverse mixing times ($\tau_m$) for the same 1:1:1 **AU**+**A**+**U** and 1:2:2 **GC**+**G**+**C** mixtures. At sufficiently long mixing times, the exchange cross-peaks between $c$(**AU**)$_4$ and the fast-exchanging mixture of species are observed, and an exchange rate constant was calculated as 1.8 s$^{-1}$ using the expression:[3]

$$k = \frac{1}{\tau_m} \ln \frac{r+1}{r-1} \qquad r = 4X_A X_B \frac{I_{AA} + I_{BB}}{I_{AB} + I_{BA}} - (X_A - X_B)^2$$

where $k$ is the exchange rate constant, $\tau_m$ is the mixing time, $X_A$ and $X_B$ are the molar fractions of molecules in states A and B, respectively, $I_{AA}$ and $I_{BB}$ are the diagonal peak intensities, and $I_{AB}$ and $I_{BA}$ are the cross-peak intensities. However, in the **GC**+**G**+**C** mixture no exchange cross-peaks could be detected even at the longest mixing times, which highlights the kinetic stability of the self-sorted *c*(**GC**)$_4$ + **G**:**C** mixture.

Finally, Figure S5D displays the DOSY NMR spectra of the same 1:1:1 **AU**+**A**+**U** and 1:2:2 **GC**+**G**+**C** mixtures, where two sets of diffusing species in slow exchange are clearly seen:

1) The *c*(**AU**)$_4$ and *c*(**GC**)$_4$ macrocycles, which are larger in size and thus display smaller diffusion coefficients.

2) The mixture of fast-exchanging oligomers (for **AU**) or the **G**:**C** pair (for **GC**).

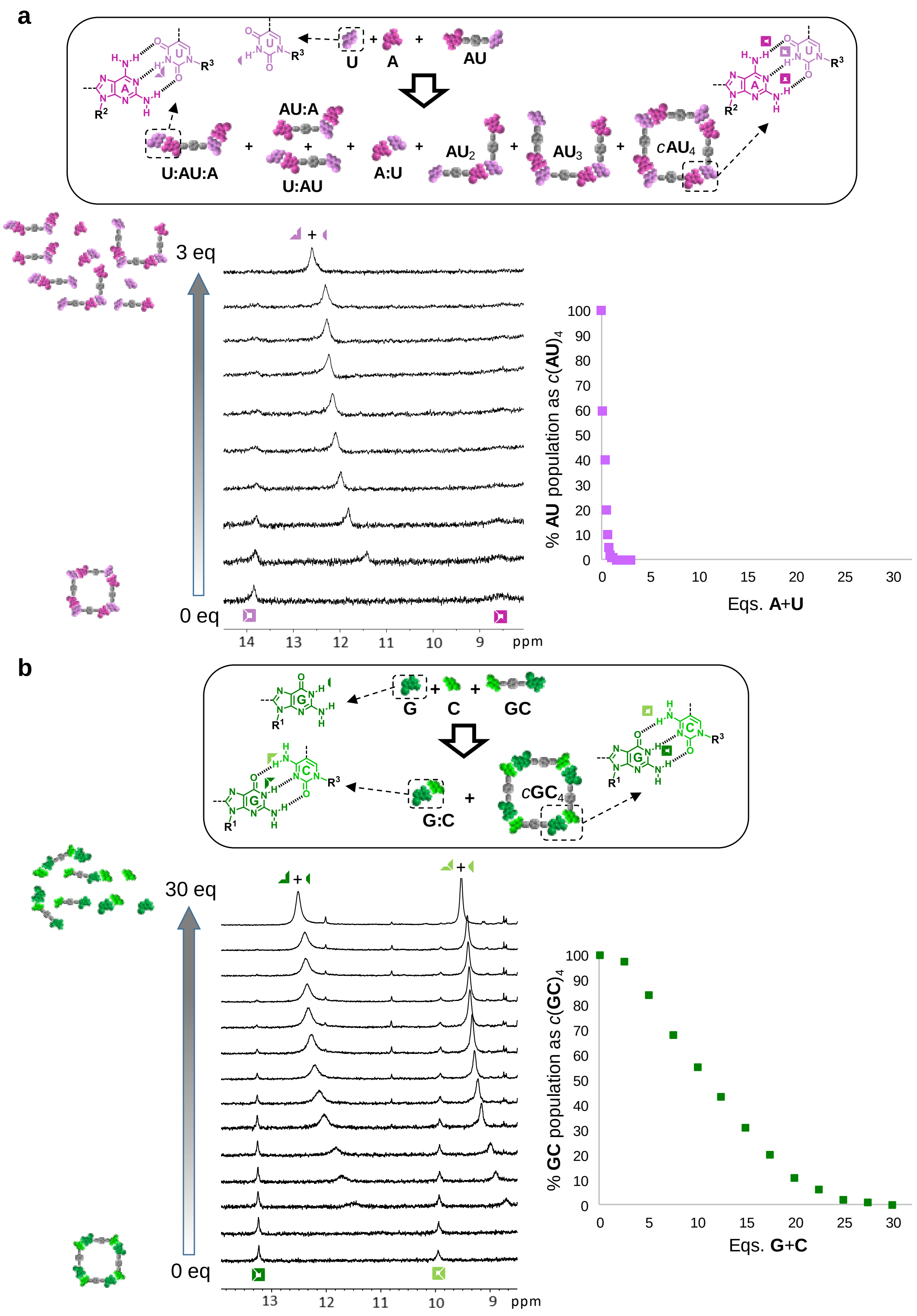


**Figure S5A**. *Titration of the dinucleoside, initially associated as cyclic tetramers, with increasing amounts of the corresponding 1:1 mixture of complementary mononucleosides.* (**a**) **AU** with **A** + **U** in $CDCl_3$:$CCl_4$ (2:3); (**b**) **GC** with **G** + **C** in THF-$D_8$. In both cases, the *ca.* 8-15 ppm region of the $^1$H NMR spectra is shown, where the most relevant H-bonded proton signals are found. At the right, the abundance of dinucleoside molecules associated as cyclic tetramers is represented as a function of the equivalents of 1:1 mononucleoside mixture added.

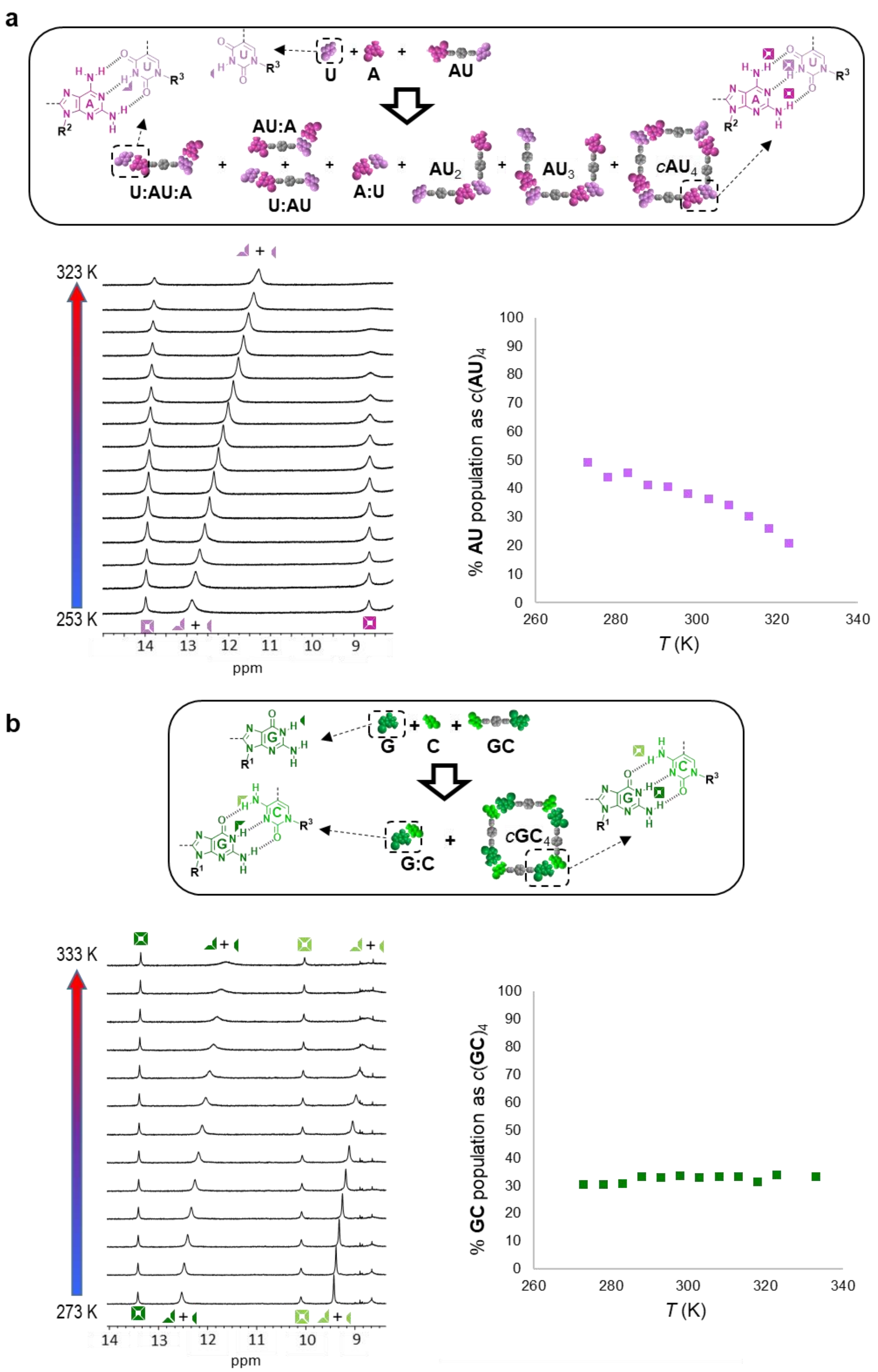


**Figure S5B**. *Temperature dependent behaviour of mixtures of dinucleoside and the corresponding mononucleosides.* (**a**) A 1:1:1 mixture of **AU** + **A** + **U** in $CDCl_3$:$CCl_4$ (2:3); (**b**) a 1:2:2 mixture of **GC** + **G** + **C** in THF-$D_8$. In both cases, the *ca.* 8-15 ppm region of the $^1H$ NMR spectra is shown, where the most relevant H-bonded proton signals are found. At the right, the abundance of dinucleoside molecules associated as cyclic tetramers is represented as a function of the temperature.

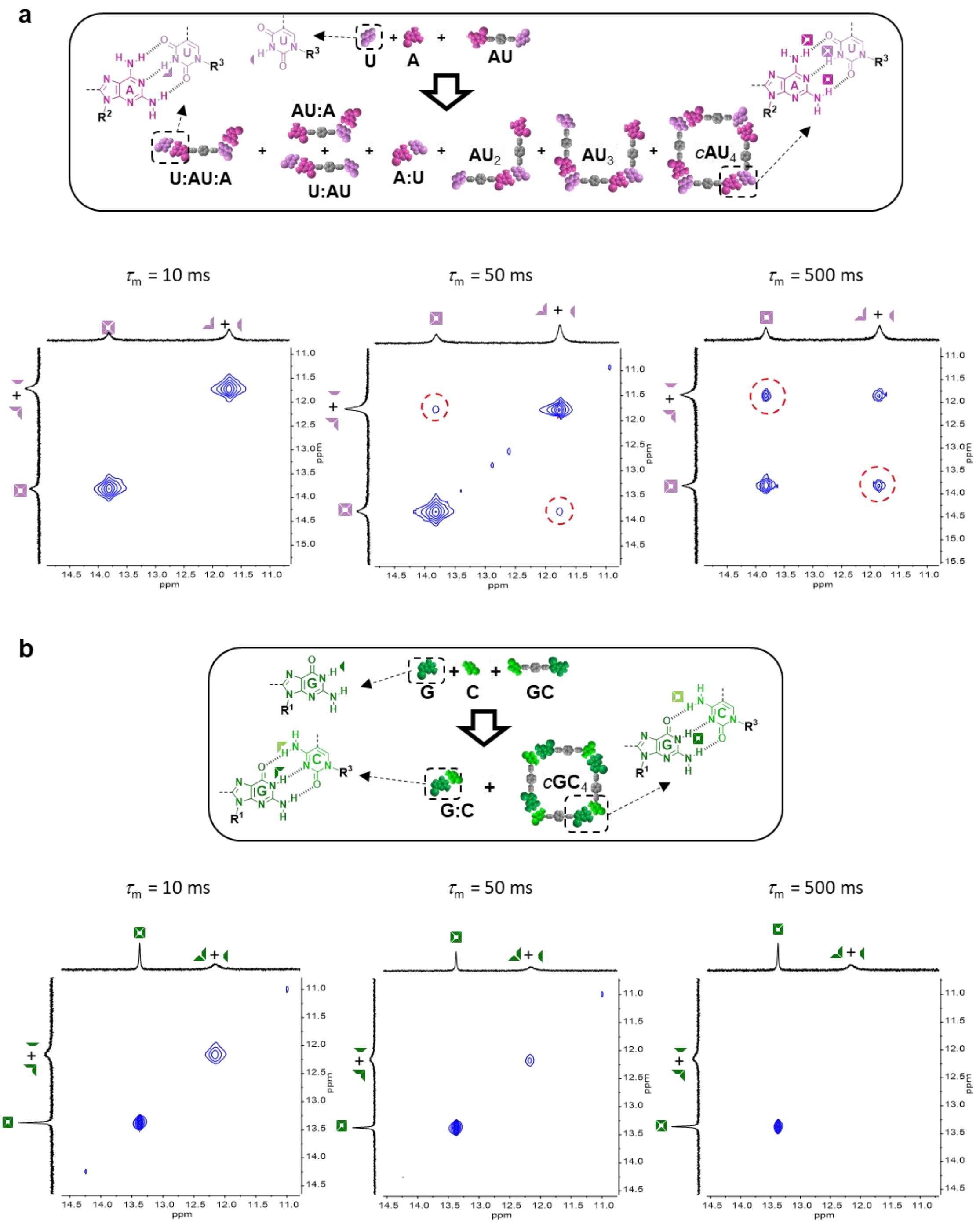


**Figure S5C**. *Exchange dynamics of the dinucleoside associated as cyclic tetramers and as open oligomers.* NOESY NMR spectra at different mixing times ($\tau_m$) of (**a**) A 1:1:1 mixture of **AU** + **A** + **U** in $CDCl_3$:$CCl_4$ (2:3); (**b**) a 1:2:2 mixture of **GC** + **G** + **C** in THF-$D_8$. In both cases, the *ca.* 11-15 ppm region of the $^1$H NMR spectra is shown, where the U-imide and G-amide proton signals can be found.

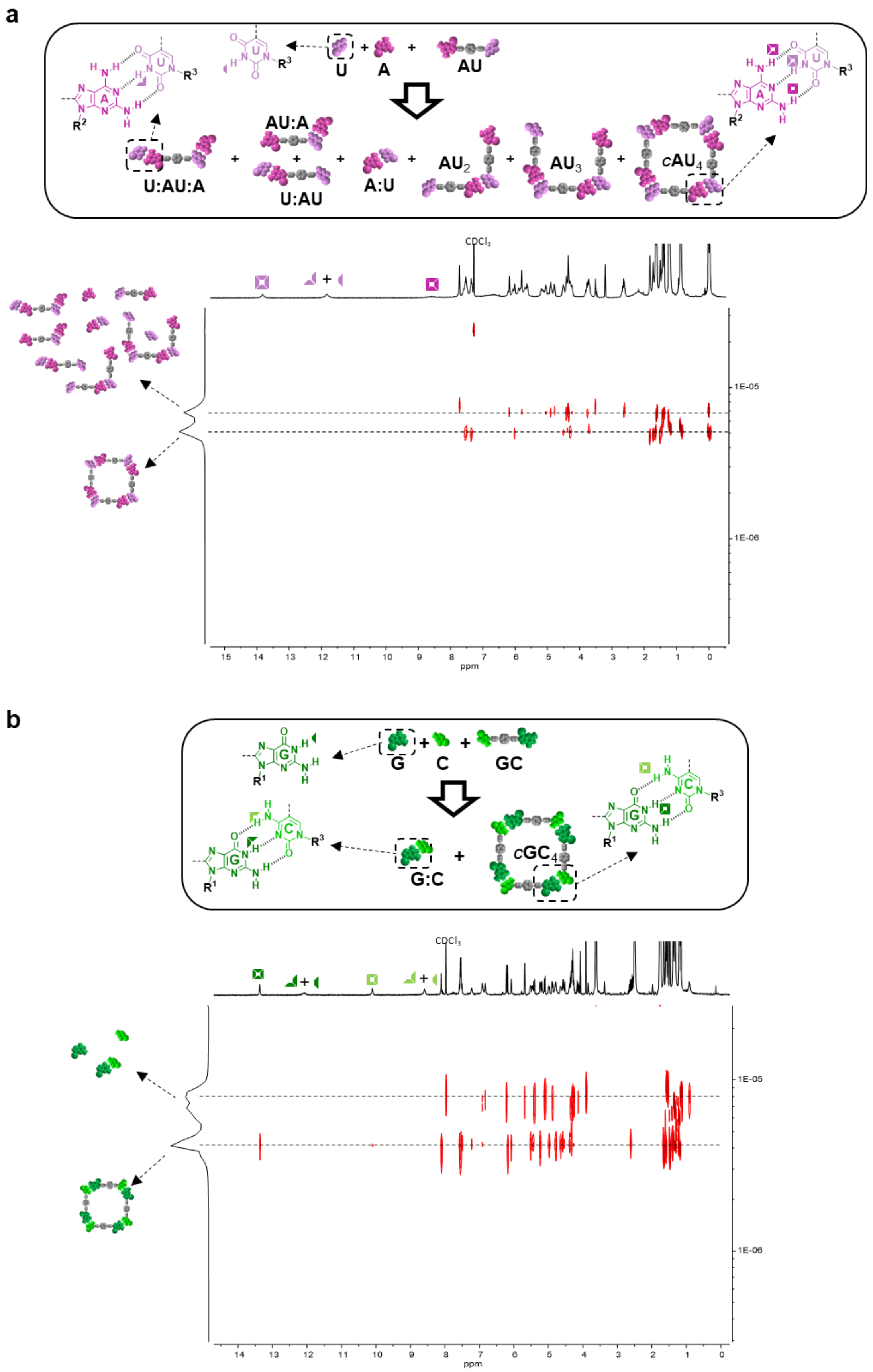


**Figure S5D**. *Diffusion of the mixture of dinucleoside and complementary mononucleosides.* DOSY NMR spectra of (**a**) A 1:1:1 mixture of **AU** + **A** + **U** in $CDCl_3$:$CCl_4$ (2:3); (**b**) a 1:2:2 mixture of **GC** + **G** + **C** in THF-$D_8$.
